# Glyoxal oxidase-mediated detoxification of reactive carbonyl species contributes to virulence, stress tolerance, and development in a pathogenic fungus

**DOI:** 10.1371/journal.ppat.1012431

**Published:** 2024-07-30

**Authors:** Xiaoyu Liu, Nemat O. Keyhani, Hong Liu, Yue Zhang, Yuxian Xia, Yueqing Cao

**Affiliations:** 1 Genetic Engineering Research Center, School of Life Sciences, Chongqing University, Chongqing, People’s Republic of China; 2 Chongqing Engineering Research Center for Fungal Insecticide, Chongqing, People’s Republic of China; 3 Key Laboratory of Gene Function and Regulation Technologies Under Chongqing Municipal Education Commission, Chongqing, People’s Republic of China; 4 Department of Biological Sciences, University of Illinois, Chicago, Illinois, United States of America; Universidade de São Paulo Câmpus de Ribeirão Preto: Universidade de Sao Paulo Campus de Ribeirao Preto, BRAZIL

## Abstract

Reactive carbonyl and oxygen species (RCS/ROS), often generated as metabolic byproducts, particularly under conditions of pathology, can cause direct damage to proteins, lipids, and nucleic acids. Glyoxal oxidases (Gloxs) oxidize aldehydes to carboxylic acids, generating hydrogen peroxide (H_2_O_2_). Although best characterized for their roles in lignin degradation, Glox in plant fungal pathogens are known to contribute to virulence, however, the mechanism underlying such effects are unclear. Here, we show that Glox in the insect pathogenic fungus, *Metarhizium acridum*, is highly expressed in mycelia and during formation of infection structures (appressoria), with the enzyme localizing to the cell membrane. *MaGlox* targeted gene disruption mutants showed RCS and ROS accumulation, resulting in cell toxicity, induction of apoptosis and increased autophagy, inhibiting normal fungal growth and development. The ability of the *MaGlox* mutant to scavenge RCS was significantly reduced, and the mutant exhibited increased susceptibility to aldehydes, oxidative and cell wall perturbing agents but not toward osmotic stress, with altered cell wall contents. The Δ*MaGlox* mutant was impaired in its ability to penetrate the host cuticle and evade host immune defense resulting in attenuated pathogenicity. Overexpression of *MaGlox* promoted fungal growth and conidial germination, increased tolerance to H_2_O_2_, but had little to other phenotypic effects. Transcriptomic analyses revealed downregulation of genes related to cell wall synthesis, conidiation, stress tolerance, and host cuticle penetration in the Δ*MaGlox* mutant. These findings demonstrate that *MaGlox*-mediated scavenging of RCS is required for virulence, and contributes to normal fungal growth and development, stress resistance.

## Author summary

The ability of organisms to detoxify reactive metabolic byproducts is crucial for normal development, stress resistance, and where applicable, pathogenesis. Here, using the insect pathogenic fungus, *Metarhizium acridum*, we show that a glyoxal oxidase acts to detoxify cellular reactive carbonyl species (RCS) as well as leading to decreased reactive oxygen species (ROS) levels by alleviating aldehydes accumulation which can trigger ROS production. Mutants deficient in *MaGlox* showed increased levels of RCS/ROS leading to increased autophagy and apoptosis, resulting in impaired fungal growth, development, and reduced virulence. These findings link Glox mediated RCS/ROS detoxification to cell degradation/turnover processes and cell death, impacting fungal pathogenicity and development.

## Introduction

Glyoxal oxidases (Glox) are copper-containing extracellular or membrane bound enzymes with broad substrate specificities capable of oxidizing aldehydes into carboxylic acids coupled to the conversion of oxygen to the production of hydrogen peroxide (H_2_O_2_) [1,2]. Glox belongs to the AA5_1 subfamily, composed of Glox and glyoxal-like enzymes termed radical copper oxidases, and is related in structure and reactive chemistry to galactose oxidases, although the latter are considered as a separate subfamily (AA5_2) [3–5]. Gloxs contain a glyoxal oxidase domain and from zero to five cell wall integrity and stress component domains (WSC domains) [6,7]. Glox was first characterized in the cellulose-degrading (white rot) fungus *Phanerodontia chrysosporium* [8] where it is a critical component involved in the breakdown of lignin [1,7,9,10]. The enzyme converts aldehydes produced from the pretreatment of lignocellulose, *e*.*g*., furaldehyde and 5-(hydroxymethyl)-2-furaldehyde (HMF), producing H_2_O_2_ [11], and the biochemical (and hence biological) roles of Gloxs have mainly focused on their contributions to substrate degradation in lignocellulose-degrading fungi white-rot fungi and plant associated *Trichoderma* sp., as well as its potential biotechnological application in bioethanol production (in yeasts) [11–13]. Several Glox enzymes have also been characterized in plant pathogenic fungi. Glox has been shown to contribute to virulence and mycotoxin production in the wheat and barley blight pathogen, *Fusarium graminearum* [14], and is involved in mycelial development and pathogenicity in the corn smut causing fungus, *Ustilago maydis* [15]. Downregulation of *Glox*1 in *Trichoderma virens*, a soil dwelling saprophyte currently being used as a biological control agent against plant microbial pathogens such as *Pythium* and *Rhizoctonia* sp., resulted in delayed hyphal growth and reduced hydrophobicity of conidia, but had no effect on its biocontrol ability [13]. Interestingly, Glox enzymes are also found in plants, and a Glox gene in grapevine (*Vitus pseudoreticulata*) appears to help defend the plant against the powdery mildew fungal pathogen, *Erysiphe necator*, by producing toxic H_2_O_2_ [16]. However, the functions of *Glox* genes in fungal animal or insect pathogens has yet to be examined.

Entomopathogenic fungi are increasingly being utilized as safe and environmentally-friendly alternatives to chemical insecticides for pest control, as agents that can be compatible with a variety of other pest management strategies as well as “green” farming practices [17,18]. The infection process of these fungi, *e*.*g*. those from the *Metarhizium* genera, towards host insects typically involves spore adhesion, appressorium formation, cuticle penetration, colonization in the hemocoel, and sporulation on the host cadavers [19,20]. During infection, entomopathogenic fungi are challenged by various biotic and abiotic stresses, including oxidative and osmotic stresses, unfavorable temperature and moisture, poor nutrition, antagonistic microbes on the cuticle surface, and the host innate immunity [21,22]. These factors can induce the generation of reactive oxygen species (ROS), which can damage macromolecules and promote autophagy and apoptosis [23,24]. Endogenous factors, which encompass the accumulated intermediate products in metabolism involving specific oxidases, also have the potential to induce the production of ROS [24–26]. Aldehydes, classified as carbonyl compounds, hold a significant status as reactive carbonyl species (RCS). They can cause extensive damage to cellular biomolecules and exhibit various biological effects, ranging from disrupting normal gene regulation to toxicity [27]. As Glox enzymes mediate aldehyde oxidation we hypothesized that (in non-lignin degrading fungi), these enzymes may be primarily used for detoxification of RCS while potentially producing ROS that then contributes to targeting host defenses and hence is required for virulence and normal fungal growth.

Although many *Metarhizium* species are broad host range insect pathogens (*e*.*g*., *M*. *robertsii*), *M*. *acridum* has a narrower host specificity towards acridids, and has been commercialized for use in killing locusts and grasshoppers [28,29]. Several unique adaptions have been reported for *M*. *acridum* including expression of a specialized extracellular catalase-peroxidase implicated in scavenging host antimicrobial oxidative stress defenses [22]. In order to further probe fungal detoxification mechanisms that might contribute to pathogenicity and development, we investigated the function of the *Glox* gene in *M*. *acridum* via genetic and biochemical approaches. Our results indicate that MaGlox scavenges RCS by targeting aldehyde accumulation, influencing numerous downstream physiological and biological pathways including, ROS accumulation, fungal growth and development, apoptosis, stress tolerance, and pathogenicity.

## Results

### MaGlox sequence analyses, cellular localization and gene expression pattern

A single *Glox* homolog, with a full-length nucleotide sequence of 3762 bp, containing three introns was identified in the *M*. *acridum* genome. The open reading frame of *MaGlox* was 3093 bp, encoding a protein of 1030 amino acids with an estimated molecular weight of 108.16 kDa and an isoelectric point of 4.97 (https://web.expasy.org/protparam/). Sequence analyses indicated that the protein contained eight transmembrane helices (at the C-terminus) as predicted by TMpred, an N-terminal signal peptide of 23 amino acids, four WSC domains at the N-terminus, a conserved glyoxal oxidase N-terminal domain (Glyoxal_oxid_N) (from Val-583 to Gly-776) and a C-terminal early “set” domain (E_set_GO_C) (from Gly-911 to Ile-1009, S1A Fig). The Glyoxal_oxid_N and E_set_GO_C domains grouped together (referred to as the AA5_1 domain) and are conserved in all Glox proteins. Sequence and domain alignments confirmed the absence of WSC domains in plant Gloxs, and in several white-rot fungi and human pathogenic fungi, whereas three to five WSC domains were found in phytopathogenic fungi including *Trichodema* spp., *Thermothelomyces thermophilus*, and in all Ascomycete entomopathogenic fungi examined (S1B Fig). Phylogenetic analysis revealed that MaGlox grouped within a clade containing both microbial and insect biocontrol fungi, *e*.*g*., *Metarhiuzuim* sp., *Beauveria bassiana* and *Trichoderma* sp. (S1B Fig). Sequence alignment showed that MaGlox had the five conserved active site residues (Cys-589, Tyr-648, Tyr-870, His-871 and His-955) within the AA5_1 domain (S1C Fig).

**Fig 1:**
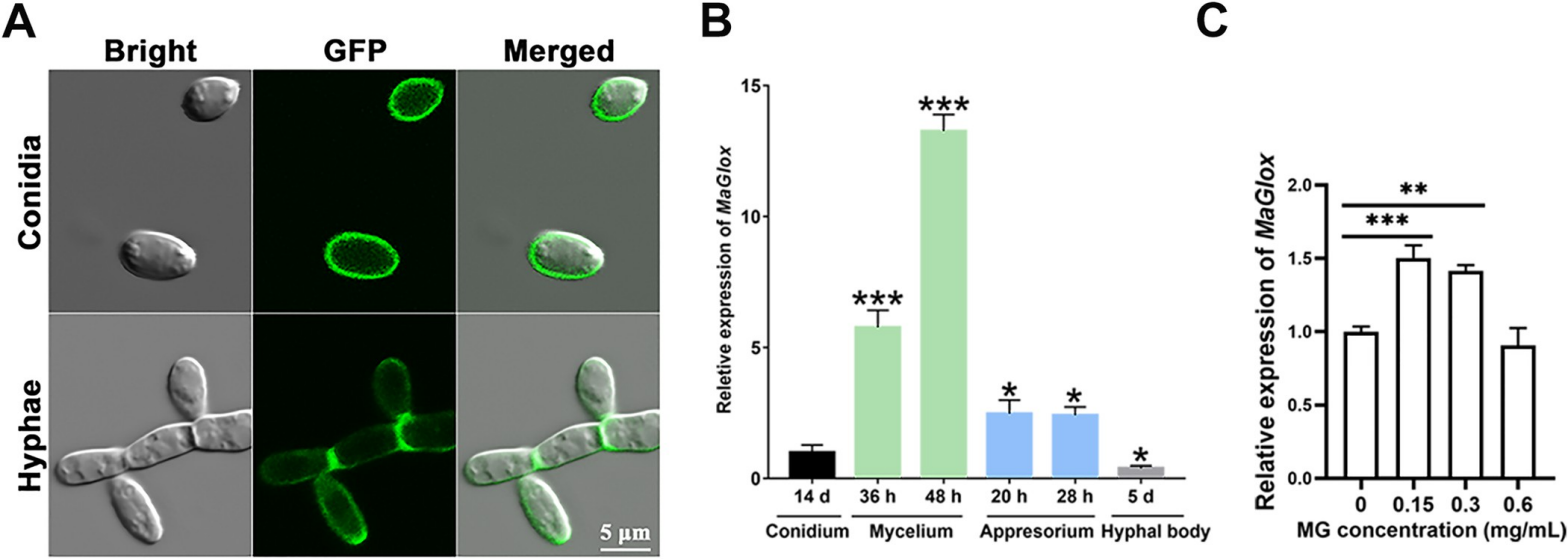
Subcellular localization MaGlox and *MaGlox* relative expression levels in different fungal tissues. (A) LSCM images of EGFP-tagged MaGlox in hyphae and conidia. Scale bar indicates 5 μm. (B) Expression pattern of *MaGlox* in 14 d conidia, hyphae/mycelia at 36 and 48 h growth in ¼-SDY, appressoria at 20 h and 28 h post inoculation on locust wings, and fungal hyphal bodies derived from insect hemolymph during infection. (C) Effect of exogenous methylglyoxal (MG) on the expression of *MaGlox*. MG at different concentrations was added to *M*. *acridum* 48-h liquid cultural, and the fungus continues to grow 6 h. Transcription level of *MaGlox* was determined by RT-qPCR. Error bars represent the standard deviation. Tukey’s HSD, *: *P* < 0.05; ***: *P* < 0.001.

To examine subcellular localization of the protein, a *MaGlox*-*eGFP* expression fusion construct was transformed into wild type *M*. *acridum* as detailed in the Methods section (S2 Fig and S1 Table). These data showed green fluorescence signals at the cell membrane, particularly at the septa in hyphae, and in the membranes of conidia (Fig 1A). To provide a quantitative analysis of *MaGlox* expression, RT-qPCR analyses were performed with RNA extracted from various fungal cells. These data indicated the highest expression in growing hyphae/mycelia, which was ~5–13 times greater than that seen in conidia (Fig 1B). *MaGlox* expression during appressorium formation on locust wings was twice that seen in conidia, but *MaGlox* expression was low in *in vivo*-derived fungal cells (termed hyphal bodies) growing inside the host hemolymph after cuticle penetration (Fig 1B). Addition of exogenous methylglyoxal at low concentrations (0.15 mg/mL and 0.3 mg/mL) induced expression of *MaGlox* by 40–50% (*P* < 0.01), however, increased expression was not observed at a higher methylglyoxal concentration tested (0.6 mg/mL) (Fig 1C).

### *MaGlox* contributes to growth and conidiation

To investigate functional roles for *MaGlox*, we utilized homologous recombination to construct a targeted gene deletion mutant via replacement of a portion of the *MaGlox* gene with the *bar* gene resistance marker cassette as well as constructing a corresponding complementation strain (CP). We also constructed a constitutive *MaGlox* (over) expression strain (*MaGlox*-OE), in which *MaGlox* expression was driven by the glyceraldehyde-3-phosphate dehydrogenase gene promoter (*PgpdM*) (S2A Fig). The integrity of the *MaGlox* disruption mutant, *MaGlox*-OE and complementation strains were confirmed by PCR and Southern blot analyses (S2B-S2E Fig). As expected, expression of *MaGlox* was undetectable in the Δ*MaGlox* mutant and recovered in the CP strain. *MaGlox* expression was 7 times higher in the *MaGlox*-OE strain as compared to the wild type parent (S2F Fig).

**Fig 2:**
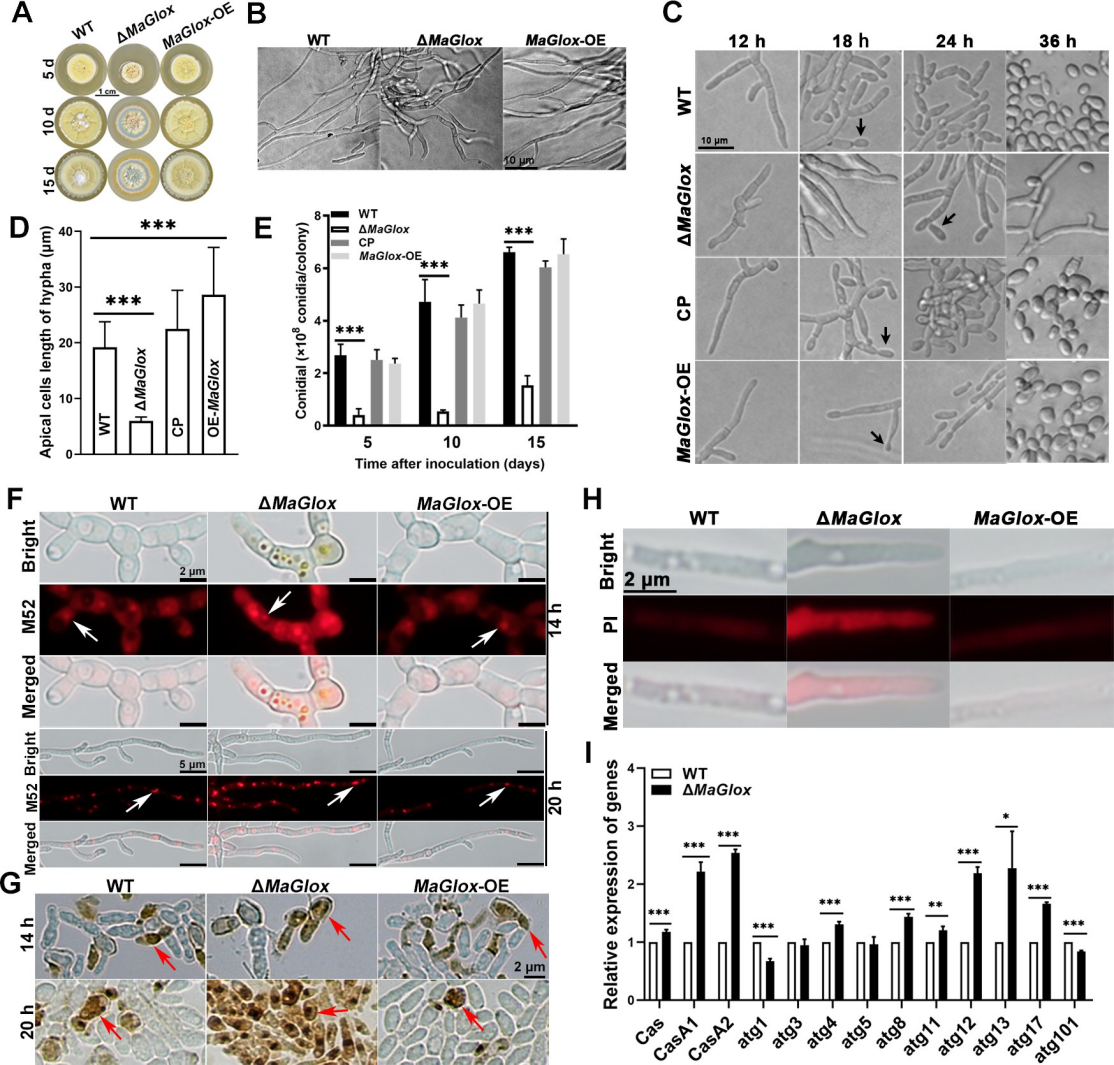
*MaGlox* contributes to autophagy and apoptosis. (A) Colony morphology on ¼-SDAY medium. (B) Hyphae morphology in ¼-SDY liquid medium. (C) The hyphal growth and conidial development of fungal strains at 18 h, 24 h and 36 h. White arrows indicated mature conidia. (D) The apical cell hyphae length of fungal strains at 20 h. (E) Conidial yield of fungal strains grown on ¼-SDAY at 5 d, 10 d and 15 d. Error bars represent standard deviation. Tukey’s HSD, ***: *P* < 0.001. (F) Autophagosomes in hyphae of WT, Δ*MaGlox* and *MaGlox*-OE strains at 14 h and 20 h stained by fluorescent probe perylene-3,4-dicarboxylic anhydride. (G) TUNEL staining in hyphae of WT, Δ*MaGlox* and *MaGlox*-OE strains cultured in ¼-SDY medium at 28°C for 14 h and 20 h. (H) PI staining in hyphae of WT, Δ*MaGlox* and *MaGlox*-OE strains cultured in ¼-SDY medium. (I) Relative expression level of autophagy and apoptosis related genes in WT and Δ*MaGlox* strains. Tukey’s HSD, **: *P* < 0.01; ***: *P* < 0.001.

The Δ*MaGlox* mutant showed a moderate growth inhibition phenotype (12.3–25.7% decrease, *P* < 0.05) when cultured on ¼-SDAY, with the colony color changed from normal yellow green to grey-green and conidial concentric rings more evident as compared to the wild type strain. *MaGlox-*OE strain had a similar phenotype as that of the wild type (Fig 2A). Δ*MaGlox* hyphae were irregular and curled as compared to longer and straighter hyphae seen for the wild type and *MaGlox*-*OE* strain (Fig 2B). The wild type strain initiated conidiation at ~18 h on ¼-SDAY, whereas the hyphae of Δ*MaGlox* only began to produced conidiophores at ~24 h (Fig 2C). Few conidia were apparent for the mutant strain even at 36 h, whereas extensive conidial production was evident for the control strains. *MaGlox*-OE strain also showed delayed conidiation as compared to wild type, with hyphae longer than wild type (Fig 2C). Measurement of the hyphal apical cell length revealed an ~70% reduction in the Δ*MaGlox* and ~ 25% increase in *MaGlox*-OE strain as compared to wild type and complemented controls (*P* < 0.01, Fig 2D). Quantification of conidial yield indicated a 70–90% decrease in conidial production for the Δ*MaGlox* strain over the entire time course examined (5–15 d on ¼-SDAY, *P* < 0.01, Fig 2E). However, no significant differences was found in conidial yield between the *MaGlox*-OE strain and wild type (Fig 2E).

### Loss of *MaGlox* induces autophagy and cellular apoptosis

To examine whether the observed growth phenotypes were related to disruption in normal autophagy and/or cell apoptosis, these processes were examined at different time points in fungal cells grown in liquid cultural by M52 staining (i.e. the fluorescent probe perylene-3,4-dicarboxylic anhydride) and TUNEL assays, respectively, as detailed in the Methods section. Autophagosome staining of fungal cells grown for 14-h in media showed large (single) autophagosomes within each septal compartment of wild type and *MaGlox*-OE hyphae. However, the Δ*MaGlox* exhibited smaller, diffuse, yet still punctuate staining with an overall stronger fluorescence as compared to the wild type (Fig 2F). Similar results were observed at the 20 h growth time point (Fig 2F). TUNEL staining of 14 h cells revealed no differences between the three strains (Fig 2G). However, at 20 h, TUNEL staining was present in only a small fraction of growing hyphae in the wild type and *MaGlox*-OE strains, while extensive staining of nuclei was observed in the hyphae of the Δ*MaGlox* strain (Fig 2H). Additionally, live/dead staining using propidium iodide (PI) showed strong and extensive staining of nuclei in Δ*MaGlox* hyphae, which was not seen in the wild type and *MaGlox*-OE strains (Fig 2H). To determine whether the apparent increase in autophagosomes and subsequent apoptosis correlated with increased expression of autophagy and cell death genes, RT-qPCR analysis of the autophagy-related genes (*atg1*, *atg3*, *atg4*, *atg5*, *atg8*, *atg11*, *atg12*, *atg13*, and *atg17*) and apoptosis-related genes (*Cas*, *CasA1*, *CasA2*) were performed on fungal cells grown in media. Results showed significantly (*P* < 0.01) increased expression of the *atg* genes (*atg4*, *atg8*, *atg11 atg12*, *atg13*, *atg17* and *atg101*) and all the three apoptosis-related genes in the Δ*MaGlox* mutant as compared to the wild type (Fig 2I).

### MaGlox is essential for maintaining intracellular balance of ROS

**Fig 3:**
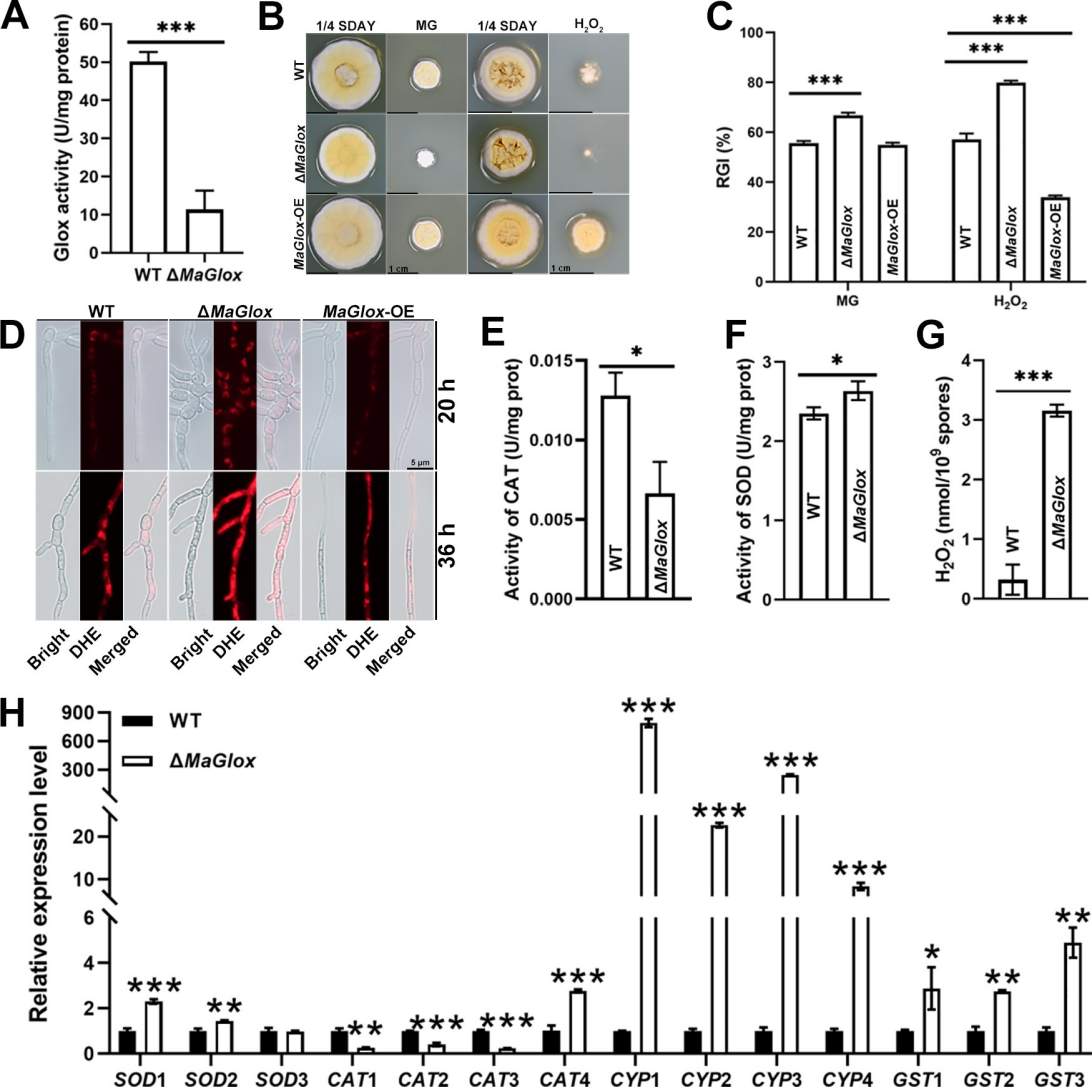
*MaGlox* is essential for maintaining intracellular balance of redox. (A) Glox enzyme activity with MG as substrate in fungal hyphae. Colony morphology (B) and relative growth inhibition (RGI) rate (C) of the WT, Δ*MaGlox* grown for 5 d on ¼-SDAY supplemented with 12 mM H_2_O_2_ and MG with a concentration of 0.71mg/mL. (D) ROS was detected by fluorescence probe DHE at 20 h and 36 h post inoculation in ¼-SAY medium. CAT (E) and SOD (F) enzyme activity in fungal hyphae. (G) Concentration of intracellular H_2_O_2_ of WT and Δ*MaGlox*. (H) Relative expression level of *SOD*, *CAT* and *CYP* genes of WT and Δ*MaGlox* strains. Tukey’s HSD, *: *P* < 0.05; **: *P* < 0.01; ***: *P* < 0.001.

Enzyme activity assays revealed that the Δ*MaGlox* strains exhibited an ~80% reduction in Glox activity compared to the wild type (*P* < 0.001) (Fig 3A). Additionally, tolerance to methylglyoxal and H_2_O_2,_ which are substrates and reaction products of the enzyme, respectively, were significantly decreased for the Δ*MaGlox* mutant (Fig 3B and 3C). However, similar to the wild type, the growth of the *MaGlox*-OE strain was inhibited at high concentrations of methylglyoxal, although *MaGlox*-OE colonies appeared slightly fluffier. In contrast, the *MaGlox*-OE strain demonstrated significantly increased tolerance to H_2_O_2_ compared to the wild type (Fig 3B and 3C). Staining for cellular ROS levels with the peroxide indicator dihydroethidium (DHE) revealed strong fluorescence in the Δ*MaGlox* strain, whereas only a weak signal was observed in the hyphae of the wild type and *MaGlox*-OE strains for cells grown for 20 h, and fluorescence was strongly increased at the 36 h growth time point for all three strains (Fig 3D). Enzyme assays also revealed a significant reduction in total catalase (CAT) activity, coupled with increased superoxide dismutase (SOD) activity in the Δ*MaGlox* strain as compared to the wild type (*P* < 0.01, Fig 3E and 3F). Furthermore, alterations in the expression of genes related to oxidative stress tolerance were observed, including elevated levels of the expression of *SOD* and glutathione-S-transferase (*GST*) genes (*P* < 0.01) in the Δ*MaGlox* strain, while three out of four tested *CAT* genes were significantly downregulated (*P* < 0.01, Fig 3G). Furthermore, intracellular H_2_O_2_ levels increased 10–12 fold in the Δ*MaGlox* mutant as compared to the wild type strain (Fig 3H).

### Loss of *MaGlox* results in accumulation of cellular RCS

**Fig 4:**
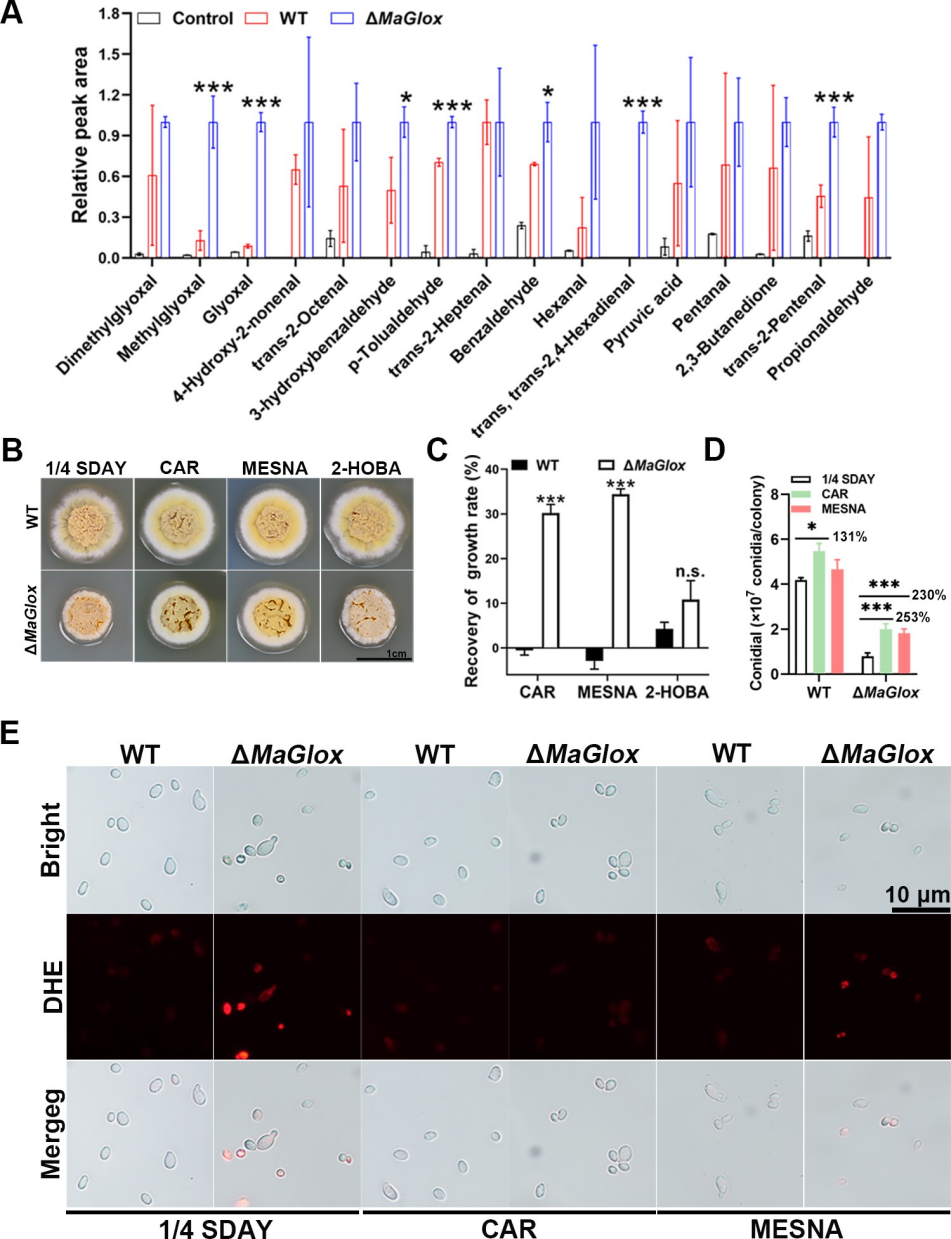
*MaGlox* is involved in RCS accumulation and growth. (A) Intracellular aldehyde content of WT and Δ*MaGlox* cultured in ¼-SDY medium for 48 h. ¼-SDY medium was used as control. Colony morphology (B) and recovery of growth rate (C) of the WT and Δ*MaGlox* strains cultured for 5 d on ¼-SDAY plates supplemented with 400 μg/mL HOBA, 125 μg/mL MESNA and 10 mM CAR, respectively. Conidial yield (D) and ROS staining (E) of wild type and Δ*MaGlox* strains cultured on ¼-SDAY plates and ¼-SDAY supplemented with and 10 mM CAR and 125 μg/mL MESNA, respectively. Tukey’s HSD, n.s.: no significant; *: *P* < 0.05; ***: *P* < 0.001.

The substrates for Glox are small chain aldehydes (*e*.*g*., glyoxal and methylgloxal), which represent an important class of RCS that are often highly toxic to cells and can lead to generation of ROS [30–34]. To explore if loss of *MaGlox* affected intracellular levels of RCS, UHPLC-QTOF-MS was applied for measurement of fungal metabolites as detailed in the Methods section. A total of sixteen RCS species were detected and their concentrations were quantified (Fig 4A and S2 Table). Concentrations of seven RCS compounds: 2,3-butanedione, methylglyoxal, glyoxal, p-tolualdehyde, benzaldehyde, 3-hydroxybenzaldehyde, trans-2-pentenal, and trans, trans-2,4-hexadienal were significantly increased in the Δ*MaGlox* mutant as compared to wild type (*P* < 0.01). Of note among these, concentrations of methylglyoxal and glyoxal, the optimal substrates of Glox [9], were 6.7 and 10.3 times higher in the mutant than that seen for wild type, respectively.

RCS scavengers, which comprise thiol-, amino- and imidazole groups, are capable of scavenging RCS [33]. To determine if the accumulation of RCS play a role in the growth inhibition of *M*. *acridum*, three types of RCS scavengers, 2-hydroxybenzylamine (2-HOBA), carnosine (CAR) and sodium 2-mercaptoethanesulfonate (MESNA), were used to test potential phenotypic rescue on fungal growth. The results showed that CAR (10 mmol/L) and MESNA (125 μg/mL) significantly recovered the colonial growth defects seen in the Δ*MaGlox* mutant compared to wild type. However, exogenous addition of 2-HOBA at 400 μg/mL did not rescue the growth phenotype of the Δ*MaGlox* mutant (Fig 4B and 4C), likely due to the observation that 2-HOBA has a relatively short half-life of 62 minutes, resulting in a relatively poor performance in phenotype recovery [35].

Conidial yield was also determined after RCS scavengers were included in the media. Results showed that CAR and MESNA supplementation in the media resulted in an ~130–150% increase in conidial yield for Δ*MaGlox* as compared to an ~30% increase for the wild type when CAR was added and no changes when MESNA was included (Fig 4D). In addition, cellular ROS levels were significantly decreased in the Δ*MaGlox* mutant when CAR and MESNA were added (*P* < 0.01, Fig 4E). Expression of autophagy-related (*atg1*, *atg3*, *atg4*, *atg8*, *atg11*, *atg12*, *atg13*, *atg17*) and apoptosis-related genes (*Cas*, *CasA1*, *CasA2*) were also significantly decreased when CAR and MESNA were added during growth of the Δ*MaGlox* mutant (S Fig 3A and 3B).

### Contribution of *MaGlox* to fungal stress tolerance

**Fig 5:**
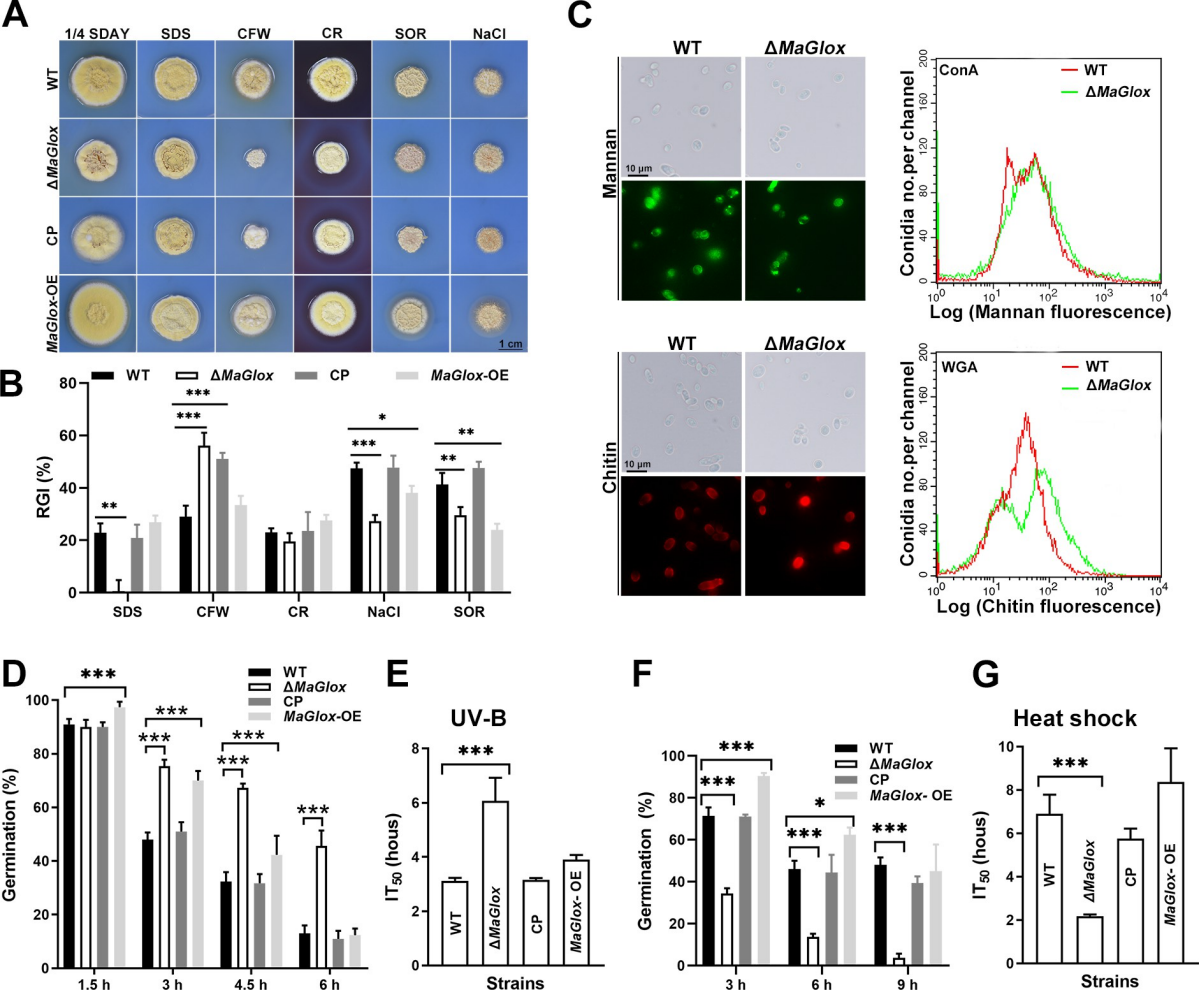
Stress tolerances assays. (A) Colony morphology of the WT, Δ*MaGlox*, CP and *MaGlox*-OE strains grown for 5 d on ¼-SDAY supplemented with 0.01% SDS, 0.05 mg/mL CFW, 0.5 mg/mL CR, 1 M SOR 0.5 M and 0.5 M NaCl, respectively. (B) The RGI rate of each strain on ¼-SDAY with different chemical reagents. (C) Fluorescence intensity for mannan and chitin detection stained with FITC-ConA and FITC-WGA under fluorescence microscopy, respectively. Flow cytometry analysis of the mannan and chitin on fungal cell wall were also performed. (D) Conidial germination after treatment with UV-B. (E) IT_50_ after UV-B treatment. (F) Conidial germination after heat-shock treatment. (G) IT_50_ after heat-shock treatment. Error bars represent standard deviation. Tukey’s HSD, *: *P* < 0.05; **: *P* < 0.01; ***: *P* < 0.001.

In terms of growth in the presence of various cellular stress inducing agents, the Δ*MaGlox* strain was less tolerance to the membrane perturbing agent, calcofluor white (CFW), but showed increased resistance to detergent (SDS), and osmotic stress causing agents, sorbitol (SOR) and NaCl, as compared to the wild type parental strain. The *MaGlox*-OE strain also showed increased resistance to NaCl and SOR (Fig 5A and 5B). Except for the CFW phenotype, the complemented mutant phenotype was restored to wild type. Immunofluorescence and flow cytometry (FCM) were used to measure binding of (fluorescent) lectins to visualize cell surface carbohydrate epitopes (i.e, mannan/glucan and chitin). Results indicated no differences in ConA binding between any of the strains tested (Fig 5C), however, binding of wheat germ agglutinin (WGA, chitin) revealed two apparent populations for the Δ*MaGlox* mutant, with one showing stronger and the other weaker relative fluorescence as compared to controls (Fig 5C). This observation was further confirmed by flow cytometry analyses, in which two close but distinct peaks for Δ*MaGlox* conidia could be discerned (Fig 5C). The Δ*MaGlox* strain also displayed improved tolerance to UV-B radiation with increased germination under these conditions (*P* < 0.01, Fig 5D), and an overall significantly increased length of time of exposure to UV-B required to inhibit germination to 50% of untreated cells (IT_50_, *P* < 0.01, i.e., the mutant showed greater resistance to UV-B exposure of conidia in terms of germination as compared to the wild type and the complemented strains, Fig 5E). However, the Δ*MaGlox* strain had a significantly impaired tolerance to heat shock, showing a 66% decreased IT_50_ compared to the control strain (*P* < 0.01, Fig 5F and 5G). *MaGlox*-OE strain showed no difference in tolerance to UV-B or heat stress as compared to the wild type strain (Fig 5D–5G).

### Loss of *MaGlox* results in decreased virulence

**Fig 6:**
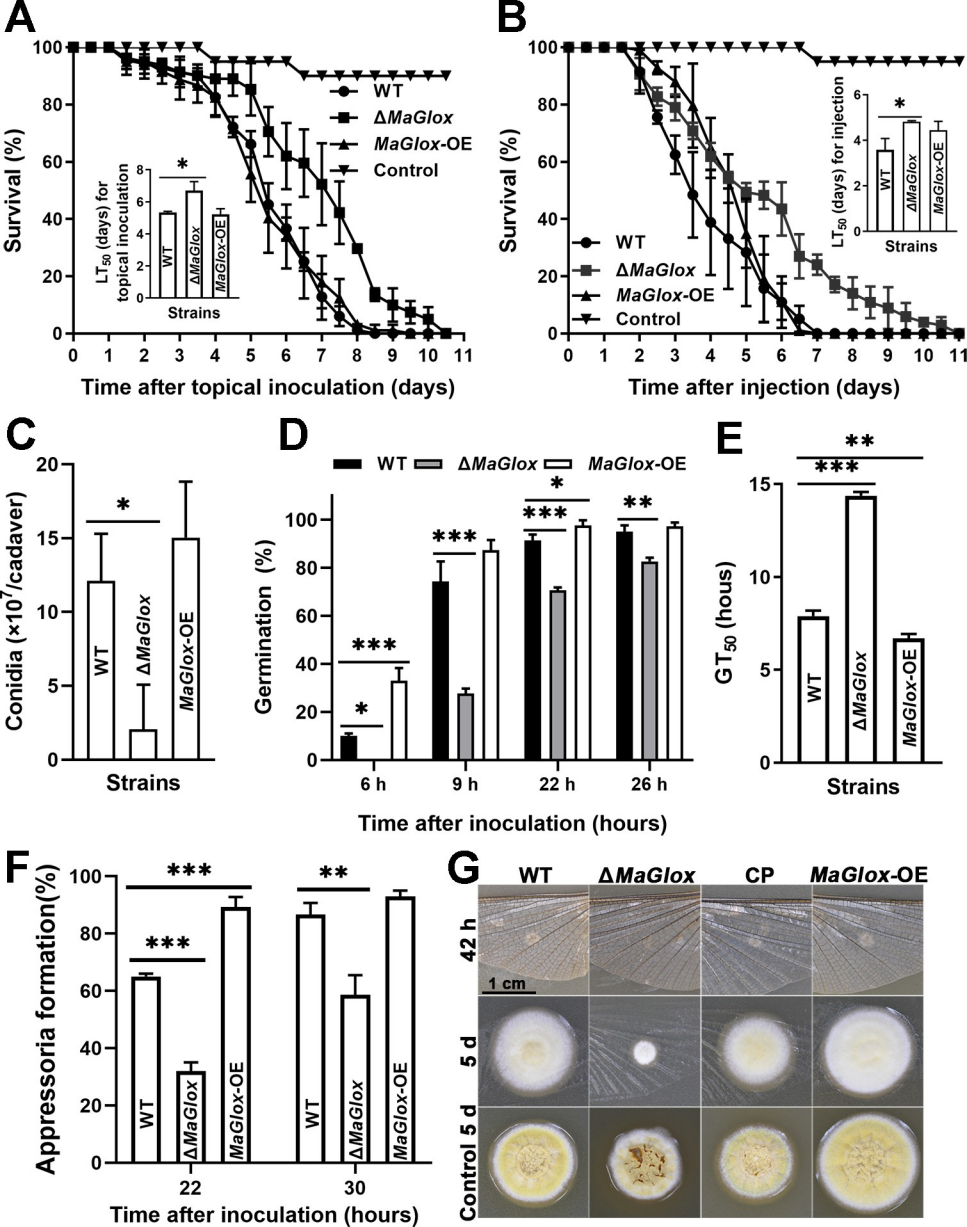
Insect bioassays. (A) Survival of locusts after infected with WT and Δ*MaGlox* strains by topical inoculation and its LT_50_. (B) Survival of locusts after infected with WT and Δ*MaGlox* strains by intra-hemocoel injection and its LT_50_. (C) Conidial yield on locust cadavers 9 d post-death after WT and Δ*MaGlox* strains treatment. (D) Germination rate of WT, Δ*MaGlox* and *MaGlox*-OE strains on locust hind wings at 6, 9, 22 and 26 hpi. (E) The GT_50_ of fungal strains on the locust hind wings. (F) The appressoria formation rate of WT and Δ*MaGlox* strains on the locust hind wings at 22 and 30 hpi. (G) Colony morphology of WT, Δ*MaGlox* and CP strains on the hind wings of locusts. Tukey’s HSD, *: *P* < 0.05; ***: *P* < 0.001.

**Fig 7:**
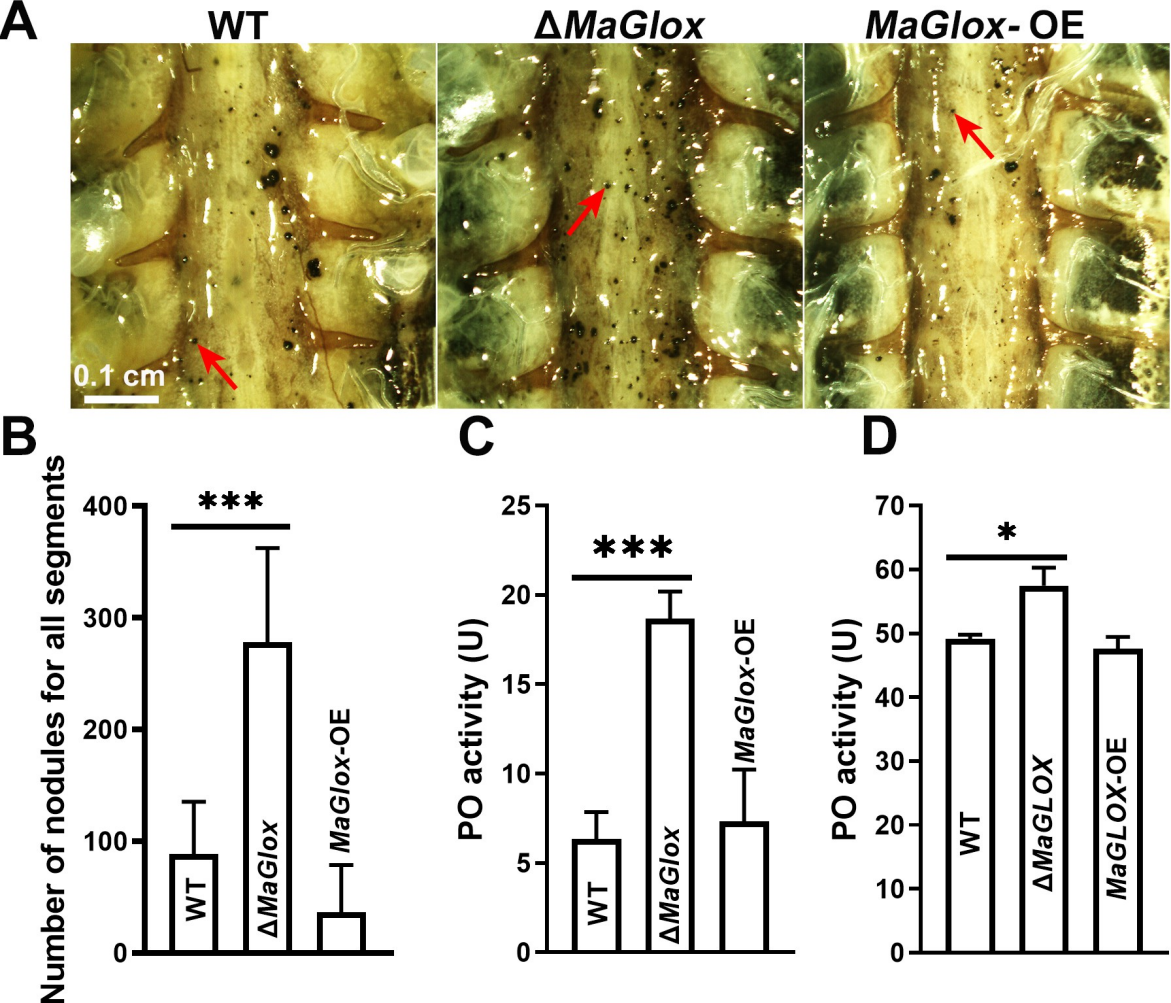
Determination of locust immune response after inoculation with WT and Δ*MaGlox* strains. (A) Nodules (red arrows) in locust inner body walls at 12 h after injection with WT, Δ*MaGlox* and *MaGlox*-OE strains. (B) Nodule number in locusts after injection with WT, Δ*MaGlox* and *MaGlox*-OE strains. (C) PO activity of cell-free hemolymph from locusts at 12 h post topical inoculation. (D) PO activity of cell-free hemolymph from locusts at 12 h post injecton inoculation. Tukey’s HSD, **: *P* < 0.01; ***: *P* < 0.001.

To explore the effects of disruption of *MaGlox* on virulence in *M*. *acridum*: (1) topical inoculation and (2) intrahemocoel injection bioassays were conducted using 5^th^ instar locust nymphs as the host. In topical inoculation, the wild type and *MaGlox*-OE strains killed all locusts within 9.5 d, while similar mortality was seen on day 10.5 for the Δ*MaGlox* treatment group (Fig 7A). Overall, the Δ*MaGlox* group showed a longer calculated time to kill 50% of hosts, LT_50_ = 6.70 ± 0.32 d as compared to wild type, LT_50_ = 5.34 ± 0.04 d and *MaGlox*-OE with 5.22 ± 0.21 d (*P* < 0.01, Fig 6A). Similarly, for the intrahemocoel injection assays, 100% mortality was observed at 7 d for the wild type and *MaGlox*-OE strains, but at 11 d for the Δ*MaGlox* mutant (Fig 6B), with a corresponding change in LT_50_ = 3.58 ± 0.29 d for wild type, 4.44 ± 0.23 d for *MaGlox*-OE and 4.81 ± 0.03 d for the Δ*MaGlox* mutant, respectively, representing a 34% increase, i.e. decreased virulence when disruption of the *MaGlox* (*P* < 0.05, Fig 6B). Sporulation on cadavers was evident for wild type and *MaGlox*-OE infections 9 d post-mortem, however, little outgrowth observed for the Δ*MaGlox* mutant during the same time period (Fig 6C). Quantification of spore production on cadavers indicated 1.21±0.19×10^8^ conidia/cadaver for the wild type, 1.50±0.38×10^8^ conidia/cadaver for *MaGlox*-OE strain, but only 2.05±1.75×10^7^ conidia/cadaver for the Δ*MaGlox* mutant, representing ~83% reduction in Δ*MaGlox* mutant and no difference in *MaGlox*-OE strain compared to wild type (*P* < 0.05, Fig 6C).

To assess whether effects seen with respect to virulence were influenced by germination and/or appressoria (infection structure) formation, these processes were monitored on dissected locust wings. Conidial germination for wild type (10%) and *MaGlox*-OE strain (33%) initiated 6 h post-inoculation onto the wings, whereas little to no germination had yet occurred for the Δ*MaGlox* strain (Fig 6D). At 9 hours post-inoculation, the germination level for the Δ*MaGlox* strain reached 27.7 ± 1.2%, whereas 74.3% of the wild type had already germinated (representing a 62.7% decrease, *P* < 0.01). Germination of *MaGlox*-OE strain showed no difference compared to wild type at 26 h post-inoculation (Fig 6D). Overall, the calculated GT_50_ of the Δ*MaGlox* strain was 14.82 ± 0.16 h, which as significantly increased (*P* < 0.001) compared to the wild type strain (GT_50_ = 7.90 ± 0.17 h, Fig 6E), while the GT_50_ of *MaGlox*-OE strain (6.69 ± 0.14 h) was significantly shorter (*P* < 0.01) than that of wild type (Fig 6E). Appressoria formation was similarly delayed in the Δ*MaGlox* strain (Fig 6F). At 22 h post-inoculation, only ~32% of Δ*MaGlox* had formed appressoria, whereas ~65% of wild type and *MaGlox*-OE strain had, respectively (*P* < 0.001). At 30 h post-inoculation, the appressorium formation by Δ*MaGlox* was ~32% lower than wild type (*P* < 0.01), which had reached ~90%. Appressorium formation of *MaGlox*-OE strain was not significantly different from the wild type (Fig 6F). Cuticle penetration was qualitatively estimated by examining colonies formed through dissected locust wing placed on agar plates after conidia were applied on the wings. These assays showed smaller Δ*MaGlox* colonies as compared to control strains (Fig 6G). The *MaGlox*-OE strain exhibited similar penetration ability to the wild type (Fig 6G).

As the *MaGlox* mutant showed decreased virulence in intrahemocoel injection assays, host immune responses including production of prophenol oxidase (PO) and nodulation were examined (Fig 7). At 12 h post intrahemocoel injection of Δ*MaGlox* conidia into hosts, a two-fold increase in nodule numbers in the ventral diaphragm (to 280 ± 70 nodules/locust) was seen compared to the wild type group (93 ± 30 nodules/locust, *P* < 0.001, Fig 7A and 7B). Similarly, PO activity in locust infected with Δ*MaGlox* was two-fold higher after topical inoculation (*P* < 0.001, Fig 7C), but increased by only 17% in intrahemoceol injection assays (Fig 7D). There were no significant differences between the wild type and *MaGlox*-OE strains in terms of nodule formation or PO activity (Fig 7).

### Transcriptomic analysis

To explore the consequences on global gene expression resulting from loss of *MaGlox*, a comparative transcriptomic analysis was performed between wild type and the Δ*MaGlox* strain grown on ¼-SDAY as detailed in the Methods section (S4A Fig). These data generated 1554 differentially expressed genes (DEGs), of which 669 showed higher expression and 885 showed lower expression in the Δ*MaGlox* mutant as compared to the wild type parent (S4B Fig). The DEGs with per kilobase of transcript per million mapped reads (FPKM) > 10 are listed in S3 Table. Eighteen DEGs associated with growth, stress tolerance and virulence were analyzed by RT-qPCR to verify the transcriptome data. Results showed a high correlation coefficient (R = 0.966) between these two methods, suggesting that the RNA-Seq data are reliably robust (S4C Fig).

**Fig 8:**
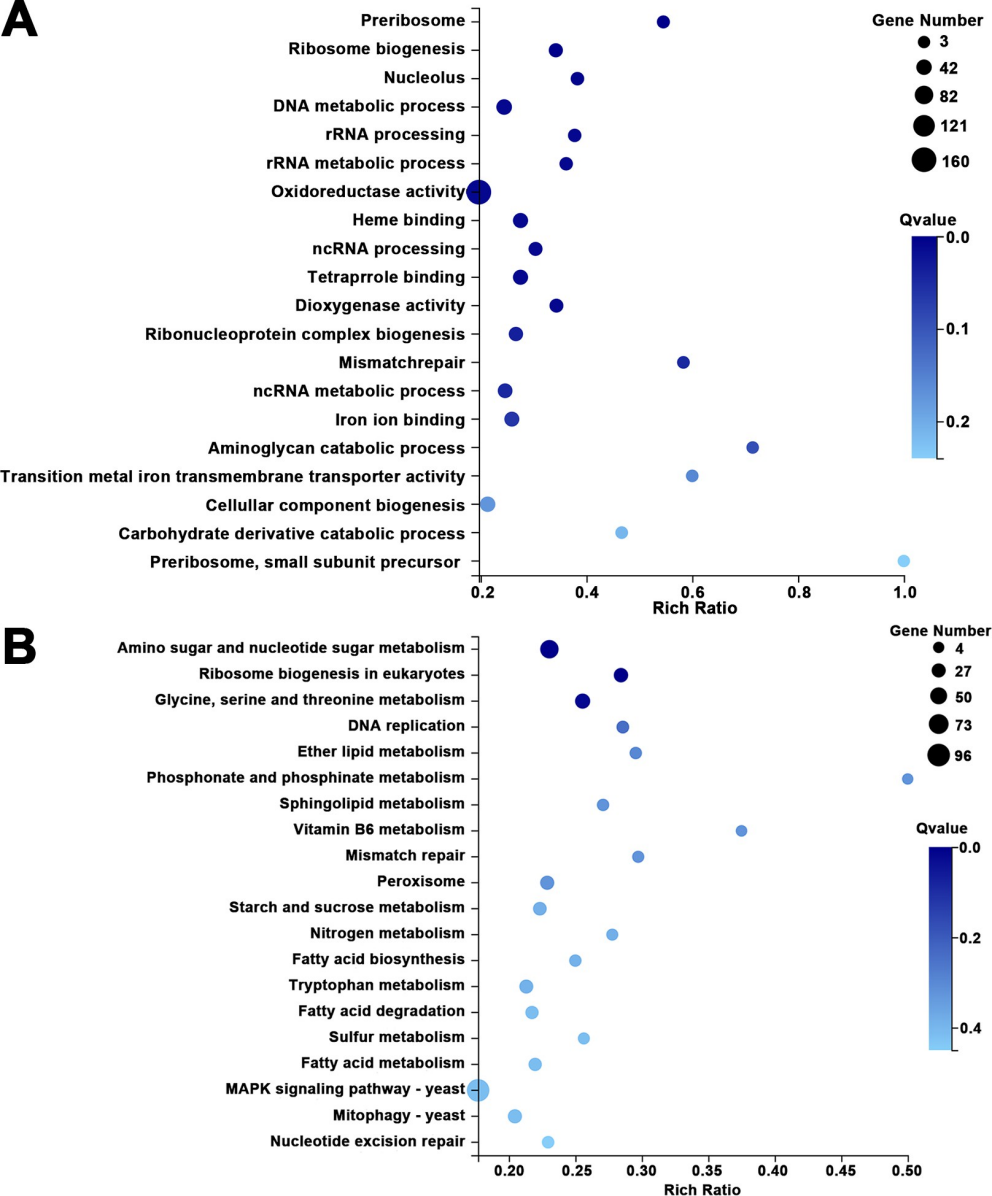
DEGs analysis between Δ*MaGlox* and WT strains. GO enrichment (A) and KEGG pathway enrichment (B) of the DEGs from Δ*MaGlox* vs WT.

DEGs were classified into 36 categories and distributed in molecular function, cell component and biological process (S5A Fig) and were found to be mainly involved in nutrient metabolism, signaling, transporting, growth and cell death (S5B Fig). GO enrichment analyses revealed that the top category of enriched DEGs were associated with oxidoreductase activity (Fig 8A). Many genes involved in scavenging ROS were higher expression in Δ*MaGlox* as compared to wild type, including SOD (MAC_04849, MAC_01993), glutathione S-transferase (MAC_08283, MAC_06995, MAC_07906), cytochrome P450 (MAC_09477, MAC_07120, MAC_05732) and NADPH oxidase (NOX) (MAC_01488) (S3 Table). Expression of glyoxalase I gene (*Gl*o I) (MAC_02360), which directly detoxified MG in a GSH-dependent manner [36], was also up-regulated (S3 Table). Genes responsible for cell wall integrity and remodeling, including glycoside hydrolase (MAC_06492) [37], β-1-3-glucanosyltransferase (MAC_00058) [38], sulfate reductase (NADPH) and flavoprotein component (MAC_03446) [39], showed lower expression in the *MaGlox* mutant as compared to wild type (S3 Table). Genes involved in cell growth and division, such as cell division cycle protein (MAC_02282), meiosis protein MEI2 (MAC_06466), urea active transporter (MAC_06614) and Ran1-like protein kinase (MAC_02716), showed 1–2 fold lower expression in the mutant (S3 Table). The Target of Rapamycin (*TOR*), a central inhibitory regulator of autophagy [40] was downregulated when *MaGlox* was impaired. Other genes downstreamed of TOR in mitophagy pathway were upregulated in Δ*MaGlox* (S6 Fig and S3 Table). Metabolic processes related to ribosome biogenesis, DNA and RNA metabolism were also significantly influenced (Fig 8A). As a carbohydrate-active enzyme (CAZy), the knockout of *MaGlox* disrupted carbohydrate metabolism, resulting in altered expression of numerous associated genes (S3 Table). Additionally, KEGG enrichment analysis indicated that DEGs were significantly enriched in pathways related to amino sugar and nucleotide sugar metabolism, eukaryotic ribosome biogenesis, and glycine, serine, and threonine metabolism (*P* < 0.05) (Fig 8B).

**Fig 9:**
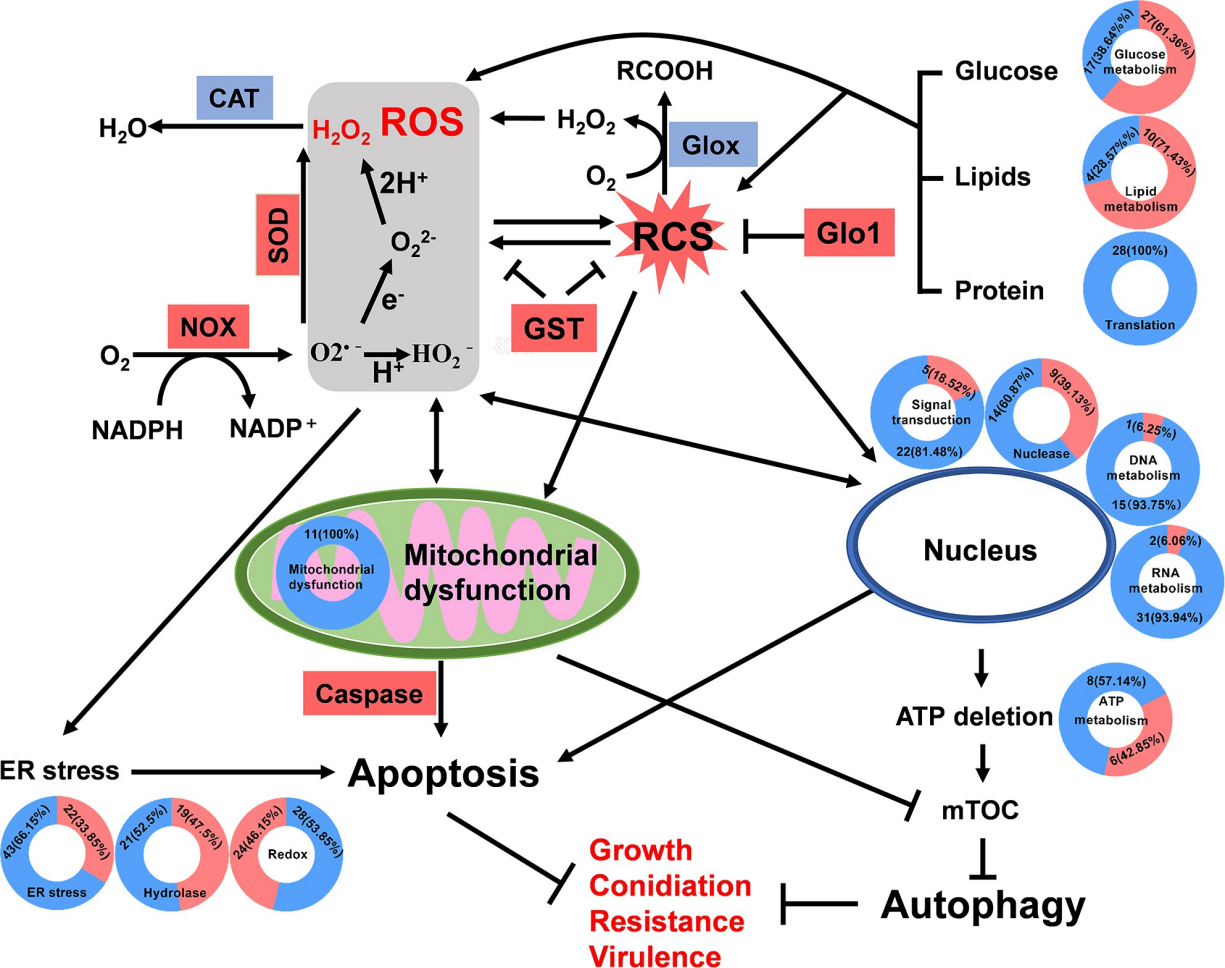
*MaGlox* functions in fungal growth, conidiation, stress resistance and virulence in *M*. *acridum*. MaGlox detoxifies the intracellular RCS to maintain cellular RCS/ROS balance. The loss of *MaGlox* leads to an accumulation of intracellular RCS and ROS, causing the ER stress, mitochondrial dysfunction, and damage to the nucleus, resulted in autophagy and apoptosis. The DEGs encoded NOX and SOD were up-regulation, which were related the generation of ROS. The glyoxalase I (Glo I) directly detoxifying MG were also up-regulated. The red-blue circles represent the DEGs results related the according category. The DEGs included in the red were upregulated and the blue were downregulated, with the number in the circle representing the quantity of DEGs and its proportion in the items. Genes in the rectangle in red indicated up-regulated and blue indicated down-regulated when *MaGlox* was deleted.

To identify virulence-related genes among DEGs (S4 Table), a BLAST analysis was conducted against the pathogen-host interaction (PHI) gene database. There were 270 DEGs involved in virulence, with 38 genes assigned to the hypothetical protein category. Of the remaining 232 genes, 92 were upregulated and 140 downregulated in the mutant as compared to the wild type. Some cuticle degrading enzyme and other factors involved in penetration showed decreased expression, including: aspartyl protease (MAC_01391, MAC_05521) [41], glycosyltransferase (MAC_01796, MAC_01312) [42,43] and glycerol-3-phosphate dehydrogenase (MAC_07043) [44]. Genes showing higher expression levels included two Zn(II)2Cys6 transcription factors (MAC_08402, MAC_09307) contributed to fungal growth and virulence, and a subunit of transcription factor TFIIH (MAC_03555) with a function in virulence and DNA repair [45–48]. A summary model of the functions of *MaGlox* is presented (Fig 9).

## Discussion

Glyoxal oxidases were initially characterized as fungal enzymes in white rot involved in lignin/plant biomass turnover [1]. Additional functions in some phytopathogenic fungi, *e*.*g*., *U*. *maydis* and *F*. *graminearum*, suggest a role in targeting plant tissues (13, 14). However, as these enzymes exist in most fungi, many of whom are not directly involved in lignin degradation, it seems likely that they have broader functions in detoxification of reactive carbonyl species that could be present in the environment and/or generated endogenously as metabolic by products. Our analysis of *Glox* genes shows a clear divide in terms of structural motifs, with the Glox of plant white rot, and mammalian fungal pathogens lacking WSC domains, whereas the genes found in fungal plant and invertebrate pathogens contain from 3–5 such domains. As these domains are known to be involved in binding to chitin/glucans, the functional roles of these two groups of Gloxs are likely to have diverged. Indeed, whereas the white rot Glox is an extracellular enzyme, the *M*. *acridum* Glox is localized to the outer membrane and septa (the latter particularly rich in chitin) likely via the WSC domains.

In terms of function, our data show that the MaGlox contributes to detoxification of intracellular RCS and expression of *MaGlox* is induced by the addition of exogenous aldehyde substrates within a specific concentration range. Although the product of Glox is H_2_O_2_, loss of Glox still resulted in increased cellular H_2_O_2_ levels, likely due to downstream generation as a result of accumulation of toxic RCS, which are markers of oxidative stress [49]. Thus, despite generating H_2_O_2_, in terms of wider cellular metabolism, Glox contributes to decreased levels of both intracellular RCS and ROS, the former via direct enzymatic activity, and the latter as an indirect result, including by affecting (decreasing expression of) catalase activity. ROS are generated through various enzymatic and non-enzymatic processes, including the activities of NADPH oxidases (NOXs), mitochondria, the endoplasmic reticulum, peroxisomes, and external stimuli [50]. Notably, the DEGs involved in maintaining mitochondrial function (11 genes), ribosome biogenesis (28 genes), DNA repair (8 genes), and translation (6 genes) all showed decreased expression in the *MaGlox* mutant (see S3 Table). Additionally, genes associated with endoplasmic reticulum stress, signal transduction, protein/ion transport, and DNA, RNA, and ATP metabolism also showed decreased expression. Decreased expression of these genes likely contributes to the induction of cell death pathways, *e*.*g*., apoptosis, autophagy, and necrosis, that subsequently negatively affects cell growth, conidiation, resistance. In addition, activation of these pathways impairs the ability of the fungus to infect hosts, resulting in decreased virulence. Interestingly, our data indicate that *MaGlox* mutant cells try to compensate for the increased levels of RCS/ROS by increasing expression of potential scavenging enzymes including glyoxalase I, glutathione-S-transferase, superoxide dismutase, and cytochrome P450 genes, however, these are apparently unable to limit the increased levels of toxic RCS/ROS. As the mutant strain grows and metabolizes, the absence of *MaGlox* leads to the accumulation of RCS, which induces autophagy. With the accumulated cellular damage triggering apoptosis. The consequences of the increased RCS/ROS appear to be the induction of a stress responses including autophagosome proliferation, followed by the induction of cellular death pathways. RCS scavengers rescued the impaired growth and conidiation of mutant strain, down-regulated the expression of autophagy and apoptosis-related genes and decreased ROS levels in *MaGlox* mutant strain, supporting the model for MaGlox functioning. The induction of increased autophagy and apoptosis can account for the Δ*MaGlox* phenotypes of reduced vegetative growth, distorted hyphal morphology and elongation, delayed germination, and decreased conidial yield. Some of these phenotypes are consistent with *Glox* mutants of *T*. *virens* and *U*. *maydis*, which also showed impaired hyphal development [13,15], but differ from findings in *Fusarium* spp. [14]. Genes related to fungal growth and development (MAC_03443, MAC_05865, and MAC_03916) were significantly downregulated in Δ*MaGlox*. Of note, expression of elongator complex protein gene (MAC_01832), involved in vegetative growth and conidiation, was reduced by ~75% in the Δ*MaGlox* mutant. Constitutive expression of *MaGlox* promoted germination, colony growth, and increases tolerances to H_2_O_2_ and osmotic stress. This contrasts with the findings in *U*. *maydis*, where *Glox* overexpression does not result in any growth phenotype differences compared to the wild type [15]. This discrepancy may be attributed to the presence of three *Glox* genes in *U*. *maydis*, which exhibit functional redundancy. Nonetheless, the overexpression of *MaGlox* exerts a significantly less pronounced effect on fungal phenotypes compared to its disruption. This reduced impact is likely due to the overexpression of *MaGlox* not substantially altering the overall metabolic flux, as other rate-limiting metabolic steps remain influential, leading to less pronounced phenotypic differences.

Entomopathogenic fungi are natural biological insecticides that have evolved a series of stress tolerance and detoxification systems to mitigate biotic and abiotic stresses including those that act as host (immune) defenses [28]. The Δ*MaGlox* mutant exhibited decreased tolerances to heat-shock, cell wall stressors and oxidative stresses, but little no changes were seen with respect to osmotic stress. Exogenous H_2_O_2_ has been shown to partially restore hyphal growth and conidiation in a *T*. *virens* Δ*Glox* mutant [13], however, this contrasts with our findings in *M*. *acridum* where addition of exogenous H_2_O_2_ or methylglyoxal was toxic to the Δ*MaGlox* strain. In terms of the fungal cell wall (and septa), chitin is a major complex carbohydrate component of these structures [51]. Changes in the distribution and content of chitin in the cell wall of Δ*MaGlox* can affect fungal cell wall integrity, thereby affecting tolerance to cell wall stresses, as well as normal growth and development. With respect to virulence, *MaGlox* affects the formation of infection structures, conidial penetration of the insect cuticle, and proliferation in the host/evasion of host immune responses. Such consequences can be due to either failure to detoxify metabolic by products mobilized during infection, *e*.*g*., lipids during appressoria formation, and/or failure to detoxify exogenous RCS produced by the host either as a defense response or via its own metabolic activities. Expression of *M*. *acridum* chitinase [52,53] and aspartyl protease (MAC_01391) [51], both of which function in hydrolyzing the insect cuticle, were decreased by >80% in the *MaGlox* mutant, thus helping to account for the decreased cuticle penetration phenotype. Intriguingly, expression of a KP4 killer toxin protein homolog (MAC_04104), involved in plant fungal pathogenicity, was decreased by 95% in the Δ*MaGlox* strain, although the consequence of this remains unknown. Impaired virulence was also marked by a decrease in the ability of the mutant to overcome a number of host immune defenses [54,55], resulting in an inability to suppress nodule formation and PO activity, to the extent that the wild type could.

## Conclusion

Here, we characterized the *Glox* gene in the entomopathogenic fungus *M*. *acridum*. Our results demonstrate that MaGlox contributes to directly decreasing toxic levels of RCS and indirectly levels of ROS, contributing to oxidative stress tolerance, autophagy, apoptosis, and virulence. These data provide a mechanism by which Glox affects fungal growth, development, and virulence in pathogenic fungi.

## Material and methods

### Strains and culture conditions

*M*. *acridum* strain CQMa102 (China General Microbiological Culture Collection Center, CGMCC, Accession No. 0877, GCF_000187405.1) was used as the wild type (WT) for all experiments. *M*. *acridum* wild type and engineered fungal strains were cultured on ¼ -strength Sabouraud dextrose agar medium (¼-SDAY; 10 g glucose, 2.5 g peptone, 5 g yeast extract, 18 g agar and 1 L water) for conidiation for 15 days at 28°C. *Escherichia coli* BGT1 (BioGround, Chongqing, China) was used for vector construction and *Agrobacterium tumefaciens* AGL-1 (Dingguo, Beijing, China) was for *M*. *acridum* transformation. Locusts, *Locusta migratoria manilensis*, were reared and maintained in the lab as described previously [56].

### Construction of targeted gene disruption, complementation and EGFP-tagged *MaGlox* overexpression strains

Targeted gene disruption and complementation strains of the *MaGlox* gene were constructed with pK2-PB and pK2-Sur as the backbone vectors, which contain the phosphinothricin resistance (*Bar*) cassette and chlorimuron ethyl resistance gene, *Sur*, respectively [57]. Transformants were screened by colony polymerase chain reaction (PCR), and the correct integration event verified by Southern blotting and loss of gene expression confirmed via reverse transcription quantitative PCR (RT-qPCR). Primers for vector construction, verification and probe preparation are listed in S1 Table. Briefly, 1.0-kb 5′- and 3′-flanking sequences of *MaGlox* were separately amplified from wild type genomic DNA with primer pairs Glox_LF/Glox_LR and Glox_RF/Glox_RR. The upstream PCR products were digested with *Xba*I/*Eco*RI and inserted into the pK2-PB vector with a *Bar* cassette (pK2-PB-LGlox). The downstream amplification products were inserted into the *Spe*I/*Eco*RV-digested pK2-PB-LGlox, and the construct was delivered into wild type by *A*. *tumefaciens* to obtain the *MaGlox*-disruption mutant (Δ*MaGlox*). Transformants were screened on Czapek-dox agar containing 500 μg/mL glufosinate ammonium (Sigma, St. Louis, MO, USA) and the desired insertion confirmed by PCR and Southern blotting with DIG-High Prime DNA Labeling and Detection Starter Kit for the latter (Roche, Basel, Switzerland). To generate the complemented Δ*MaGlox*::*MaGlox* (CP) strain, the full-length *MaGlox* sequence including the promoter region was amplified and cloned into vector pK2-Sur. The CP strain was selected on Czapek-dox agar supplemented with 20 μg/mL chlorimuron ethyl (Sigma, Bellefonte, PA, USA).

To examine the subcellular location of MaGlox, an *MaGlox-EGFP* fusion construct was constructed in which expression of the gene was driven by the glyceraldehyde-3-phosphate dehydrogenase gene promoter (*PgpdM*) (EGFP-*MaGlox*-OE). Briefly, the complete *MaGlox* gene (3762 bp) was amplified with primers OE-F/OE-R and fused with *EGFP*. The integrity of the final construct was confirmed by sequencing, and the construct was transformed into wild type *M*. *acridum* as above. Transformants were confirmed by PCR and RT-qPCR.

### Microscopy

Green fluorescence signals in conidia and mycelia for the *MaGlox*-EGFP strain were observed using a Laser Scanning Confocal Microscope (LSCM) (TCS SP8, Leica, Germany). Briefly, fungal cells cultured on ¼-SDAY were collected and washed twice with sterile H_2_O. Sample were fixed using 4% paraformaldehyde for 20 min. Aliquots (5 μL) were loaded on the slide and then sealed after covered by coverslip, which were then placed under the microscope for observation.

### Phenotypic characterizations

#### Growth, hyphal development and conidiation assays

Aliquots of 50 μL conidial suspensions (1×10^6^ conidia/mL) were evenly spread on ¼-SDAY plates and incubated at 28°C for 20 h for measurement of conidial germination as described previously [58]. Mycelial development at 18 h, 24 h and 36 h was observed on ¼-SDAY plates under microscope. Conidial germination and conidial yield analyses was performed on ¼-SDAY as described previously [59].

#### Stress tolerance assays

To investigate the tolerance changes to exogenous aldehyde in Δ*MaGlox*, and whether the phenotypes of the Δ*MaGlox* strain could be rescued via addition of exogenous H_2_O_2_ and RCS scavengers, ¼-SDAY plates were amended with 1.6 mM methylglyoxal (MG, Meryer, China), 6 mM H_2_O_2_ and three RCS scavengers, 400 μg/mL HOBA(Meryer, China), 125 μg/mLMESNA (Meryer, China) and 10 mM CAR (Meryer, China) [60–62]. Colony growth of wild type and Δ*MaGlox* were compared to control unamended plates after incubation at 28°C for 5 d.

Fungal stress tolerance to various chemical agents was determined on ¼-SDAY amended with 0.01% SDS, 0.5 mg/mL Congo red (CR), 0.5 M NaCl, 0.05 mg/mL calcofluor white (CFW), 1 M sorbitol (SOR) respectively. Colony growth was compared to control unamended plates after 5 d of incubation at 28°C. Conidial tolerance to heat-shock (45°C) and UV-B irradiation (1350 mW/m^2^) was conducted as described previously [63]. The IT_50_ after heat-shock or UV-B exposure was compared between the wild type, Δ*MaGlox* and complemented (CP) strains.

#### Measurement of cell wall components

To analyze the effect of loss of the *MaGlox* gene on cell wall components, fluorescence-labeled antibodies and flow cytometry were used to detect the distribution of chitin and mannan/glucan on the conidial cell wall surface as described previously [64]. Fungal cell wall chitin was stained with fluorescein isothiocyanate (FITC)-wheat germ agglutinin (WGA) (Invitrogen, Carlsbad, CA, USA) and mannan/glucan was stained with FITC-concanavalin A (ConA, Vector Laboratories, Burlingame, CA, USA) according to the operation manual. Fluorescence was analyzed with BD FACSCalibur Flow Cytometer (Becton Dickinson, San Jose, CA, USA) and BD CellQuest Pro and FACS Express v3. Mannan/glucan bound with FITC-ConA was detected at excitation wavelength (Ex) of 488 nm and the emission wavelength (Em) of 530 nm. The chitin was examined at the Ex of 488 nm and Em of 630 nm. The analysis of each experiment was repeated three times. All phenotypic and cell wall experiments contained three technical replicates and the entire experiment repeated three times.

### Glyoxal oxidase activity assays

Glox enzyme activity was assayed according to the protocol previously described with modifications [65]. Culture of wild type and Δ*MaGlox* in ¼-SDY grown for 2 d at 28°C were harvested and ground under liquid nitrogen. The resulting tissue powder was suspended in 1 mL of 10 mM PBS (pH 7.0). After centrifugation with 5000 rpm at 4°C for 10 min, the supernatant was filtered using a 30-kDa ultra centrifugal filter (6000 rpm, 15 min, 4°C). Proteins retained on the filter membrane were dissolved in 1 mL 50 mM sodium citrate buffer (pH 6.0) containing 5 mM EDTA. Glox enzyme activity was determined by measuring H_2_O_2_ formation using a coupled assay with horseradish peroxidase (HRP, Sigma-Aldrich, China) and 2,2′-azino-bis (3-ethylben zothiazoline-6-sulfonic acid) (ABTS, Solarbio, China) according to the manufacturer’s instructions. The reaction mixture contained 50 mM sodium citrate buffer (pH 6.0) containing 1 mM ABTS, 7 U HRP, 200 μg protein sample and 10 mM substrate methylglyoxal in a total reaction volume 1 mL. The reaction was initiated by the addition of methylglyoxal and the lag phase was eliminated by the addition of 5 μM H_2_O_2_ as described previously [66]. The absorbance of oxidized ABTS was measured at 420 nm for 3 min at 30°C using a SPECTRAmax 190 Spectrometer (MDC, USA). One enzyme unit was defined as the amount of enzyme that converts 1 μmol of O_2_ to H_2_O_2_ per minute under the assay conditions. Assays were conducted with three technical replicates and using triplicate biological samples.

### Insect bioassays

Insect bioassays were performed using fifth instar *L*. *migratoria* nymphs via (1) topical inoculation and (2) intrahemoceol injection as described previously [67]. For topical bioassays, aliquots of 5 μL conidial suspensions (1×10^7^ conidia/mL) prepared in paraffin oil and conidia were inoculated onto the pronotum of the locust. For intrahemoceol injection assays, aliquots of 5 μL conidial suspensions (1×10^6^ conidia/mL H_2_O) were injected into the hemocoel using a microinjector. Respective treatments with paraffin oil or sterile ddH_2_O were used as the blank control for topical bioassay and intrahemocoel injection assays. For each bioassay, three groups of 30 locusts were treated for each test fungal strain and the experiment repeated three times. The number of dead locusts was recorded daily over a period of 11 days. LT_50_ was estimated and compared between the wild type and Δ*MaGlox* strains.

To quantify the total number of spores on the surface and inside of the locusts, host cadavers were collected at 9 d after death, immersed in liquid nitrogen and then ground into a powder and suspended in 2 mL 1% Tween-80. The number of conidia was quantified using a hemocytometer. The experiments were performed in triplicate with three biological samples.

### Germination, appressorium formation and penetration assay on locust wings

Conidial germination and appressoria formation on locust hind wings were determined as described previously [68]. Briefly, ten sterilized locust hind wings were vortexed in 5-ml tube containing 4 ml conidial suspensions in 0.05% Tween-80 (1×10^7^ conidia/mL) for 10 min and then spread on the glass slide gently. The glass slides were laid in petri dishes at 28°C with wet filter paper to keep humidity. Conidial germination was quantified at 6 h, 9 h, 22 h and 26 h and appressorium formation was observed at 22 h and 30 h. Conidia were considered germinated when the germ tube was greater than or equal to the width of the conidia. Appressoria were scored visually. For examining fungal penetration of insect wings, sterilized wings were laid on ¼-SDAY plates. Aliquots of 2 μL spore suspension (1×10^7^ conidia/mL) were inoculated in the middle of wings. After incubation for 42 h, the wings were carefully removed, and the plates were continuously incubated for an additional 3 days. Plates in which the spore suspension was directly spotted on ¼-SDAY and grown for 5 d were used as control.

### Measurement of host innate immune responses to fungal infection

For examination of locust nodule formation, 5 μL conidial suspension (1×10^8^ conidia/mL ddH_2_O) indicated fungal strains was injected into fifth instar locust nymphs. At 12 h post injection, a mid-dorsal cut was made along the full length of the body. The gut and fat bodies were removed to expose the inner ventral surface and then the number nodules were counted in all abdominal segments under a dissecting microscope as previously described [64]. Each experiment examined 10 individuals and all experiments were repeated three times.

Prophenol oxidase (PO) activity in hemolymph was tested using L-dopamine (Aladdin, Shanghai, China) as the substrate [69]. Briefly, 5 μL conidial suspension (1×10^6^ conidia/mL H_2_O) was injected into insect hemolymph, and hemolymph was collected from ten fifth-instar locust nymphs at 12 h post inoculation. The hemolymph sample was centrifuged at 500 g at 4°C and the cell free supernatant collected. PO reaction mixtures (200 μL) containing 20 μL cell free hemolymph, 50 mM PBS (pH 6.5), 150 mM NaCl, and 10 mM L-dopamine were incubated at 28°C for 28 h after which the absorbance was recorded at 470 nm with a TriStar LB941 multifunctional microplate reader (Berthold, Bad Wildbad, Germany). The trial was performed in triplicate with 10 locusts/experiment. Protein concentration in cell free hemolymph was determined by the bicinchoninic acid method using a BCA Protein Assay Kit (CWBIO, Beijing, China). One unit (U) of PO was defined as the amount of enzyme that increases 0.001 of absorbance at A470 per minute per mg protein.

### Autophagy and apoptosis assays

Autophagy was analyzed using the M52 probe kit (Bestbio, Shanghai, China) of autophagosomes according to the manufacture’s protocols, which contains fluorescent probe perylene-3,4-dicarboxylic anhydride to detect mitophagy without mitochondria damage [70]. Briefly, the samples washed with PBS was incubated at 37°C with M52 diluted with detection buffer for 15 min. Fluorescence was observed via microscopy (Nikon Y-TV55, Tokyo, Japan) at an excitation wavelength of 460 nm. Apoptosis was assessed using the terminal deoxynucleotidyl transferase-mediated dUTP nick end labelling (TUNEL) assay, as previously described [71] with a detection kit (Beyotime, China). Briefly, hyphae were washed with 10 mM PBS and fixed in 4% paraformaldehyde for 1 hour at 4°C. Samples were then treated with 2 ml of 1 M sorbitol containing Snailase (5 mg/mL; Dingguo, China), cellulase (5 mg/mL; Dingguo, China), and lysing enzyme (5 mg/mL; Sigma-Aldrich, USA) and shaken at 180 rpm for 3 hours at 28°C. The subsequent staining procedures were carried out following the manufacturer’s protocol. TUNEL-positive cells were stained by 3′,3′ diaminobenzidine (DAB). Propidium iodide (PI; Bestbio, Shanghai, China) was used for live/dead cell staining to detect cell apoptosis. PI fluorescence intensities were visualized using a fluorescence microscope at excitation wavelengths of 488 nm. Gene expression levels of a set of autophagy related genes including *atg1*, *atg8*, *atg9*, *atg13*, *atg17* genes and apoptosis-related genes (*Cas*, *CasA1*, *CasA2*) were determined at different fungal developmental stages by RT-qPCR. Primers used for these experiments are given in S1 Table.

### ROS assays

Intracellular ROS levels were detected by using DHE (Uelandy, China) according to the manufacture’s protocols. Briefly, fungal cells were treated with 5 μM DHE in 10 mM PBS (pH 7.4) for 1 hour at 37°C, where DHE reacts with ROS in the cell to form ethidium oxide, which incorporates into chromosomal DNA to produce red fluorescence [72]. Red fluorescent signals were observed under a fluorescence microscope (Nikon Y-TV55, Tokyo, Japan) at 512 nm. For enzyme assay measurements, fungal cells were grown in ¼-SDY for 3 d before harvesting. SOD and CAT activities were measured in the extracts of fungal mycelia that had been collected by filtration, washed, and ground in liquid nitrogen, following the guidelines provided by the manufacturer using commercial kits available for each enzyme (BC0200, BC0170, Solarbio, Beijing, China). Intracellular H_2_O_2_ levels in conidia were quantified utilizing a hydrogen peroxide test kit according to the manufacturer’s protocol (BC3595, Solarbio, Beijing, China). The transcription levels of cytochrome P450 (*CYP60*, *CYP64*, *CYP105*, *CYP_*fungal), *SOD* and *CAT* genes were determined via RT-qPCR. Primers used in these experiments are given in S1 Table.

### RCS determination

RCS were determined using UHPLC-QTOF-MS [30], with the raw data analyzed using MassHunter Workstation software (Agilent Technologies, version10.1). Fungal strains were grown in ¼-SDY for 2 days before hyphae were harvested by centrifugation (8000 g, 5 min), washed with ddH_2_O. Hyphal samples were ground into a fine powder in liquid nitrogen and 50 mg samples were dissolved in 100 μL ice-cold perchloric acid (1 M) and 400 μL ddH_2_O. After vortexing for 2 min, the samples were centrifuged for 15 min at 4°C, and a 100 μL aliquot of the supernatant was mixed with 20 μL of 25 mg/mL butylated hydroxytoluene (Solarbio, China) and 20 μL of 1 M perchloric acid. Samples were then vortexed for 2 min and centrifuged at 14000 rpm for 15 min at 4°C. Supernatants (80 μL) were derivatized with 200 μL of 200 mM precleaned 2,4-dinitrophenylhydrazine (DNPH) (Shanghai Macklin Biochemical Technology, China) in the dark at 37°C with shaking for 2 h. The precleaned DNPH was prepared by dissolving the reagent in 16 ml solution containing 12 mol/L HCl, ddH_2_O and acetonitrile (Honeywell, South Korea) in the ratio 2:5:1 (v/v/v). After extraction with hexane twice for derivatization, the samples were centrifuged at 14000 rpm for 10 min at 4°C and the supernatant was used for further analysis. Acetonitrile was used as the control. Experiments were performed with three biological samples.

Chromatographic separation was performed using an Agilent 1290 Infinity II series UHPLC system (Agilent Technologies, Germany) equipped with a C_18_ column (150 mm × 2.1 mm, 1.9 μm, Thermo Fisher Scientific, USA). Mobile phase (A) was 0.1% formic acid-water and phase (B) was 0.1% formic acid-acetonitrile. The gradient elution condition was set as: 0–5 min, 2% B; 5–20 min, 2%-80% B; 20–25 min, 80%-100% B; 25.0–28.0 min, 100% B; 28–29 min, 100%-2% B; 29–30 min, 2% B. The flow rate was 400 μL/min and the injection volume was 10 μL. The column temperature was maintained at 30°C.

High resolution mass spectrometry (HRMS) analyses were carried out using a 6546 quadrupole time-of-flight MS (QTOF-MS; Agilent, UK) coupled with a Dual AJS ESI source operating in negative mode. Source ionization parameters were as follows: gas temperature, 320°C; gas flow, 8 L/min; nebulizer, 35 psi; sheath gas temperature, 350°C; sheath gas flow, 11 L/min; capillary voltage, 3500 V; nozzle voltage, 1000V; capillary outlet voltage, 150 V; Fixed collision energies, 10V, 30V and 50V. The MS spectra were acquired in a mass range from 50 to 500 *m/z*. In the MS-MS mode, the TOF analyzed ions from 50 to 500 *m/z*. The MS and MS-MS scan rate were 1 Hz and 2 Hz, respectively; and reference mass, *m/z* 119.0363, 1033.9881.

### Gene expression profiling

Total RNA was extracted from 15 d conidia harvested from ¼-SDAY growing at 28°C using the Ultrapure RNA Kit (CWBIO, Beijing, China) according to the manufacturer’s instructions. Three biological replicate samples for each strain were used for sequence analyses. The first and second cDNA strands were prepared as described previously [57]. The cDNA libraries were sequenced on DNBSEQ 500 platform (BGI, Shenzhen, China) and the raw data were filtered using SOAPnuke (BGI, Shenzhen, China). After clean reads were aligned to the reference genome (GCF_000187405.1). The genes with transcription level change at least 2-fold with a Q-value ≤ 0.001 were defined as differentially expressed genes (DEGs). Annotation and classification of DEGs were performed using Gene Ontology (GO) and KEGG pathway analysis. The DEGs were searched against the PHI-base database (http://www.phi-base.org/) using DIAMOND to identify pathogenic-related genes.

### RT-qPCR

Total RNA was reversed transcribed into cDNA using PrimeScript RT reagent Kit with gDNA Eraser (TaKaRa, Dalian, China). Gene expression levels of specific genes were determined using TB Green Premix Ex Taq II according to the manufacture’s protocols. The target gene transcript level was calculated using the 2^-ΔΔCt^ method [73]. Quantification of transcripts was normalized to expression levels of the glyceraldehyde dehydrogenase (*Magpd*, EFY84384) gene. Three (technical) replicates for each sample were included, and the experiment was repeated three times (biological replicates). Primers for RT-qPCR are listed in S1 Table.

### Statistics analyses

GraphPad Prism 7.0 and SPSS 22.0 software (SPSS Inc., Chicago, IL, USA) were used for statistics analysis. One-way ANOVA analysis followed by Tukey’s post hoc HSD tests was performed to separate means at *P* < 0.05, 0.01 or 0.001. The numerical data used in all figures are included in S1 Data.

## Supporting information

S1 Data Excel spreadsheet containing the underlying numerical data in separate sheets for Figure panels Fig 1B, 1C, 2D, 2E, 2I, 3A, 3C, 3E, 3F, 3G, 3H, 4A, 4C, 4D, 5B, 5D, 5E, 5F, 5G, 6A, 6B, 6C, 6D, 6E, 6F, 7B, 7C, 7D, S2F, S3A, S3B and S4C. (XLSX)

S1 Fig Bioinformatics analysis of MaGlox. (A) Protein structure of the MaGlox amino acid sequence. i: inside; o: outside. (B) Phylogeny analysis of MaGlox. The neighbor-joining tree is constructed by MEGA7. MUSCLE is used for sequence alignment and Gblocks v 0.91b^8^ was for eliminating the regions that couldn’t be unambiguously aligned. All the amino acid sequences used in the phylogenetic tree are obtained from NCBI. Phylogenetic tree is made up of seven entomopathogenic fungi (*M*. *acridum* CQMa 102, *Metarhizium anisopliae*, *Metarhizium robertsii*, *Beauveria bassiana*, *Ophiocordyceps sinensis*, *Purpureocillium lilacinum* and *Pochonia chlamydosporia* 170); two white-rot fungi (*Phanerodontia chrysosporium* and Trametes cinnabarina); six plants (*Jatropha curca*s, *Populus alba*, *Theobroma cacao*, *Vitis pseudoreticulata*, *Nicotiana tabacum*, *Arabidopsis thaliana*); Five phytopathogenic fungi (*Fusarium graminearum*, *Fusarium verticillioides*, *Fusarium oxysporum*, *Neurospora crassa*, *Pyricularia oryzae* 70–15); four human pathogenic fungi (*Histoplasma capsulatum* G186AR, *Talaromyces marneffei* ATCC 18224, *Cryptococcus neoformans var*. *grubii* and *Cryptococcus gattii* CA1280); two *Trichoderma* spp. (*Trichoderma reesei* and *Trichoderma virens*) and *Thermothelomyces thermophilus*. *M*. *acridum* CQMa 102 was shown with red asterisk. (C) Conserved amino acid active site of Glox.
(TIF)

S2 Fig The disruption, complement and overexpression of *MaGlox* gene in *M*. *acridum*. (A) A construction sketch map for *MaGlox* disruption mutant, complementation and overexpression strain. Replacement plasmid pK2-5′-Bar-3′ and pK2-Sur-CP was used for gene disruption and complementation by homologous recombination, respectively. The left border was inserted into the pK2-PB vector with a Bar cassette digested with *Xba*I/*Eco*RI and the right border was inserted into the *Spe*I/*Eco*RV-digested pK2-PB with 5′- flanking sequences of *MaGlox* with *Hin*dIII/*Xba*I and *Eco*RV/*Eco*RI were used to digested Pk2-Sur-3HA. Probe was located at upstream of the left border. (B) Southern blot. About 5 μg of genomic DNA from wild type, and two Δ*MaGlox* transformants named 28# and 29# were digested with *Dra*I and *Pst*I. A 358-bp Probe was amplified with MaGlox_PF/MaGlox_PR. Probe labeling, membrane hybridization, and visualization were performed using the Digoxigenin High-Prime DNA Labeling and Detection Starter Kit I (Roche, Mannheim, Germany). (C) Verification of Δ*MaGlox* transformants with primer pairs Glox-VF/Pt-R and Glox-VR/Bar-F.(D) Verification of OE-*MaGlox* transformants with primer pairs OE-F/EGFP-VR. (E) Verification of CP transformants with primer pairs SurVL-R/CP-VF(L1) and SurVR-F/Glox-RR(L2). (F) Relative expression levels of *MaGlox* gene in 15 d- conidia on ¼-SDAY by RT-qPCR. Error bars represent the standard deviation. (Tukey’s HSD, **: *P* < 0.01; ***: *P* < 0.001).
(TIF)

S3 Fig The Influence of RCS scavengers on the transcription of autophagy and apoptosis-related genes in the Δ*MaGlox* strain. RT-qPCR analysis of the *Cas* and *atg* genes was conducted on the Δ*MaGlox* strains cultured on ¼-SDAY for 7 days, with or without the addition of CAR (A) and MESNA (B).
(TIF)

S4 Fig RNA_Seq analysis. (A) Samples of wild type and Δ*MaGlox* on ¼-SDAY for RNA_Seq. (B) Number of DEGs in Δ*MaGlox* compared to WT. (C) Verification of RNA_Seq results by RT-qPCR.
(TIF)

S5 Fig GO and KEGG pathway classification analysis. (A) GO classification of the DEGs from Δ*MaGlox* VS WT. (B) KEGG pathway classification of the DEGs from Δ*MaGlox* vs WT.
(TIF)

S6 Fig DEGs related to mitophagy in TOR pathway. Green indicates downregulated DEGs and red indicates upregulated DEGs.
(TIF)

S1 Table Primers for vector construction, verification and RT-qPCR. (DOCX)

S2 Table Summary of aldehyde metabolites in *M*. *acridum*. (DOCX)

S3 Table Differentially expressed genes (DEGs). (DOCX)

S4 Table DEGs related to virulence queried against the PHI. (DOCX)
