# Supplementary figures and images for: Glyoxal oxidase-mediated detoxification of reactive carbonyl species contributes to virulence, stress tolerance, and development in a pathogenic fungus
Source: PLoS Pathog. 2024 Jul 30;20(7):e1012431. doi: 10.1371/journal.ppat.1012431 (PMC11315307; doi:10.1371/journal.ppat.1012431)

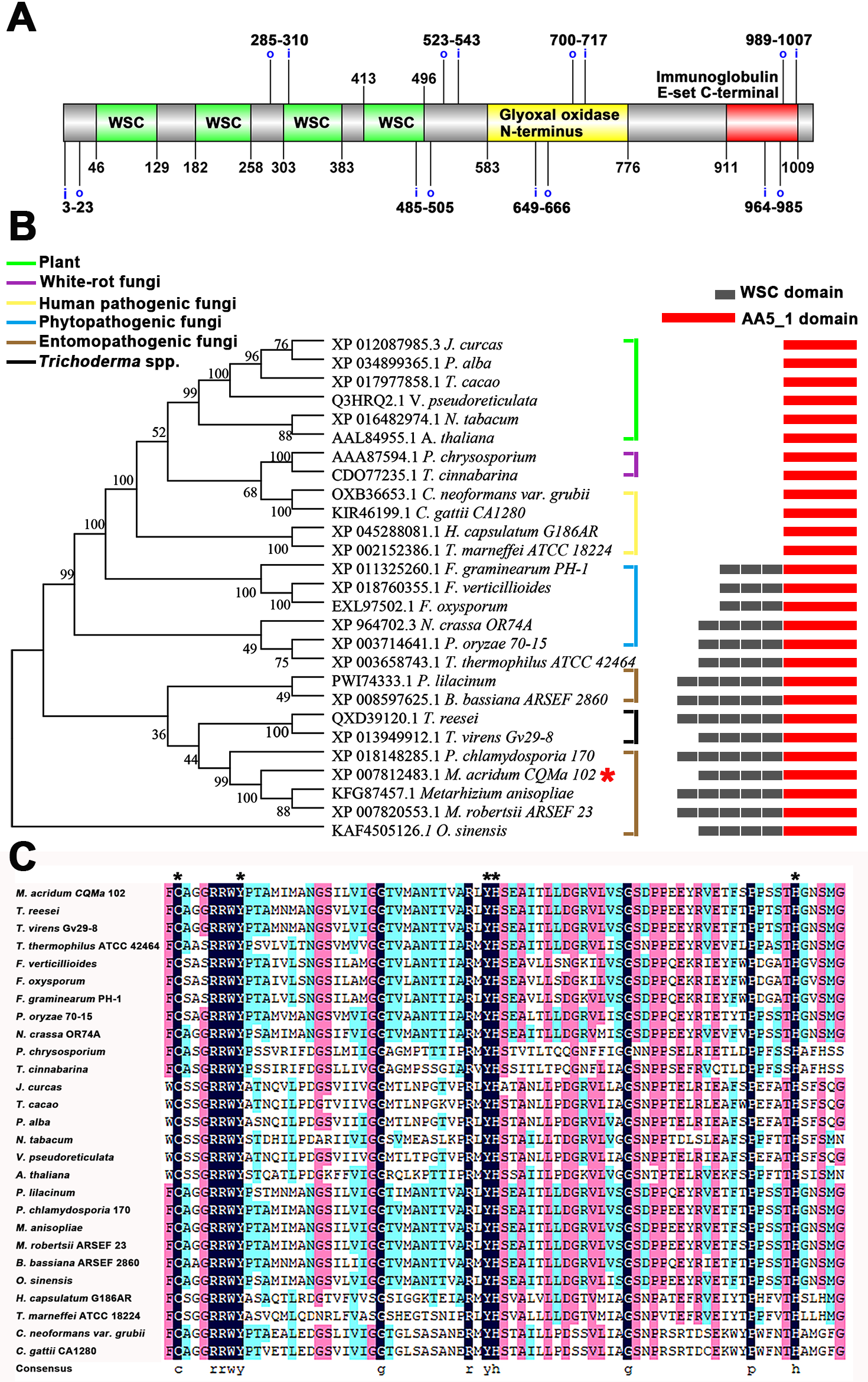

Supplement: S1 Fig — (A) Protein structure of the MaGlox amino acid sequence. i: inside; o: outside. (B) Phylogeny analysis of MaGlox. The neighbor-joining tree is constructed by MEGA7. MUSCLE is used for sequence alignment and Gblocks v 0.91b8 was for eliminating the regions that couldn’t be unambiguously aligned. All the amino acid sequences used in the phylogenetic tree are obtained from NCBI. Phylogenetic tree is made up of seven entomopathogenic fungi (M. acridum CQMa 102, Metarhizium anisopliae, Metarhizium robertsii, Beauveria bassiana, Ophiocordyceps sinensis, Purpureocillium lilacinum and Pochonia chlamydosporia 170); two white-rot fungi (Phanerodontia chrysosporium and Trametes cinnabarina); six plants (Jatropha curcas, Populus alba, Theobroma cacao, Vitis pseudoreticulata, Nicotiana tabacum, Arabidopsis thaliana); Five phytopathogenic fungi (Fusarium graminearum, Fusarium verticillioides, Fusarium oxysporum, Neurospora crassa, Pyricularia oryzae 70–15); four human pathogenic fungi (Histoplasma capsulatum G186AR, Talaromyces marneffei ATCC 18224, Cryptococcus neoformans var. grubii and Cryptococcus gattii CA1280); two Trichoderma spp. (Trichoderma reesei and Trichoderma virens) and Thermothelomyces thermophilus. M. acridum CQMa 102 was shown with red asterisk. (C) Conserved amino acid active site of Glox. (TIF) [file ppat.1012431.s002.tif]

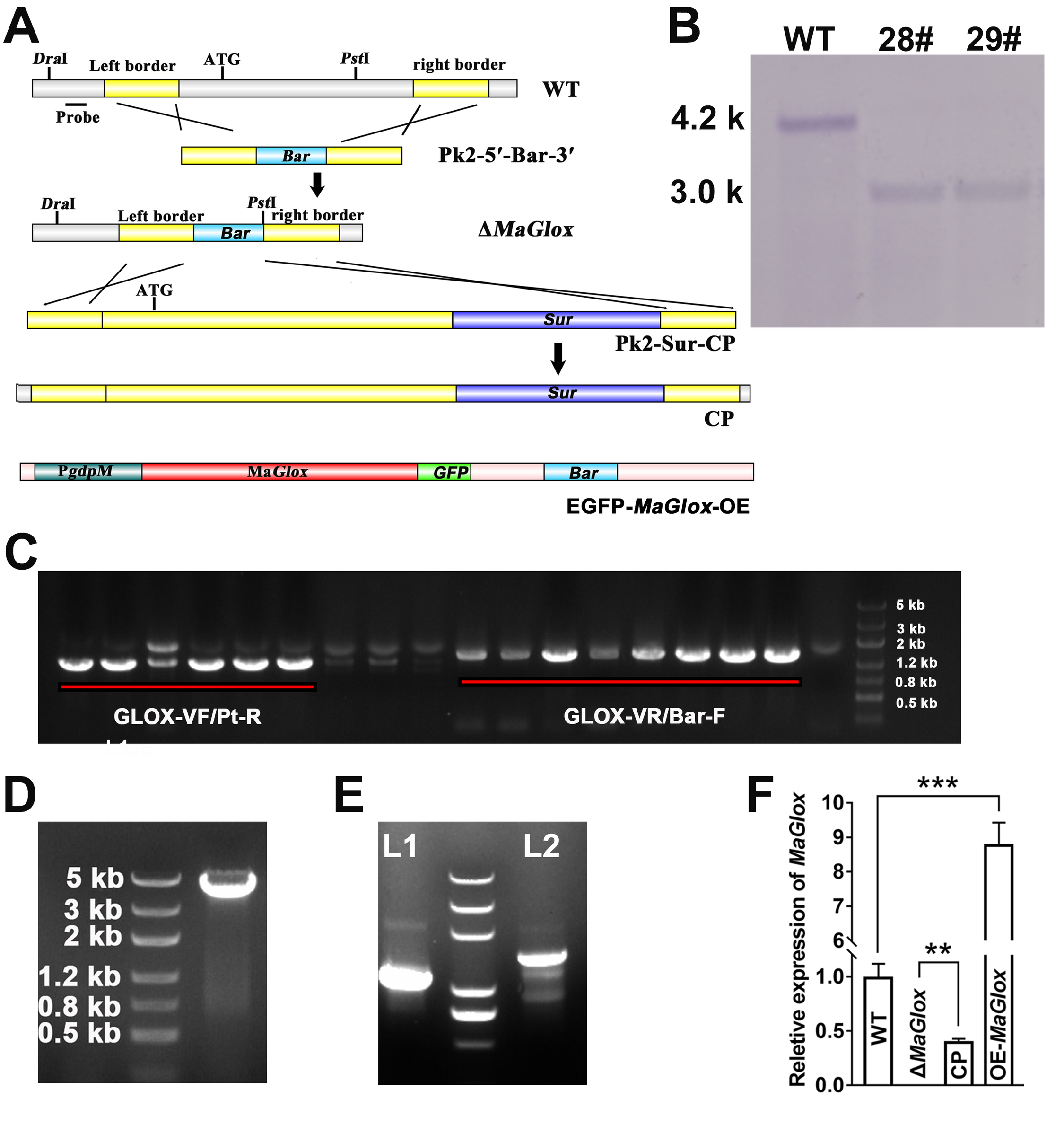

Supplement: S2 Fig — (A) A construction sketch map for MaGlox disruption mutant, complementation and overexpression strain. Replacement plasmid pK2-5′-Bar-3′ and pK2-Sur-CP was used for gene disruption and complementation by homologous recombination, respectively. The left border was inserted into the pK2-PB vector with a Bar cassette digested with XbaI/EcoRI and the right border was inserted into the SpeI/EcoRV-digested pK2-PB with 5′- flanking sequences of MaGlox with HindIII/XbaI and EcoRV/EcoRI were used to digested Pk2-Sur-3HA. Probe was located at upstream of the left border. (B) Southern blot. About 5 μg of genomic DNA from wild type, and two ΔMaGlox transformants named 28# and 29# were digested with DraI and PstI. A 358-bp Probe was amplified with MaGlox_PF/MaGlox_PR. Probe labeling, membrane hybridization, and visualization were performed using the Digoxigenin High-Prime DNA Labeling and Detection Starter Kit I (Roche, Mannheim, Germany). (C) Verification of ΔMaGlox transformants with primer pairs Glox-VF/Pt-R and Glox-VR/Bar-F.(D) Verification of OE-MaGlox transformants with primer pairs OE-F/EGFP-VR. (E) Verification of CP transformants with primer pairs SurVL-R/CP-VF(L1) and SurVR-F/Glox-RR(L2). (F) Relative expression levels of MaGlox gene in 15 d- conidia on ¼-SDAY by RT-qPCR. Error bars represent the standard deviation. (Tukey’s HSD, **: P < 0.01; ***: P < 0.001). (TIF) [file ppat.1012431.s003.tif]

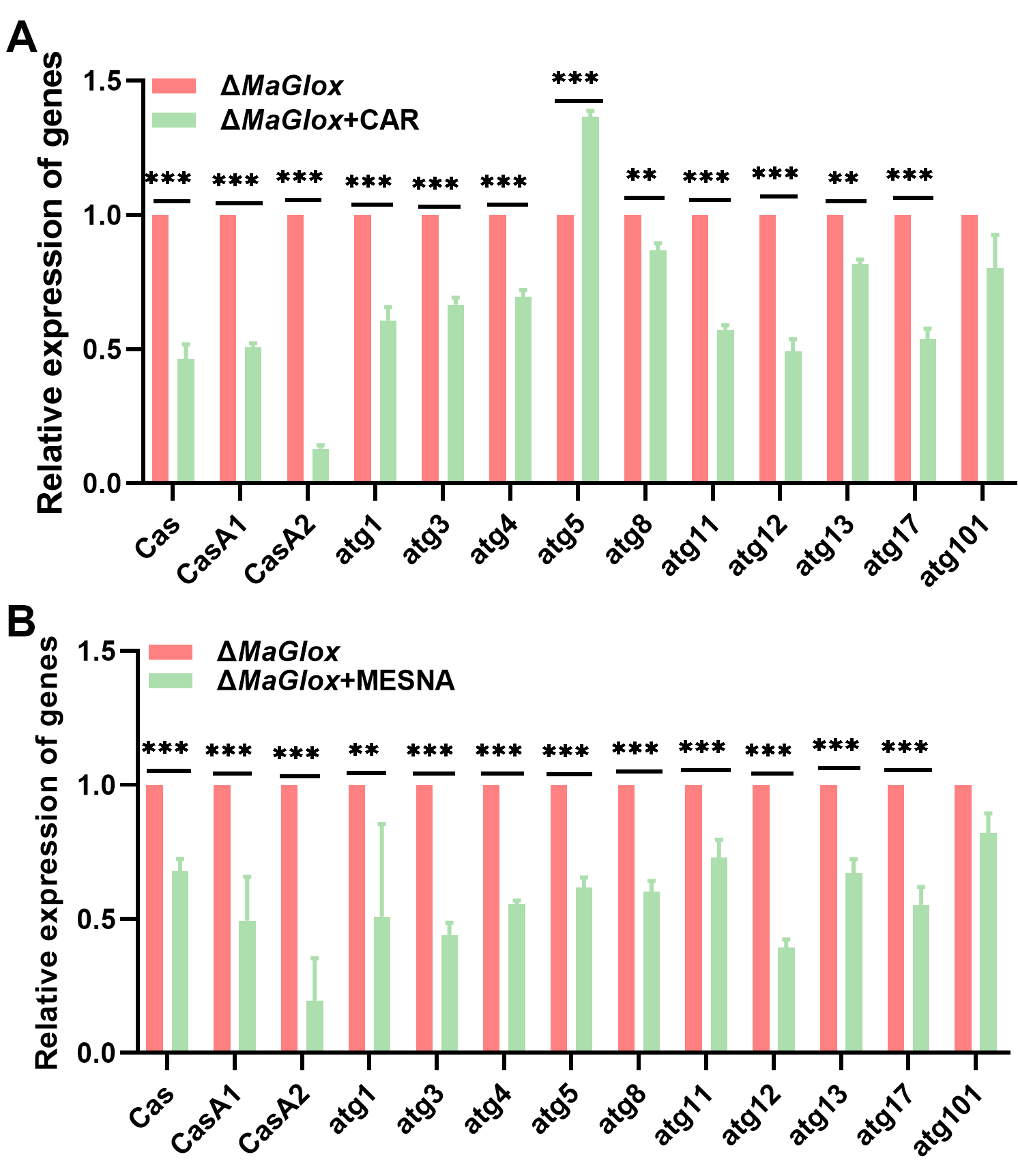

Supplement: S3 Fig — RT-qPCR analysis of the Cas and atg genes was conducted on the ΔMaGlox strains cultured on ¼-SDAY for 7 days, with or without the addition of CAR (A) and MESNA (B). (TIF) [file ppat.1012431.s004.tif]

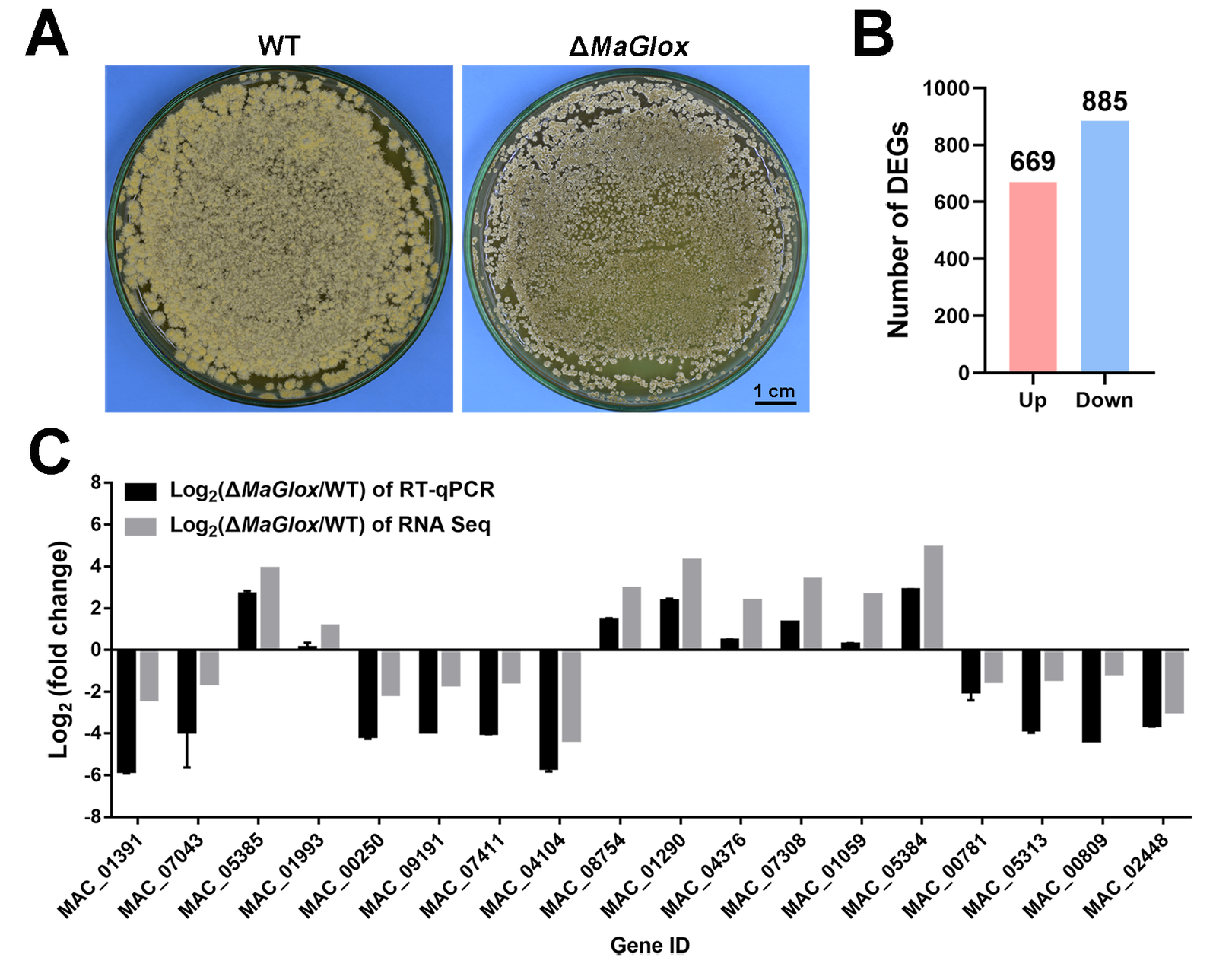

Supplement: S4 Fig — (A) Samples of wild type and ΔMaGlox on ¼-SDAY for RNA_Seq. (B) Number of DEGs in ΔMaGlox compared to WT. (C) Verification of RNA_Seq results by RT-qPCR. (TIF) [file ppat.1012431.s005.tif]

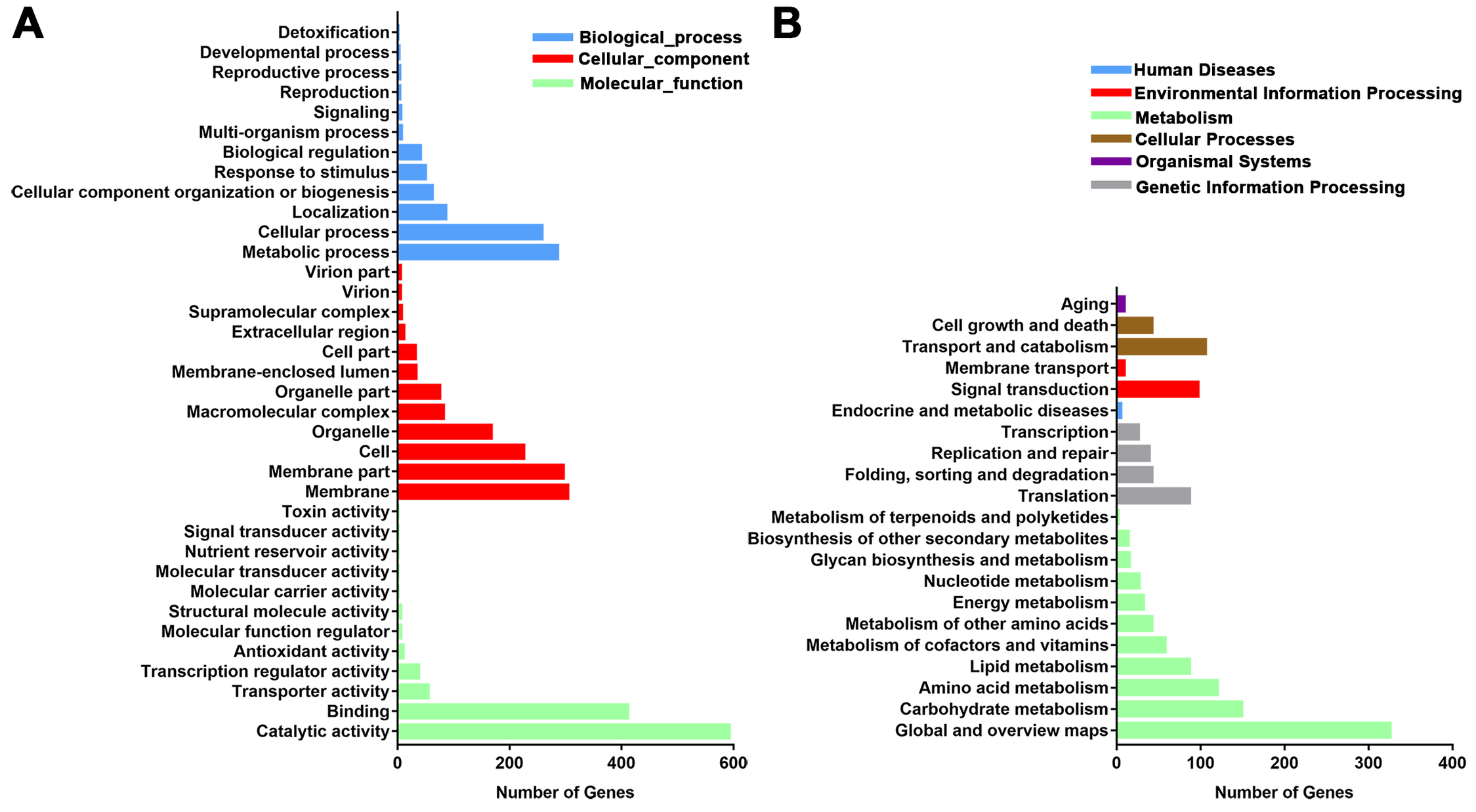

Supplement: S5 Fig — (A) GO classification of the DEGs from ΔMaGlox VS WT. (B) KEGG pathway classification of the DEGs from ΔMaGlox vs WT. (TIF) [file ppat.1012431.s006.tif]

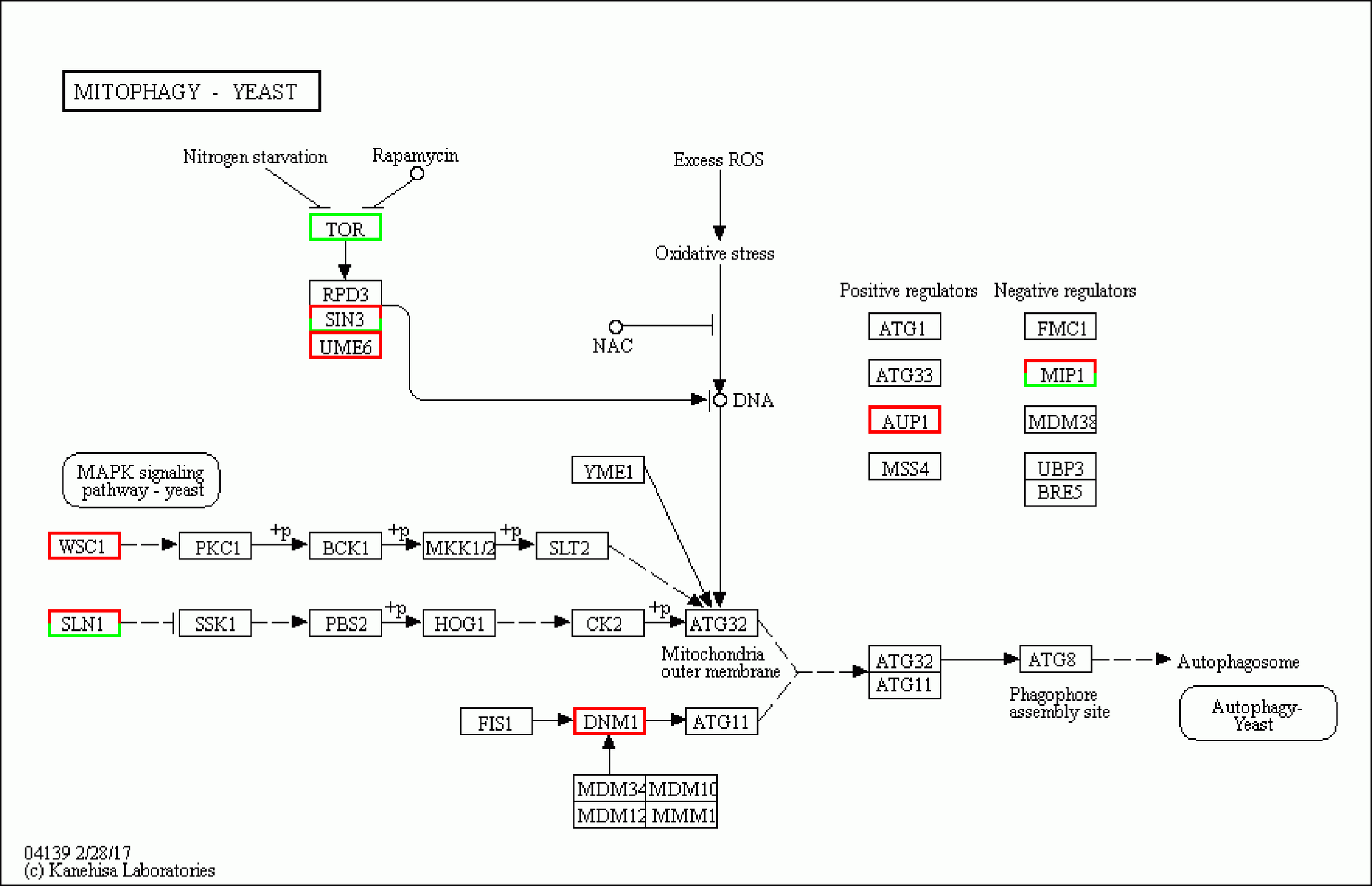

Supplement: S6 Fig — Green indicates downregulated DEGs and red indicates upregulated DEGs. (TIF) [file ppat.1012431.s007.tif]
