# Supplementary material for: Glyoxal oxidase-mediated detoxification of reactive carbonyl species contributes to virulence, stress tolerance, and development in a pathogenic fungus
Source: PLoS Pathog. 2024 Jul 30;20(7):e1012431. doi: 10.1371/journal.ppat.1012431 (PMC11315307; doi:10.1371/journal.ppat.1012431)
Supplement: S1 Table — (DOCX) [file ppat.1012431.s008.docx]

**S1 Table. Primers for vector construction, verification and RT-qPCR**.

| Primers | Sequence（5′-3′） | Remarks |
| --- | --- | --- |
| Glox_LF | gctctagagatgggctgctgcgagta (X*ba*I) | Construction of the Δ*MaGlox* mutant |
| Glox_LR | cggaattcccgcctgtgacatggtga (E*coR*I) |  |
| Glox_RF | gactagtactcaactggtgcttacgaact (S*pe*I) |  |
| Glox_RR | acgatatcggtctcaacacgatactcctcc (E*coR*V) |  |
| Glox_VF | aaaacagcaccaagccacg | Screening the disruption mutant |
| Pt-R | cagccaagcccaaaaagtg |  |
| Bar-F | gctctacacccacctgct |  |
| Glox_VR | cccatagagttgccgtgagt |  |
| CP-LF1 | gacggccagtgccaagctcttgacgccactcttcggta (*Hin*dⅢ) | Construction of the CP mutant |
| CP-LR1 | gtccgtgcctgaatcgtccgagctctctcacgtctgaatc |  |
| CP-LF2 | gattcagacgtgagagagctcggacgattcaggcacggac |  |
| CP-LR2 | ccatggcggccgggagcaaacgccgggagggttaaatcc (*Xba*I) |  |
| CP-RF | cgttggcacgtcgacgatactcaactggtgcttacgaact (EcoRⅤ) |  |
| CP-RR | atgacatgattacgaattggtctcaacacgatactcctcc (*EcoR*Ⅰ) |  |
| CP-VF | caagacgcttcccaacattc | Verification of the CP mutant |
| SurVL-R | atcaactcgtataggcaagg |  |
| SurVR-F | atcgtggagtcatgtttgcc |  |
| eGFP-VR | cgatgcggttcaccagggtgt | Screening and amplification of *MaGlox*-EGFP |
| OE-F | tacacacacgcaaatctagaatgagggcttcaccacttat (X*ba*I) |  |
| OE-R | ctcaccatactagtctcgagaacgccgggagggttaaatcca (X*ho*I) |  |
| Glox_PF | ccccaccccttgacagaat | Amplification the probe |
| Glox_PR | tcgcaactaggatggacgc | of *MaGlox* |

**Primers of genes for RT-qPCR**

| Glox_qF | cttcaccacttactcctt | RT-qPCR of *MaGlox* |
| --- | --- | --- |
| Glox_qR | ctcaacatagcaaccttg |  |
| MAC_04104-F | aacagattcaggctcaag | Calcium Channel Inhibitor |
| MAC_04104-R | gtagaaggcacagatagac |  |
| MAC_07411-F | tctcttctcaacatccag | Heat shock protein 70 |
| MAC_07411-R | tctaacaggttcgtatcaa |  |
| MAC_00250-F | gataggaaccgtcttgga | LEA domain containing protein |
| MAC_00250-R | tgtagttgtcgtcaatgtg |  |
| MAC_07043-F | gtcgaagttactgctctg | Glycerol-3-phosphate dehydrogenase |
| MAC_07043-R | ataatctccttgcgttgg |  |
| MAC_01391-F | atgaagaccaccattacg | Aspartyl protease |
| MAC_01391-R | ggacgatatgacgatgtag |  |
| MAC_02448-F | ggctaccactcttaccata | Clock-controlled protein |
| MAC_02448-R | gttgactccattgcttcc |  |
| MAC_05385-F | ccaagttctatcaatctgt | Conidial pigment polyketide synthase |
| MAC_05385-R | atatcaaaggcaaagacg |  |
| MAC_05313-F | gacatactcaccctcaag | Proliferating cell nuclear antigen |
| MAC_05313-R | catactcgctgattctgt |  |
| MAC_00809-F | agaagaagagaagactaacag | Serine/threonine-protein kinase PRKX |
| MAC_00809-R | cagaaggagcagatgatg |  |
| MAC_01993-F | acacatcaaccactcctt | Superoxide dismutase |
| MAC_01993-R | cccagaagagcctttgta |  |
| MAC_09191-F | cctcgtagtcattgtcttc | Hexose transporter-like protein |
| MAC_09191-R | tagtcatccgtgttccaa |  |
| MAC_01059-F | cgtatcagcagcatcact | Subtilisin-like serine protease PR1A |
| MAC_01059-R | caccagcactatcatcgta |  |
| MAC_08754-F | cgaaccgtaagatgaagac | Chitinase |
| MAC_08754-R | cataatagtgaccgctgatt |  |
| MAC_07308-F | ttggactctcggttctaat | Lectin |
| MAC_07308-R | gatctggtgtggtgattg |  |
| MAC_05384-F | cgattgagaatgctgtgat | Laccase |
| MAC_05384-R | ctcttcaaagtcctcgtt |  |
| MAC_04376-F | ctcttcaaagtcctcgtt | Hydrophobin |
| MAC_04376-R | gagatcttctggtggttg |  |
| MAC_01290-F | ctcacaccaaggaagtct | Mmc protein |
| MAC_01290-R | aggagggcgagaaaattc |  |
| MAC_00781-F | ccaagttctatcaatctgt | Phosphatidyl synthase |
| MAC_00781-R | atatcaaaggcaaagacg |  |
| MAC_07845-F | ttgacattataggatctc | CYP60B-like |
| MAC_07845-R | ctactaggattacgaatc |  |
| MAC_00270-F | aatacctcacagaacaagttg | CYP105-like |
| MAC_00270-R | gccgatataattcagcagtt |  |
| MAC_05399-F | ataatcggcgttcggtat | CYP_fungal |
| MAC_05399-R | cctctgtattcgtctggat |  |
| MAC_01110-F | ttgttcctcttggcttcc | CYP64-like |
| MAC_01110-R | tgatgattacttggcttcctt |  |
| MAC_01993-F | catgcctattacctacagta | SODA |
| MAC_01993-R | cttccagttgatgacctt |  |
| MAC_06152-F | agacaaggacaccaagac | Superoxide dismutase [Fe] |
| MAC_06152-R | ggaggcgtagttgaagat |  |
| MAC_01763-F | ctcctcttctgaacttcc | Manganese superoxide dismutase |
| MAC_01763-R | ccgaaatcctcattgatg |  |
| MAC_01194-F | agaagaagagaagactaacag | catalase |
| MAC_01194-R | cagaaggagcagatgatg |  |
| MAC_03712-F | acacatcaaccactcctt | catalase |
| MAC_03712-R | cccagaagagcctttgta |  |
| MAC_03117-F | cctcgtagtcattgtcttc | peroxisomal catalase |
| MAC_03117-R | tagtcatccgtgttccaa |  |
| MAC_04470-F | cgtatcagcagcatcact | catalase |
| MAC_04470-R | caccagcactatcatcgta |  |
| Cas-F | ctctgtcctacttcttgt | MAC_05111 caspase |
| Cas -R | atagatgacggtgtatgt |  |
| CasA1-F | cgatgtgattatgtggtc | MAC_04419 metacaspase CasA1 |
| CasA1-R | ctgaggcttctgagtata |  |
| CasA2-F | gcgtcatatcttcataca | MAC_04708 metacaspase CasA2 |
| CasA2-R | gtctggtcatctttactac |  |
| ATG1-F | cgaaccgtaagatgaagac | Autophagy-related protein 1 |
| ATG1-R | cataatagtgaccgctgatt |  |
| ATG3-F | caggtcactccagaagaa | Autophagy-related protein 3 |
| ATG3-R | tgtcacgaggtattgctt |  |
| ATG4-F | gagaataaggagccaact | Autophagy-related protein 4 |
| ATG4-R | ccagaacgaatcatacatc |  |
| ATG5-F | ctgaactgtgtcaaggag | Autophagy-related protein 5 |
| ATG5-R | cattcaacagcctcgtat |  |
| ATG8-F | gtagagaagagcgacatc | Autophagy-related protein 8 |
| ATG8-R | gcacctcatcaacgaata |  |
| ATG11-F | cagaaggagttgtatgtg | Autophagy-related protein 11 |
| ATG11-R | ggcgtttggtattgataa |  |
| ATG12-F | gtggttgtcaagttcaag | Autophagy-related protein 12 |
| ATG12-R | ctggtgtcatagagtagg |  |
| ATG13-F | gaactcaagatgtggaagac | Autophagy-related protein 13 |
| ATG13-R | aatgacaaggctctgact |  |
| ATG17-F | atattcagcagtcattcg | Autophagy-related protein 17 |
| ATG17-F | gggtaggattcgtttatg |  |
| ATG101-F | aacaaacccttctcacta | Autophagy-related protein 101 |
| ATG101-R | ttaatcttgaacgggaac |  |
