# Supplementary material for: Glyoxal oxidase-mediated detoxification of reactive carbonyl species contributes to virulence, stress tolerance, and development in a pathogenic fungus
Source: PLoS Pathog. 2024 Jul 30;20(7):e1012431. doi: 10.1371/journal.ppat.1012431 (PMC11315307; doi:10.1371/journal.ppat.1012431)
Supplement: S2 Table — (DOCX) [file ppat.1012431.s009.docx]

**S2 Table. Summary of aldehyde metabolites in *M. acridum*.**

| Analytes | CAS | Chemiccal formula after  DNPH derivatization | Calculated exact  mass [M-H]^-^ (*m*/*z*) | Measured mass [M-H]^-^ (m/z) | RT Time |
| --- | --- | --- | --- | --- | --- |
| Dimethylglyoxal | 431-03-8 | C16H14O8N8 | 445.0878 | 445.0862 | 21.2 |
| Methylglyoxal | 78-98-8 | C15H12O8N8 | 431.0726 | 431.0705 | 20.4 |
| Glyoxal | 107-22-2 | C14H10O8N8 | 417.0567 | 417.0549 | 19.7 |
| 4-Hydroxy-2-nonenal | 75899-68-2 | C15H20O5N4 | 335.1359 | 335.1361 | 18.8 |
| trans-2-Octenal | 2548-87-0 | C14H18O4N4 | 305.1266 | 305.1255 | 22.4 |
| 3-hydroxybenzaldehyde | 100-83-4 | C13H10O5N4 | 301.0581 | 301.0578 | 17.7 |
| p-Tolualdehyde | 104-87-0 | C14H12O4N4 | 299.0782 | 299.0786 | 19.9 |
| trans-2-Heptenal | 18829-55-5 | C13H16O4N4 | 291.1107 | 291.1099 | 21.6 |
| Benzaldehyde | 100-52-7 | C13H10O4N4 | 285.0636 | 285.0629 | 19.7 |
| trans, trans-2,4-Hexadienal | 142-83-6 | C12H12O4N4 | 275.0543 | 275.0786 | 11.1 |
| Pyruvic acid | 127-17-3 | C9H8O6N4 | 267.0388 | 267.0371 | 15.2 |
| Pentanal | 110-62-3 | C11H14O4N4 | 265.0941 | 265.0942 | 20.2 |
| 2,3-Butanedione | 431-03-8 | C10H10O5N4 | 265.0577 | 265.0578 | 17.5 |
| trans-2-Pentenal | 1576-87-0 | C11H12O4N4 | 263.0787 | 263.0786 | 16.7 |
| Propanal | 123-38-6 | C9H10O4N4 | 237.0633 | 237.0629 | 18.1 |
