# Supplementary material for: Glyoxal oxidase-mediated detoxification of reactive carbonyl species contributes to virulence, stress tolerance, and development in a pathogenic fungus
Source: PLoS Pathog. 2024 Jul 30;20(7):e1012431. doi: 10.1371/journal.ppat.1012431 (PMC11315307; doi:10.1371/journal.ppat.1012431)
Supplement: S3 Table — (DOCX) [file ppat.1012431.s010.docx]

**S3 Table. Differentially expressed genes (DEGs)**

| Gene ID | Protein name | log_2_(Δ*MaGlox*/WT) |
| --- | --- | --- |
| Cell cycle, division and growth | | |
| MAC_01832 | elongator complex protein | -2.1 |
| MAC_06614 | urea active transporter | -2.1 |
| MAC_04678 | CipC1 protein, concanamycin induced protein C | -1.9 |
| MAC_04110 | C6 transcription factor | -1.9 |
| MAC_02716 | cell division cycle protein | -1.9 |
| MAC_02282 | Ran1-like protein kinase | -1.8 |
| MAC_06356 | methyltransferase type 11 | -1.7 |
| MAC_02654 | HET domain protein | -1.7 |
| MAC_07406 | tRNA (His) guanylyltransferase | -1.6 |
| MAC_00637 | midasin | -1.6 |
| MAC_05600 | putative trfA protein | -1.6 |
| MAC_04682 | serine-type carboxypeptidase | -1.6 |
| MAC_03615 | putative SHK1 kinase-binding protein | -1.6 |
| MAC_01972 | DUF500 domain protein | -1.6 |
| MAC_01928 | Arginase | -1.5 |
| MAC_03738 | Peptidyl-prolyl cis-trans isomerase PIN4 | -1.5 |
| MAC_03828 | BRCA1 C Terminus (BRCT) domain containing protein | -1.5 |
| MAC_09570 | DEAD/DEAH box helicase | -1.4 |
| MAC_00854 | DEAD/DEAH box RNA helicase | -1.4 |
| MAC_01565 | nmt1 protein | -1.3 |
| MAC_03595 | putative anaphase promoting complex subunit 10 | -1.3 |
| MAC_03443 | Cell division control protein | -1.3 |
| MAC_07563 | Methyltransferase domain-containing protein | -1.2 |
| MAC_07123 | casein kinase II beta subunit | -1.2 |
| MAC_06726 | MAK16 protein | -1.2 |
| MAC_00300 | Glycosyl hydrolase family 76 | -1.2 |
| MAC_01024 | cell cycle control protein | -1.2 |
| MAC_08882 | Pfs, NACHT and Ankyrin domain protein | -1.1 |
| MAC_02730 | Ras guanyl-nucleotide exchange factor RasGEF | -1.1 |
| MAC_05808 | glycosyl transferase | -1 |
| MAC_01916 | Frequency clock protein | -1 |
| MAC_06466 | meiosis protein MEI2 | -1 |
| MAC_05865 | phosphoserine aminotransferase | -1 |
| MAC_05592 | pfs domain-containing protein | 1.05 |
| MAC_05522 | glycosyl hydrolase | 1.07 |
| MAC_07865 | glucosidase II alpha subunit | 1.12 |
| MAC_05276 | glycosyl hydrolase | 1.19 |
| MAC_07310 | homeoprotein | 1.2 |
| MAC_07651 | fumarate reductase Osm1 | 1.29 |
| MAC_05637 | O-methyltransferase | 1.29 |
| MAC_07927 | phosphoserine aminotransferase | 1.32 |
| MAC_00428 | REV7-like protein | 1.36 |
| MAC_08713 | methyltransferase | 1.54 |
| MAC_00274 | enoyl-CoA hydratase/carnithine racemase | 1.57 |
| MAC_00280 | glucose-6-phosphate isomerase | 1.58 |
| MAC_03833 | esdC | 1.72 |
| MAC_06218 | methyltransferase LaeA | 1.9 |
| MAC_03149 | putative SPO76 protein | 2.01 |
| MAC_09500 | methyltransferase LaeA | 2.25 |
| MAC_03279 | methyltransferase LaeA | 2.5 |
| MAC_04084 | Asp-hemolysin | 3.28 |
| MAC_08673 | ThiJ/PfpI family protein | 3.73 |
| MAC_01290 | Mmc protein | 4.28 |
| MAC_06640 | carbohydrate-binding protein | 5.67 |
| Oxidative stress | | |
| MAC_07440 | 2,5-diketo-D-gluconic acid reductase A | -3.8 |
| MAC_06606 | GMC oxidoreductase | -2.8 |
| MAC_02029 | MFS multidrug transporter | -2.6 |
| MAC_02945 | flavohemoprotein | -2.2 |
| MAC_01577 | C4-dicarboxylate transporter | -2.1 |
| MAC_05601 | hydrolase, TatD family protein | -1.8 |
| MAC_03503 | transporter protein smf2 | -1.8 |
| MAC_04470 | catalase | -1.6 |
| MAC_06407 | C4-dicarboxylate transporter | -1.4 |
| MAC_01488 | NADPH oxidase | -1.4 |
| MAC_06779 | NADPH-P450 reductase | -1.3 |
| MAC_06409 | flavodoxin and radical SAM domain protein | -1.2 |
| MAC_01223 | phosphotransferase family protein | -1.2 |
| MAC_02444 | phosphotransferase enzyme family protein | -1.2 |
| MAC_08909 | phosphotransferase enzyme family protein | -1.1 |
| MAC_02794 | Phosphotransferase family protein | -1.1 |
| MAC_08384 | NADP-specific glutamate dehydrogenase | -1.1 |
| MAC_03916 | FluG domain-containing protein | -1 |
| MAC_04849 | Superoxide dismutase | 1 |
| MAC_00155 | Hsp70 family chaperone | 1 |
| MAC_04434 | NADP-dependent leukotriene B4 12-hydroxydehydrogenase | 1.05 |
| MAC_03180 | peroxisomal dehydratase | 1.06 |
| MAC_08430 | oxidoreductase, 2-nitropropane dioxygenase family | 1.07 |
| MAC_08413 | alpha/beta hydrolase fold family protein | 1.11 |
| MAC_00174 | NADP-dependent alcohol dehydrogenase | 1.13 |
| MAC_01993 | Superoxide dismutase | 1.14 |
| MAC_01235 | oxidoreductase CipA-like | 1.14 |
| MAC_05578 | aldo/keto reductase/Endoribonuclease L-PSP | 1.15 |
| MAC_03727 | peroxin 26 | 1.25 |
| MAC_03471 | 2-nitropropane dioxygenase family oxidoreductase | 1.28 |
| MAC_06269 | Peroxidase/catalase | 1.28 |
| MAC_05628 | oxidoreductase, short-chain dehydrogenase/reductase family | 1.31 |
| MAC_08742 | flavohemoprotein | 1.43 |
| MAC_07495 | peroxisomal biogenesis factor 2 | 1.63 |
| MAC_05894 | oxidoreductase | 2.23 |
| MAC_02188 | hydrolase | 2.39 |
| MAC_04082 | MFS monocarboxylate transporter | 4.31 |
| Other stress response | | |
| MAC_02448 | clock-controlled protein | -2.9 |
| MAC_05829 | choline dehydrogenase | -2.3 |
| MAC_01249 | sulfate permease II | -2.2 |
| MAC_00250 | LEA domain containing protein | -2.1 |
| MAC_07589 | putative negative acting factor | -2.1 |
| MAC_07951 | C6 finger domain protein | -2 |
| MAC_07244 | putative multicopperoxidase | -1.9 |
| MAC_00249 | LEA domain protein | -1.8 |
| MAC_03963 | multidrug resistant protein | -1.8 |
| MAC_09749 | cytochrome P450 alkane hydroxylase | -1.7 |
| MAC_08930 | aquaporin | -1.6 |
| MAC_02378 | MFS multidrug transporter | -1.6 |
| MAC_01130 | spermidine synthase | -1.6 |
| MAC_07411 | heat shock protein 70 | -1.5 |
| MAC_06408 | DNA repair and recombination protein RAD5B | -1.5 |
| MAC_00106 | DNA repair protein Rad18 | -1.5 |
| MAC_09220 | C6 transcription factor | -1.5 |
| MAC_04242 | mitotic check point protein (Bub2) | -1.5 |
| MAC_06873 | MFS transporter | -1.5 |
| MAC_09279 | phosphorylcholine phosphatase | -1.4 |
| MAC_09287 | DUF775 domain protein | -1.3 |
| MAC_07024 | eliciting plant response-like protein | -1.3 |
| MAC_00726 | C6 finger domain protein | -1.3 |
| MAC_09141 | MFS maltose permease | -1.3 |
| MAC_01209 | metaphase-anaphase transition protein (Mlo2) | -1.2 |
| MAC_06025 | DNA repair protein Rad7, protein | -1.2 |
| MAC_08027 | penicillin-binding protein | -1.2 |
| MAC_00926 | heat shock 70 kd protein cognate 1 | -1.2 |
| MAC_06004 | C6 zinc finger domain protein | -1.2 |
| MAC_03740 | 40S ribosomal protein S22 | -1.1 |
| MAC_04632 | protein-tyrosine phosphatase 2 | -1.1 |
| MAC_02199 | MSF membrane transporter | -1.1 |
| MAC_05543 | PNGase family | -1.1 |
| MAC_06026 | DNA repair protein RAD16 | -1.1 |
| MAC_08820 | DNA repair protein Pso2/Snm1 | -1.1 |
| MAC_03397 | Carboxylesterase family protein | -1.1 |
| MAC_08691 | developmental protein FluG | -1.1 |
| MAC_02952 | penicillin-binding protein | -1.1 |
| MAC_04321 | DNA repair and recombination protein rhm52 | -1.1 |
| MAC_01221 | MFS monocarboxylate transporter | -1.1 |
| MAC_06114 | DEAD box ATP-dependent RNA helicase | -1 |
| MAC_01190 | penicillopepsin | -1 |
| MAC_08347 | 40S ribosomal protein S3 | -1 |
| MAC_04102 | increased rDNA silencing protein | -1 |
| MAC_05902 | carboxyphosphonoenolpyruvate phosphonomutase | 1.04 |
| MAC_02775 | DNA mismatch repair protein | 1.04 |
| MAC_08851 | Bys1 family protein | 1.1 |
| MAC_07906 | glutathione S-transferase II | 1.12 |
| MAC_03579 | protein wos2 | 1.13 |
| MAC_02789 | MFS transporter | 1.16 |
| MAC_03516 | toxin biosynthesis protein | 1.22 |
| MAC_09145 | carboxylesterase family protein | 1.26 |
| MAC_08932 | formate dehydrogenase | 1.27 |
| MAC_04341 | DUF636 domain protein | 1.28 |
| MAC_04714 | PH domain protein | 1.29 |
| MAC_00051 | Calcipressin family protein | 1.36 |
| MAC_05179 | GPR/FUN34 family protein | 1.41 |
| MAC_08283 | glutathione S-transferase II | 1.54 |
| MAC_08739 | carboxypeptidase | 1.73 |
| MAC_09405 | sorbitol dehydrogenase | 2.12 |
| MAC_06844 | Ig domain protein group 2 domain protein | 2.3 |
| MAC_02146 | stress responsive A/B barrel domain-containing protein | 3.12 |
| MAC_00831 | D-arabinitol dehydrogenase ArbD | 3.87 |
| MAC_08777 | 56kDa selenium binding protein (SBP56) | 4.1 |
| MAC_08008 | carboxylesterase family protein | 4.18 |
| Virulence | | |
| MAC_04104 | Calcium Channel Inhibitor | -4.3 |
| MAC_06826 | Flavin-binding monooxygenase-like family protein | -3 |
| MAC_03018 | DUF1275 domain protein | -3 |
| MAC_02271 | GrpB domain protein | -2.9 |
| MAC_07327 | chitinase | -2.9 |
| MAC_02485 | N,O-diacetyl muramidase | -2.9 |
| MAC_00632 | pyridine nucleotide-disulfide oxidoreductase | -2.1 |
| MAC_04340 | collagen-like protein Mcl1 | -2.1 |
| MAC_01893 | tyrosyl-DNA phosphodiesterase domain-containing protein | -2.1 |
| MAC_02167 | ABC transporter | -2 |
| MAC_07641 | Sugar transporter family protein | -1.9 |
| MAC_04194 | GTP binding protein | -1.9 |
| MAC_07031 | zinc transporter protein | -1.8 |
| MAC_00787 | ankyrin repeat domain containing protein | -1.8 |
| MAC_02142 | endo alpha-1,4 polygalactosaminidase precusor | -1.7 |
| MAC_07475 | Flavin-binding monooxygenase-like family protein | -1.6 |
| MAC_07043 | glycerol-3-phosphate dehydrogenase | -1.6 |
| MAC_09534 | kinase domain containing protein | -1.6 |
| MAC_03953 | molybdenum cofactor biosynthetic protein (CnxF) | -1.6 |
| MAC_01384 | WD repeat containing protein 36 | -1.5 |
| MAC_01579 | glutamate-rich WD repeat containing protein 1 | -1.5 |
| MAC_06248 | WD repeat-containing protein 75 | -1.4 |
| MAC_08425 | ABC ATPase | -1.3 |
| MAC_00105 | regulatory factor Sgt1 | -1.3 |
| MAC_01114 | eukaryotic aspartyl protease | -1.3 |
| MAC_02883 | Ankyrin | -1.3 |
| MAC_03788 | WD domain, G-beta repeat containing protein | -1.2 |
| MAC_07407 | WD domain-containing protein | -1.2 |
| MAC_04603 | ankyrin repeat containing protein | -1.2 |
| MAC_08821 | WD domain-containing protein | -1.2 |
| MAC_04538 | WD repeat protein | -1.2 |
| MAC_04314 | putative O-methyltransferase | -1.2 |
| MAC_00292 | WD domain containing protein | -1.1 |
| MAC_06226 | ABC transporter family protein | -1.1 |
| MAC_08059 | WD repeat protein | -1.1 |
| MAC_08579 | rRNA 2'-O-methyltransferase fibrillarin | -1.1 |
| MAC_01149 | Delta (24(24(1)))-sterol reductase | -1 |
| MAC_00811 | NUDIX domain-containing protein | -1 |
| MAC_01923 | histone acetyltransferase (MysT1) | -1 |
| MAC_05530 | translation Initiation Factor Eif4e | -1 |
| MAC_02006 | Lcc2 | 1.03 |
| MAC_00549 | WD domain containing protein | 1.21 |
| MAC_06221 | WD-repeat containing protein slp1 | 1.36 |
| MAC_02332 | ankyrin repeat protein | 1.43 |
| MAC_07780 | putative endochitinase CHI3 | 1.9 |
| MAC_02205 | chitinase 18-15 | 2.55 |
| MAC_07558 | major allergen Asp f 2-like protein | 2.65 |
| MAC_08754 | chitinase | 2.94 |
| MAC_05385 | conidial pigment polyketide synthase PksP/Alb1 | 3.9 |
| MAC_00860 | tetraspanin | 3.91 |
| MAC_07435 | ankyrin repeat-containing protein | 4 |
| MAC_07294 | cystein rich protein | 4.02 |
| MAC_05384 | laccase | 4.9 |
| Cell wall construction and modification | | |
| MAC_05040 | candidapepsin-4 precursor | -1.8 |
| MAC_03446 | sulfite reductase [NADPH] flavoprotein component | -1.8 |
| MAC_07319 | putative cell wall glycoprotein | -1.5 |
| MAC_00058 | putative beta (1-3) glucanosyltransferase | -1.4 |
| MAC_09140 | alpha-glucosidase | -1.4 |
| MAC_06491 | putative endo-1,3-1,4-b-glucanase | -1.4 |
| MAC_03760 | polysaccharide deacetylase (NodB) | -1.3 |
| MAC_07597 | beta-lactamase | -1.2 |
| MAC_05199 | alpha-1,2-mannosidase subfamily | -1.2 |
| MAC_07776 | alpha-1,2-mannosidase family protein | -1.1 |
| MAC_03886 | digestive-organ expansion factor | -1.1 |
| MAC_08880 | alpha-1,3-mannosyltransferase CMT1 | -1.1 |
| MAC_08711 | UPF0075 domain protein | -1 |
| MAC_07609 | TRP-like ion channel | -1 |
| MAC_07355 | GPI-anchored cell wall beta-1,3-endoglucanase EglC | 1.01 |
| MAC_02581 | calcofluor white hypersensitive protein | 1.04 |
| MAC_01544 | homogentisate 1,2-dioxygenase | 1.06 |
| MAC_07846 | calcium-transporting ATPase 3 | 1.07 |
| MAC_07330 | Hydrophobin-like protein ssgA | 1.17 |
| MAC_07374 | secreted glucosidase | 1.29 |
| MAC_03860 | GPI anchored serine-threonine rich protein | 1.43 |
| MAC_07466 | alpha-glucosidase | 1.51 |
| MAC_09507 | hydrophobin | 1.56 |
| MAC_06312 | gram-positive signal peptide, ysirk family | 1.72 |
| MAC_09531 | glucan 1,3-beta-glucosidase GLUC78 precursor | 1.84 |
| MAC_01035 | alpha-mannosidase | 1.91 |
| MAC_08097 | putative chitosanase CSN1 | 2.04 |
| MAC_04376 | hydrophobin | 2.36 |
| MAC_09499 | LysR family regulatory protein | 2.67 |
| MAC_05852 | antigenic cell wall galactomannoprotein | 2.85 |
| MAC_02204 | cell wall protein | 3.68 |
| MAC_09272 | alpha-1,2-mannosidase family protein | 3.73 |
| Cuticle degradation | | |
| MAC_01391 | aspartyl protease | -2.4 |
| MAC_06292 | cytochrome P450 monooxygenase | -1.7 |
| MAC_00270 | Cytochrome P450 55A2 | -1.5 |
| MAC_01942 | cytochrome P450 monooxygenase | -1.5 |
| MAC_06492 | CBM6-containing protein | -1.4 |
| MAC_00132 | OTU-like cysteine protease | -1.1 |
| MAC_09052 | Patatin family phospholipase | -1.1 |
| MAC_00542 | cytochrome c peroxidase precursor | 1.06 |
| MAC_00337 | cytochrome b5 | 1.14 |
| MAC_09597 | ubiquitin/metalloprotease fusion protein | 1.19 |
| MAC_01466 | vacuolar protease A | 1.33 |
| MAC_09005 | subtilisin-like protease Pr1B | 1.38 |
| MAC_05732 | cytochrome P450 | 1.43 |
| MAC_05727 | phospholipase D | 1.78 |
| MAC_03182 | Phosphatidylinositol-specific phospholipase C, X domain containing protein | 1.88 |
| MAC_05413 | Phosphatidylinositol-specific phospholipase | 1.9 |
| MAC_07120 | cytochrome P450 52A11 | 1.96 |
| MAC_01059 | Subtilisin-like serine protease PR1A | 2.63 |
| MAC_09477 | Cytochrome P450 family protein | 2.66 |
| MAC_06833 | trypsin-related protease | 4.05 |
| Lipid metabolism | | |
| MAC_00781 | phosphatidyl synthase | -1.5 |
| MAC_04113 | FAD binding domain-containing protein | -1.2 |
| MAC_04237 | FKBP-type peptidyl-prolyl isomerase | -1.1 |
| MAC_00353 | C-5 sterol desaturase | -1.1 |
| MAC_09350 | putative lipoprotein | -1.1 |
| MAC_01891 | phosphatidyl synthase | -1.1 |
| MAC_09268 | delta 8-(E)-sphingolipid desaturase | -1.1 |
| MAC_00735 | fatty acid activator Faa4 | -1.1 |
| MAC_07982 | PAP2 domain protein | -1 |
| MAC_02569 | FAD dependent oxidoreductase | 1.22 |
| MAC_00217 | acyl-CoA dehydrogenase | 1.22 |
| MAC_08195 | zinc-binding oxidoreductase ToxD | 1.33 |
| MAC_01470 | acyl-CoA dehydrogenase | 1.4 |
| MAC_07124 | PAP2 domain containing protein | 1.86 |
| MAC_01176 | 3-ketoacyl-CoA thiolase | 2.3 |
| Genetic information biosynthesis and processing | | |
| MAC_05977 | DNase1 protein | -2.5 |
| MAC_00657 | ARM-like repeat-containing protein | -2 |
| MAC_05264 | RNA-3'-phosphate cyclase family protein | -1.7 |
| MAC_03758 | transducin family protein | -1.7 |
| MAC_05929 | Ribosome biogenesis protein YTM1 | -1.6 |
| MAC_07107 | cutinase negative acting protein | -1.5 |
| MAC_01322 | GNAT family acetyltransferase | -1.5 |
| MAC_04096 | Sin3-associated polypeptide Sap18 | -1.5 |
| MAC_04009 | U3 snoRNP-associated protein Utp11 | -1.4 |
| MAC_05986 | 60S ribosomal subunit assembly/export protein loc1 | -1.4 |
| MAC_09772 | transglycosylase SLT domain protein | -1.4 |
| MAC_05313 | proliferating cell nuclear antigen | -1.4 |
| MAC_07462 | histone chaperone ASF1 | -1.4 |
| MAC_03994 | DNA-directed RNA polymerase III complex subunit Rpc37 | -1.4 |
| MAC_01287 | rRNA processing protein Bystin | -1.3 |
| MAC_01288 | origin recognition complex subunit Orc4 | -1.3 |
| MAC_02373 | putative Sulfite oxidase | -1.3 |
| MAC_00495 | DNA-directed RNA polymerase III RPC4 | -1.3 |
| MAC_04655 | ribosomal protein subunit S4 | -1.3 |
| MAC_00639 | CTD kinase subunit gamma | -1.3 |
| MAC_02918 | deacetylase complex subunit Sds3 | -1.3 |
| MAC_00633 | U3 small nucleolar RNA-associated protein | -1.3 |
| MAC_03371 | bZIP family transcription factor | -1.3 |
| MAC_09479 | U3 snoRNP protein | -1.3 |
| MAC_05524 | ribosome assembly protein Noc2 | -1.3 |
| MAC_00398 | 60S acidic ribosomal protein P0 | -1.3 |
| MAC_09242 | translation regulator GCD7 | -1.3 |
| MAC_08146 | 8-oxoguanine DNA glycosylase | -1.3 |
| MAC_03457 | cellulose signaling associated protein ENVOY | -1.3 |
| MAC_07581 | RNA binding protein | -1.3 |
| MAC_05776 | ribosomal RNA-processing protein 12 | -1.3 |
| MAC_05294 | RNA-binding protein of the Puf family, translational repressor | -1.2 |
| MAC_08747 | centrin-binding protein Sfi1 | -1.2 |
| MAC_00473 | Periodic tryptophan protein 2 | -1.2 |
| MAC_03639 | activator 1 subunit 3 | -1.2 |
| MAC_04733 | Copper fist DNA binding domain protein | -1.2 |
| MAC_01432 | RTA1 domain protein | -1.2 |
| MAC_07950 | U6 snRNA-associated protein LSm7 | -1.2 |
| MAC_03735 | 50S ribosomal subunit L30 | -1.2 |
| MAC_00110 | MIF4G domain containing protein | -1.2 |
| MAC_02379 | ribonuclease H1 | -1.2 |
| MAC_05886 | transposase | -1.2 |
| MAC_04699 | ribosome biogenesis protein Rrp14-C | -1.2 |
| MAC_09020 | exosomal core protein CSL4 | -1.2 |
| MAC_05267 | PinX1-related protein | -1.2 |
| MAC_03829 | APSES transcription factor | -1.2 |
| MAC_02262 | nucleolar protein NOP52 variant | -1.2 |
| MAC_05994 | chromatin assembly factor 1 subunit C | -1.2 |
| MAC_09802 | dependent RNA helicase drs-1 | -1.2 |
| MAC_01945 | Pre-mRNA processing ribonucleoprotein, snoRNA-binding domain protein, partial | -1.2 |
| MAC_07393 | LSM domain-containing protein | -1.2 |
| MAC_02633 | small nucleolar ribonucleoprotein complex subunit Utp14, | -1.2 |
| MAC_01316 | rRNA assembly protein Mis3 | -1.2 |
| MAC_03899 | small nucleolar ribonucleoprotein complex subunit Utp15 | -1.2 |
| MAC_00727 | rRNA processing protein Rrp8 | -1.2 |
| MAC_00506 | putative translation initiation factor 4e | -1.1 |
| MAC_01804 | pre-rRNA processing protein Tsr1 | -1.1 |
| MAC_00475 | NAP family protein | -1.1 |
| MAC_05295 | RWD domain protein (Gir2) | -1.1 |
| MAC_07841 | pre-rRNA-processing protein ESF1 | -1.1 |
| MAC_07232 | transcription initiation factor TFIID subunit 13 | -1.1 |
| MAC_09564 | ribosome biogenesis protein (Bms1) | -1.1 |
| MAC_05023 | threonyl-tRNA synthetase | -1.1 |
| MAC_00017 | histidinol-phosphatase | -1.1 |
| MAC_02318 | putative DNA directed DNA polymerase II chain B | -1.1 |
| MAC_07265 | pescadillo | -1.1 |
| MAC_09755 | RNA recognition motif containing protein | -1.1 |
| MAC_04691 | eukaryotic translation initiation factor subunit eIF2A | -1.1 |
| MAC_03411 | snRNP and snoRNP protein (Snu13) | -1.1 |
| MAC_07627 | ribonuclease P complex subunit Pop2 | -1.1 |
| MAC_00305 | MYND finger family protein | -1.1 |
| MAC_05081 | putative chromatin assembly complex, subunit p90 | -1.1 |
| MAC_01614 | telomere length regulation protein elg1 | -1.1 |
| MAC_03757 | pre-mRNA splicing factor ATP-dependent RNA helicase prp16 | -1.1 |
| MAC_03625 | histone acetylase complex subunit MRG15-2 | -1.1 |
| MAC_04828 | Mediator of RNA polymerase II transcription subunit 31 | -1.1 |
| MAC_00734 | C2H2 transcription factor | -1.1 |
| MAC_06548 | activator 1 38 kDa subunit | -1.1 |
| MAC_04499 | zinc knuckle domain containing protein | -1.1 |
| MAC_05660 | RNA polymerase Rpb1 repeat domain protein | -1.1 |
| MAC_03544 | putative transcriptional repressor | -1.1 |
| MAC_03205 | nucleolar protein 12 | -1.1 |
| MAC_06477 | eukaryotic translation initiation factor 3 subunit CLU1/TIF31 | -1.1 |
| MAC_09571 | tRNA (cytosine-5-)-methyltransferase NCL1 | -1.1 |
| MAC_00349 | snoRNP protein (gar1) | -1.1 |
| MAC_01803 | small nucleolar ribonucleoprotein complex subunit | -1.1 |
| MAC_06440 | transcription factor spTFIIE alpha subunit | -1.1 |
| MAC_07532 | fungal specific transcription factor | -1.1 |
| MAC_05542 | RING-finger protein | -1.1 |
| MAC_04380 | Pre-mRNA-splicing factor ISY1 | -1.1 |
| MAC_06742 | Rpp14 family | -1.1 |
| MAC_03573 | ribose-phosphate pyrophosphokinase | -1 |
| MAC_02795 | nonselective cation channel | -1 |
| MAC_00242 | electron transfer flavoprotein alpha-subunit | -1 |
| MAC_01947 | putative 40S ribosomal protein | -1 |
| MAC_07588 | exosome-associated family protein | -1 |
| MAC_07451 | rRNA processing protein Nop9 | -1 |
| MAC_01315 | 60S ribosomal protein L19 precursor | -1 |
| MAC_00921 | putative SEN1 protein | -1 |
| MAC_03597 | nucleolar protein NOP58-like protein | -1 |
| MAC_02111 | 66S ribosomal complex subunit | -1 |
| MAC_06784 | RNA-binding La domain protein | -1 |
| MAC_06180 | pre-mRNA-processing protein prp40 | -1 |
| MAC_00531 | 60S ribosomal protein L36 | -1 |
| MAC_02535 | 3' exoribonuclease | -1 |
| MAC_00641 | histone acetyltransferase type B catalytic subunit | -1 |
| MAC_02525 | ribosome biogenesis protein Kri1 | -1 |
| MAC_01302 | norsolorinic acid reductase | -1 |
| MAC_06229 | putative lysyl-tRNA synthetase (lysine--tRNA ligase) | -1 |
| MAC_00827 | transcriptional regulator (Cti6) | -1 |
| MAC_05051 | nuclear export protein Noc3 | -1 |
| MAC_01562 | transposase | 1.07 |
| MAC_00185 | nucleoside-diphosphate-sugar epimerase family protein | 1.07 |
| MAC_01348 | RNA binding protein MSSP-2 | 1.11 |
| MAC_06966 | glycyl-tRNA synthetase 1 | 1.15 |
| MAC_07250 | cysteine synthase A | 1.15 |
| MAC_08573 | C2H2 finger domain protein | 1.15 |
| MAC_08402 | Cutinase transcription factor 1 beta | 1.18 |
| MAC_09307 | Ctf1 transcription factor | 1.23 |
| MAC_07254 | von Willebrand factor | 1.29 |
| MAC_02718 | GNAT family acetyltransferase | 1.29 |
| MAC_00150 | pol protein | 1.3 |
| MAC_03555 | transcription factor TFIIH subunit Tfb4 | 1.32 |
| MAC_09544 | Cutinase gene palindrome-binding protein | 1.36 |
| MAC_05728 | exonuclease III | 1.44 |
| MAC_04477 | acetyltransferase | 1.46 |
| MAC_05773 | histidine acid phosphatase | 1.63 |
| MAC_03195 | cytidine deaminase | 1.65 |
| MAC_07121 | L-kynurenine/alpha-aminoadipate aminotransferase | 1.73 |
| MAC_08135 | CTR2 long splice variant | 1.76 |
| MAC_07469 | 5-oxoprolinase | 1.81 |
| MAC_08588 | nucleoside transporter | 2.2 |
| MAC_04169 | HMG box protein | 2.41 |
| MAC_01474 | histone H1 | 3.27 |
| Catalytic activity | | |
| MAC_06925 | acyl-CoA oxidase | -1.7 |
| MAC_06613 | xenobiotic compound monooxygenase, DszA family | -1.7 |
| MAC_04725 | phosphoenolpyruvate carboxykinase | -1.7 |
| MAC_06403 | amidohydrolase | -1.7 |
| MAC_09729 | serine/threonine protein kinase Sgk2 | -1.6 |
| MAC_04014 | triadin | -1.5 |
| MAC_02562 | U3 small nucleolar ribonucleoprotein protein IMP3 | -1.5 |
| MAC_05505 | D-isomer specific 2-hydroxyacid dehydrogenase | -1.5 |
| MAC_07643 | phospho-2-dehydro-3-deoxyheptonate aldolase | -1.5 |
| MAC_05300 | protein arginine N-methyltransferase 1 | -1.5 |
| MAC_08587 | ubiquitin carboxyl-terminal hydrolase 2 | -1.5 |
| MAC_04599 | Alternative oxidase | -1.4 |
| MAC_06543 | PQ loop repeat protein | -1.4 |
| MAC_02677 | chlorocatechol 1,2-dioxygenase | -1.4 |
| MAC_07955 | Multicopper oxidase family protein | -1.4 |
| MAC_05847 | V8-like Glu-specific endopeptidase | -1.4 |
| MAC_00532 | thymidylate synthase | -1.3 |
| MAC_06893 | short-chain dehydrogenase/reductase | -1.3 |
| MAC_06202 | inosine-5'-monophosphate dehydrogenase IMD2 | -1.3 |
| MAC_05424 | conserved fungal protein | -1.3 |
| MAC_09457 | Putative ATPase family protein | -1.3 |
| MAC_01312 | endoglucanase | -1.3 |
| MAC_02567 | putative dCMP deaminase | -1.3 |
| MAC_07331 | cholinesterase | -1.3 |
| MAC_05026 | DUF453 domain-containing protein | -1.2 |
| MAC_02365 | PAF acetylhydrolase family protein | -1.2 |
| MAC_06094 | phosphoadenosine phosphosulfate reductase | -1.2 |
| MAC_03473 | phytanoyl-CoA dioxygenase | -1.2 |
| MAC_00871 | ethanolaminephosphotransferase | -1.2 |
| MAC_02757 | siderophore biosynthesis enzyme | -1.2 |
| MAC_08798 | putative N-acetyl-glutamate semialdehyde dehydrogenase, precursor | -1.2 |
| MAC_00640 | pyrroline-5-carboxylate reductase | -1.2 |
| MAC_09301 | putative protein arginine N-methyltransferase 3 | -1.2 |
| MAC_05521 | aspartic-type endopeptidase (OpsB) | -1.2 |
| MAC_07482 | lysyl-tRNA synthetase | -1.2 |
| MAC_03644 | ADP, ATP carrier protein | -1.2 |
| MAC_00435 | vacuolar protein sorting-associated protein 62 | -1.2 |
| MAC_06766 | guanine deaminase | -1.2 |
| MAC_07816 | Inorganic pyrophosphatase | -1.2 |
| MAC_08298 | acetolactate synthase | -1.2 |
| MAC_06264 | putative d-aminopeptidase | -1.2 |
| MAC_00846 | aspartate-semialdehyde dehydrogenase | -1.2 |
| MAC_06668 | putative nuclear pore protein NUP57 | -1.1 |
| MAC_00809 | serine/threonine-protein kinase PRKX | -1.1 |
| MAC_03144 | nitrate reductase | -1.1 |
| MAC_05263 | pentatricopeptide repeat containing protein | -1.1 |
| MAC_03353 | paraoxonase | -1.1 |
| MAC_07516 | SNF2 family helicase/ATPase | -1.1 |
| MAC_07594 | ubiquinone biosynthesis protein coq-4 | -1.1 |
| MAC_01817 | serine-threonine rich protein | -1.1 |
| MAC_03945 | secreted protein | -1.1 |
| MAC_01186 | thiazole biosynthetic enzyme variant 1 | -1.1 |
| MAC_09193 | Multicopper oxidase family protein | -1.1 |
| MAC_01109 | cystathionine gamma-synthase | -1.1 |
| MAC_08348 | DUF803 domain membrane protein | -1.1 |
| MAC_07473 | pyridoxine | -1.1 |
| MAC_04622 | calmodulin-dependent protein kinase | -1.1 |
| MAC_09185 | epoxide hydrolase | -1.1 |
| MAC_06092 | DUF1690 domain-containing protein | -1.1 |
| MAC_01955 | putative oxidoreductase | -1.1 |
| MAC_04851 | transaldolase | -1.1 |
| MAC_00724 | Spc97 / Spc98 family protein | -1.1 |
| MAC_07511 | OPA3 domain protein | -1.1 |
| MAC_07247 | Protein phosphatase 2C domain containing protein | -1.1 |
| MAC_05639 | amine oxidase | -1.1 |
| MAC_06048 | UDP-glucose,sterol transferase | -1.1 |
| MAC_01796 | alpha-1,2-galactosyltransferase-like protein | -1.1 |
| MAC_02583 | dimethyladenosine transferase dimethyltransferase | -1 |
| MAC_00481 | gamma-tubulin complex component GCP5 | -1 |
| MAC_07856 | multisynthetase complex auxiliary component p43 | -1 |
| MAC_04608 | prolyl-tRNA synthetase | -1 |
| MAC_08168 | sulfite reductase beta subunit | -1 |
| MAC_00915 | acetylornithine deacetylase | -1 |
| MAC_07817 | UPF0145 domain-containing protein | -1 |
| MAC_05222 | peptidyl-tRNA hydrolase domain protein | -1 |
| MAC_07806 | cyclin-dependent protein kinase complex component | -1 |
| MAC_00739 | tyrosyl-DNA phosphodiesterase | -1 |
| MAC_04961 | sulfatase domain-containing protein | 1 |
| MAC_01601 | carboxy-cis,cis-muconate cyclase | 1 |
| MAC_06837 | Rieske [2Fe-2S] domain protein | 1.01 |
| MAC_07363 | amidohydrolase family protein | 1.01 |
| MAC_09337 | adenosylhomocysteinase | 1.03 |
| MAC_02819 | 2,3-dihydroxybenzoic acid decarboxylase dhbD | 1.04 |
| MAC_05705 | Phosphoglycerate kinase | 1.04 |
| MAC_04092 | Xaa-Pro dipeptidase | 1.07 |
| MAC_03447 | sarcosine oxidase | 1.1 |
| MAC_03500 | N-acetylglucosamine-6-phosphate deacetylase | 1.12 |
| MAC_06282 | N-acetylglucosaminidase | 1.16 |
| MAC_03923 | Putative Acyl-CoA dehydrogenase | 1.17 |
| MAC_03069 | protein HMF1 | 1.18 |
| MAC_07543 | short-chain dehydrogenase | 1.19 |
| MAC_02826 | 1-phosphatidylinositol phosphodiesterase precursor | 1.19 |
| MAC_04360 | tripsin | 1.19 |
| MAC_09685 | short chain dehydrogenase | 1.21 |
| MAC_05128 | acyl-coenzyme A oxidase | 1.23 |
| MAC_05717 | ThiJ/PfpI domain-containing protein | 1.26 |
| MAC_02360 | Glyoxalase/bleomycin resistance protein/dioxygenase | 1.27 |
| MAC_07047 | putative acyl-CoA dehydrogenas | 1.28 |
| MAC_04378 | magnesium-translocating P-type ATPase family protein | 1.29 |
| MAC_08779 | sn-1,2-diacylglycerol cholinephosphotransferase | 1.3 |
| MAC_04015 | mannosylphosphorylation protein (Mnn4) | 1.3 |
| MAC_03416 | peroxisomal membrane anchor protein (Pex14) | 1.36 |
| MAC_04393 | putative nuclease S1 precursor | 1.37 |
| MAC_00755 | putative exo-alpha-sialidase / neuraminidase | 1.38 |
| MAC_05152 | quinone oxidoreductase | 1.41 |
| MAC_09460 | carbohydrate-binding module family 13 protein | 1.45 |
| MAC_00140 | feruloyl esterase | 1.45 |
| MAC_03852 | neutral/alkaline nonlysosomal ceramidase | 1.47 |
| MAC_09584 | glyceraldehyde-3-phosphate dehydrogenase | 1.47 |
| MAC_08412 | TRI14-like protein | 1.48 |
| MAC_05007 | 4-aminobutyrate aminotransferase | 1.5 |
| MAC_07359 | maleylacetoacetate isomerase | 1.51 |
| MAC_02385 | Pyruvate kinase | 1.52 |
| MAC_09442 | long-chain-fatty-acid--CoA ligase FAA2 | 1.55 |
| MAC_05465 | kynureninase | 1.56 |
| MAC_08032 | glutaminase GtaA | 1.6 |
| MAC_02839 | mannose-6-phosphate isomerase | 1.66 |
| MAC_02084 | acid sphingomyelinase | 1.67 |
| MAC_01254 | lipoxygenase | 1.72 |
| MAC_09167 | nitroreductase family protein | 1.72 |
| MAC_03098 | annexin XIV | 1.73 |
| MAC_09498 | aminoadipate-semialdehyde dehydrogenase | 1.73 |
| MAC_05697 | serum paraoxonase/arylesterase family protein | 1.75 |
| MAC_04886 | acyl-CoA thioesterase | 1.75 |
| MAC_07506 | serine family amino acid catabolism-related protein | 1.78 |
| MAC_06995 | putative glutathione S-transferase | 1.84 |
| MAC_01048 | phosphomannomutase | 1.89 |
| MAC_07348 | inner membrane transport protein yeiJ | 1.91 |
| MAC_06832 | thiolase | 1.94 |
| MAC_01760 | guanyl-specific ribonuclease F1 | 1.95 |
| MAC_08267 | putative BCS1 protein precursor | 1.95 |
| MAC_00550 | exo-beta-1,3-glucanase | 1.96 |
| MAC_09033 | pyruvate decarboxylase | 1.98 |
| MAC_04762 | Golgi membrane protein | 2.02 |
| MAC_03211 | streptomycin biosynthesis protein StrI | 2.04 |
| MAC_03198 | putative histidine acid phosphatase | 2.05 |
| MAC_08973 | aldehyde reductase (GliO) | 2.11 |
| MAC_07572 | extracellular serine-rich protein | 2.14 |
| MAC_04016 | protein-ER retention receptor | 2.17 |
| MAC_04155 | beta-xylosidase | 2.24 |
| MAC_03585 | cobalamin-independent methionine synthase | 2.34 |
| MAC_08098 | endoglucanase | 2.53 |
| MAC_08073 | Multifunctional beta-oxidation protein | 2.58 |
| MAC_05632 | feruloyl esterase B precursor | 2.61 |
| MAC_06834 | sarcosine oxidase | 2.61 |
| MAC_05464 | indoleamine 2,3-dioxygenase pyrrole 2,3-dioxygenase | 2.66 |
| MAC_02622 | indoleamine 2,3-dioxygenase-like protein | 2.74 |
| MAC_09355 | BAR domain containing protein | 2.9 |
| MAC_00167 | extracellular dioxygenase | 2.97 |
| MAC_09822 | leupeptin-inactivating enzyme 1 precursor | 3.22 |
| MAC_02015 | 4-hydroxyphenylpyruvate dioxygenase | 3.25 |
| MAC_04410 | lysine amidinotransferase | 3.29 |
| MAC_09773 | phosphatidylglycerol/phosphatidylinositol transfer protein | 3.35 |
| MAC_08696 | endo-N-acetyl-beta-D-glucosaminidase precursor | 3.38 |
| MAC_09497 | polyketide synthase | 3.4 |
| Transport | | |
| MAC_09139 | integral membrane protein | -1.8 |
| MAC_09191 | hexose transporter-like protein | -1.7 |
| MAC_01298 | mitochondrial carrier protein | -1.5 |
| MAC_05827 | integral membrane protein | -1.4 |
| MAC_05171 | phosphate permease | -1.4 |
| MAC_03484 | poly(A) RNA binding protein | -1.3 |
| MAC_00494 | integral membrane family protein | -1.3 |
| MAC_03776 | integral membrane protein | -1.3 |
| MAC_05979 | GARP complex component (Vps54) | -1.3 |
| MAC_04288 | siroheme synthase Met8 | -1.3 |
| MAC_08939 | Mitochondrial import inner membrane translocase subunit tim-22 | -1.2 |
| MAC_09259 | MDR efflux pump ABC3 | -1.2 |
| MAC_08910 | oligopeptide transporter | -1.2 |
| MAC_07853 | Transmembrane amino acid transporter family protein | -1.2 |
| MAC_05028 | AIG2 family protein | -1.2 |
| MAC_03351 | importin beta-4 subunit | -1.1 |
| MAC_06908 | sulfate transporter | -1.1 |
| MAC_03716 | mitochondrial distribution and morphology protein 34 | -1.1 |
| MAC_05917 | mitochondrial folate carrier protein Flx1 | -1.1 |
| MAC_00491 | mitochondrial carrier protein | -1.1 |
| MAC_06410 | mitochondrial import receptor subunit tom-20 | -1.1 |
| MAC_02559 | pantothenate transporter liz1 | -1.1 |
| MAC_07952 | mitochondrial carrier protein RIM2 | -1.1 |
| MAC_08325 | mitochondrial oxaloacetate transport protein | -1.1 |
| MAC_08687 | arylsulfatase precursor | -1.1 |
| MAC_00663 | AP-2 complex subunit beta | -1 |
| MAC_04213 | cation diffusion facilitator 10 | -1 |
| MAC_00825 | anion exchange protein | -1 |
| MAC_03655 | AP-3 complex subunit sigma | -1 |
| MAC_08527 | monooxygenase FAD-binding protein | -1 |
| MAC_04333 | integral membrane protein | -1 |
| MAC_07788 | Ctr copper transporter | 1.07 |
| MAC_01901 | transporter | 1.07 |
| MAC_08996 | Ctr copper transporter family protein | 1.19 |
| MAC_00768 | DUF895 domain protein | 1.28 |
| MAC_02711 | putative calcium P-type ATPase | 1.38 |
| MAC_00176 | DUF895 domain membrane protein | 1.38 |
| MAC_09175 | protein CCC1 | 1.52 |
| MAC_04254 | acyltransferase | 1.66 |
| MAC_02056 | NIPSNAP family protein | 1.67 |
| MAC_05950 | plasma membrane calcium-transporting ATPase 2 | 1.68 |
| MAC_07309 | putative P-type ATPase | 1.68 |
| MAC_03559 | iron/copper transporter Atx1 | 1.91 |
| MAC_09810 | membrane transporter | 2.02 |
| MAC_08221 | fumarylacetoacetate hydrolase family protein | 2.03 |
| MAC_07796 | galactose-proton symport | 2.08 |
| MAC_03586 | putative fluconazole resistance protein (FLU1) | 2.23 |
| MAC_08470 | charged multivesicular body protein 3 | 2.29 |
| MAC_09520 | amino acid transporter | 2.42 |
| MAC_06568 | integral membrane protein | 2.43 |
| Hypothetical protein | | |
| MAC_04105 | hypothetical protein MAC_04105 | -3.94 |
| MAC_07744 | hypothetical protein MAC_07744 | -3.68 |
| MAC_04106 | hypothetical protein MAC_04106 | -2.76 |
| MAC_02232 | hypothetical protein MAC_02232 | -2.6 |
| MAC_06307 | hypothetical protein MAC_06307 | -2.57 |
| MAC_02129 | hypothetical protein MAC_02129 | -2.43 |
| MAC_08006 | hypothetical protein MAC_08006 | -2.42 |
| MAC_04601 | hypothetical protein MAC_04601 | -2.31 |
| MAC_05216 | hypothetical protein MAC_05216 | -2.3 |
| MAC_06216 | hypothetical protein MAC_06216 | -2.28 |
| MAC_07619 | hypothetical protein MAC_07619 | -2.23 |
| MAC_06771 | hypothetical protein MAC_06771 | -2.12 |
| MAC_02486 | hypothetical protein MAC_02486 | -2.02 |
| MAC_00261 | hypothetical protein MAC_00261 | -1.95 |
| MAC_02230 | hypothetical protein MAC_02230 | -1.9 |
| MAC_05869 | hypothetical protein MAC_05869 | -1.89 |
| MAC_02653 | hypothetical protein MAC_02653 | -1.88 |
| MAC_05867 | hypothetical protein MAC_05867 | -1.88 |
| MAC_00903 | hypothetical protein MAC_00903 | -1.81 |
| MAC_05412 | hypothetical protein MAC_05412 | -1.8 |
| MAC_00396 | hypothetical protein MAC_00396 | -1.76 |
| MAC_03243 | hypothetical protein MAC_03243 | -1.76 |
| MAC_04545 | hypothetical protein MAC_04545 | -1.75 |
| MAC_02137 | hypothetical protein MAC_02137 | -1.74 |
| MAC_04546 | hypothetical protein MAC_04546 | -1.71 |
| MAC_03855 | hypothetical protein MAC_03855 | -1.68 |
| MAC_01575 | hypothetical protein MAC_01575 | -1.66 |
| MAC_07745 | hypothetical protein MAC_07745 | -1.66 |
| MAC_00961 | hypothetical protein MAC_00961 | -1.65 |
| MAC_01934 | hypothetical protein MAC_01934 | -1.62 |
| MAC_00966 | hypothetical protein MAC_00966 | -1.61 |
| MAC_09137 | hypothetical protein MAC_09137 | -1.58 |
| MAC_01927 | hypothetical protein MAC_01927 | -1.57 |
| MAC_04332 | hypothetical protein MAC_04332 | -1.57 |
| MAC_04222 | hypothetical protein MAC_04222 | -1.53 |
| MAC_07840 | hypothetical protein MAC_07840 | -1.52 |
| MAC_08194 | hypothetical protein MAC_08194 | -1.52 |
| MAC_05666 | hypothetical protein MAC_05666 | -1.5 |
| MAC_01326 | hypothetical protein MAC_01326 | -1.5 |
| MAC_07663 | hypothetical protein MAC_07663 | -1.49 |
| MAC_03456 | hypothetical protein MAC_03456 | -1.49 |
| MAC_02670 | hypothetical protein MAC_02670 | -1.48 |
| MAC_00246 | hypothetical protein MAC_00246 | -1.48 |
| MAC_02850 | hypothetical protein MAC_02850 | -1.48 |
| MAC_05914 | hypothetical protein MAC_05914 | -1.47 |
| MAC_07607 | hypothetical protein MAC_07607 | -1.47 |
| MAC_08223 | hypothetical protein MAC_08223 | -1.47 |
| MAC_07518 | hypothetical protein MAC_07518 | -1.46 |
| MAC_02419 | hypothetical protein MAC_02419 | -1.44 |
| MAC_05729 | hypothetical protein MAC_05729 | -1.44 |
| MAC_03194 | hypothetical protein MAC_03194 | -1.43 |
| MAC_08970 | hypothetical protein MAC_08970 | -1.4 |
| MAC_05580 | hypothetical protein MAC_05580 | -1.4 |
| MAC_04586 | hypothetical protein MAC_04586 | -1.39 |
| MAC_08585 | hypothetical protein MAC_08585 | -1.38 |
| MAC_05599 | hypothetical protein MAC_05599 | -1.38 |
| MAC_09264 | hypothetical protein MAC_09264 | -1.37 |
| MAC_00459 | hypothetical protein MAC_00459 | -1.37 |
| MAC_07196 | hypothetical protein MAC_07196 | -1.36 |
| MAC_05824 | hypothetical protein MAC_05824 | -1.36 |
| MAC_03232 | hypothetical protein MAC_03232 | -1.36 |
| MAC_01943 | hypothetical protein MAC_01943 | -1.35 |
| MAC_07639 | hypothetical protein MAC_07639 | -1.35 |
| MAC_01615 | hypothetical protein MAC_01615 | -1.34 |
| MAC_02358 | hypothetical protein MAC_02358 | -1.33 |
| MAC_02001 | hypothetical protein MAC_02001 | -1.32 |
| MAC_03022 | hypothetical protein MAC_03022 | -1.3 |
| MAC_06676 | hypothetical protein MAC_06676 | -1.3 |
| MAC_01540 | hypothetical protein MAC_01540 | -1.3 |
| MAC_01477 | hypothetical protein MAC_01477 | -1.29 |
| MAC_09747 | hypothetical protein MAC_09747 | -1.29 |
| MAC_03779 | hypothetical protein MAC_03779 | -1.28 |
| MAC_09243 | hypothetical protein MAC_09243 | -1.28 |
| MAC_09325 | hypothetical protein MAC_09325 | -1.28 |
| MAC_02060 | hypothetical protein MAC_02060 | -1.27 |
| MAC_00835 | hypothetical protein MAC_00835 | -1.27 |
| MAC_04245 | hypothetical protein MAC_04245 | -1.26 |
| MAC_04764 | hypothetical protein MAC_04764 | -1.26 |
| MAC_09328 | hypothetical protein MAC_09328 | -1.25 |
| MAC_05866 | hypothetical protein MAC_05866 | -1.23 |
| MAC_07186 | hypothetical protein MAC_07186 | -1.23 |
| MAC_00662 | hypothetical protein MAC_00662 | -1.22 |
| MAC_08605 | hypothetical protein MAC_08605 | -1.22 |
| MAC_00104 | hypothetical protein MAC_00104 | -1.2 |
| MAC_07459 | hypothetical protein MAC_07459 | -1.2 |
| MAC_01541 | hypothetical protein MAC_01541 | -1.2 |
| MAC_00455 | hypothetical protein MAC_00455 | -1.2 |
| MAC_05447 | hypothetical protein MAC_05447 | -1.2 |
| MAC_00970 | hypothetical protein MAC_00970 | -1.19 |
| MAC_00392 | hypothetical protein MAC_00392 | -1.19 |
| MAC_03398 | hypothetical protein MAC_03398 | -1.19 |
| MAC_02073 | hypothetical protein MAC_02073 | -1.18 |
| MAC_04918 | hypothetical protein MAC_04918 | -1.17 |
| MAC_03406 | hypothetical protein MAC_03406 | -1.17 |
| MAC_04225 | hypothetical protein MAC_04225 | -1.17 |
| MAC_07851 | hypothetical protein MAC_07851 | -1.17 |
| MAC_07199 | hypothetical protein MAC_07199 | -1.17 |
| MAC_07828 | hypothetical protein MAC_07828 | -1.17 |
| MAC_04731 | hypothetical protein MAC_04731 | -1.16 |
| MAC_07372 | hypothetical protein MAC_07372 | -1.16 |
| MAC_04005 | hypothetical protein MAC_04005 | -1.16 |
| MAC_03342 | hypothetical protein MAC_03342 | -1.16 |
| MAC_06413 | hypothetical protein MAC_06413 | -1.15 |
| MAC_00758 | hypothetical protein MAC_00758 | -1.15 |
| MAC_05644 | hypothetical protein MAC_05644 | -1.15 |
| MAC_02474 | hypothetical protein MAC_02474 | -1.15 |
| MAC_07278 | hypothetical protein MAC_07278 | -1.14 |
| MAC_01656 | hypothetical protein MAC_01656 | -1.14 |
| MAC_06397 | hypothetical protein MAC_06397 | -1.13 |
| MAC_02229 | hypothetical protein MAC_02229 | -1.13 |
| MAC_00520 | hypothetical protein MAC_00520 | -1.13 |
| MAC_06232 | hypothetical protein MAC_06232 | -1.13 |
| MAC_04745 | hypothetical protein MAC_04745 | -1.13 |
| MAC_04423 | hypothetical protein MAC_04423 | -1.12 |
| MAC_07947 | hypothetical protein MAC_07947 | -1.11 |
| MAC_00920 | hypothetical protein MAC_00920 | -1.11 |
| MAC_04446 | hypothetical protein MAC_04446 | -1.11 |
| MAC_09214 | hypothetical protein MAC_09214 | -1.1 |
| MAC_08530 | hypothetical protein MAC_08530 | -1.1 |
| MAC_00430 | hypothetical protein MAC_00430 | -1.09 |
| MAC_08834 | hypothetical protein MAC_08834 | -1.09 |
| MAC_05540 | hypothetical protein MAC_05540 | -1.09 |
| MAC_04233 | hypothetical protein MAC_04233 | -1.09 |
| MAC_05002 | hypothetical protein MAC_05002 | -1.09 |
| MAC_08576 | hypothetical protein MAC_08576 | -1.08 |
| MAC_08935 | hypothetical protein MAC_08935 | -1.08 |
| MAC_04858 | hypothetical protein MAC_04858 | -1.08 |
| MAC_00805 | hypothetical protein MAC_00805 | -1.08 |
| MAC_06123 | hypothetical protein MAC_06123 | -1.07 |
| MAC_09603 | hypothetical protein MAC_09603 | -1.07 |
| MAC_06147 | hypothetical protein MAC_06147 | -1.07 |
| MAC_06542 | hypothetical protein MAC_06542 | -1.07 |
| MAC_08357 | hypothetical protein MAC_08357 | -1.06 |
| MAC_04700 | hypothetical protein MAC_04700 | -1.06 |
| MAC_01724 | hypothetical protein MAC_01724 | -1.06 |
| MAC_09601 | hypothetical protein MAC_09601 | -1.06 |
| MAC_02030 | hypothetical protein MAC_02030 | -1.05 |
| MAC_05198 | hypothetical protein MAC_05198 | -1.05 |
| MAC_01270 | hypothetical protein MAC_01270 | -1.05 |
| MAC_02554 | hypothetical protein MAC_02554 | -1.05 |
| MAC_04965 | hypothetical protein MAC_04965 | -1.05 |
| MAC_03689 | hypothetical protein MAC_03689 | -1.05 |
| MAC_01714 | hypothetical protein MAC_01714 | -1.05 |
| MAC_03993 | hypothetical protein MAC_03993 | -1.05 |
| MAC_03657 | hypothetical protein MAC_03657 | -1.05 |
| MAC_02793 | hypothetical protein MAC_02793 | -1.05 |
| MAC_07131 | hypothetical protein MAC_07131 | -1.04 |
| MAC_04890 | hypothetical protein MAC_04890 | -1.04 |
| MAC_06680 | hypothetical protein MAC_06680 | -1.04 |
| MAC_03440 | hypothetical protein MAC_03440 | -1.04 |
| MAC_05612 | hypothetical protein MAC_05612 | -1.04 |
| MAC_03752 | hypothetical protein MAC_03752 | -1.04 |
| MAC_08359 | hypothetical protein MAC_08359 | -1.04 |
| MAC_07249 | hypothetical protein MAC_07249 | -1.03 |
| MAC_08323 | hypothetical protein MAC_08323 | -1.03 |
| MAC_02345 | hypothetical protein MAC_02345 | -1.03 |
| MAC_08155 | hypothetical protein MAC_08155 | -1.03 |
| MAC_07288 | hypothetical protein MAC_07288 | -1.03 |
| MAC_09533 | hypothetical protein MAC_09533 | -1.02 |
| MAC_09285 | hypothetical protein MAC_09285 | -1.02 |
| MAC_05638 | hypothetical protein MAC_05638 | -1.02 |
| MAC_07048 | hypothetical protein MAC_07048 | -1.02 |
| MAC_08388 | hypothetical protein MAC_08388 | -1.01 |
| MAC_04651 | hypothetical protein MAC_04651 | -1 |
| MAC_09809 | hypothetical protein MAC_09809 | -1 |
| MAC_01839 | hypothetical protein MAC_01839 | 1 |
| MAC_03517 | hypothetical protein MAC_03517 | 1.04 |
| MAC_02152 | hypothetical protein MAC_02152 | 1.05 |
| MAC_04227 | hypothetical protein MAC_04227 | 1.05 |
| MAC_08794 | hypothetical protein MAC_08794 | 1.05 |
| MAC_04830 | hypothetical protein MAC_04830 | 1.06 |
| MAC_04183 | hypothetical protein MAC_04183 | 1.06 |
| MAC_05876 | hypothetical protein MAC_05876 | 1.06 |
| MAC_01545 | hypothetical protein MAC_01545 | 1.08 |
| MAC_02234 | hypothetical protein MAC_02234 | 1.1 |
| MAC_08284 | hypothetical protein MAC_08284 | 1.11 |
| MAC_04304 | hypothetical protein MAC_04304 | 1.11 |
| MAC_04761 | hypothetical protein MAC_04761 | 1.13 |
| MAC_06381 | hypothetical protein MAC_06381 | 1.13 |
| MAC_02860 | hypothetical protein MAC_02860 | 1.15 |
| MAC_04264 | hypothetical protein MAC_04264 | 1.16 |
| MAC_08940 | hypothetical protein MAC_08940 | 1.16 |
| MAC_00672 | hypothetical protein MAC_00672 | 1.18 |
| MAC_06240 | hypothetical protein MAC_06240 | 1.19 |
| MAC_03651 | hypothetical protein MAC_03651 | 1.2 |
| MAC_02361 | hypothetical protein MAC_02361 | 1.2 |
| MAC_00214 | hypothetical protein MAC_00214 | 1.21 |
| MAC_06263 | hypothetical protein MAC_06263 | 1.22 |
| MAC_04488 | hypothetical protein MAC_04488 | 1.22 |
| MAC_09468 | hypothetical protein MAC_09468 | 1.23 |
| MAC_01091 | hypothetical protein MAC_01091 | 1.23 |
| MAC_08031 | hypothetical protein MAC_08031 | 1.23 |
| MAC_00262 | hypothetical protein MAC_00262 | 1.24 |
| MAC_05640 | hypothetical protein MAC_05640 | 1.24 |
| MAC_01450 | hypothetical protein MAC_01450 | 1.25 |
| MAC_04258 | hypothetical protein MAC_04258 | 1.25 |
| MAC_05895 | hypothetical protein MAC_05895 | 1.27 |
| MAC_04570 | hypothetical protein MAC_04570 | 1.27 |
| MAC_09260 | hypothetical protein MAC_09260 | 1.28 |
| MAC_02013 | hypothetical protein MAC_02013 | 1.28 |
| MAC_05629 | hypothetical protein MAC_05629 | 1.29 |
| MAC_02259 | hypothetical protein MAC_02259 | 1.3 |
| MAC_09469 | hypothetical protein MAC_09469 | 1.32 |
| MAC_08778 | hypothetical protein MAC_08778 | 1.32 |
| MAC_07344 | hypothetical protein MAC_07344 | 1.32 |
| MAC_04684 | hypothetical protein MAC_04684 | 1.36 |
| MAC_05949 | hypothetical protein MAC_05949 | 1.37 |
| MAC_07570 | hypothetical protein MAC_07570 | 1.38 |
| MAC_08491 | hypothetical protein MAC_08491 | 1.39 |
| MAC_01904 | hypothetical protein MAC_01904 | 1.39 |
| MAC_00873 | hypothetical protein MAC_00873 | 1.39 |
| MAC_01172 | hypothetical protein MAC_01172 | 1.42 |
| MAC_01722 | hypothetical protein MAC_01722 | 1.45 |
| MAC_06961 | hypothetical protein MAC_06961 | 1.45 |
| MAC_05071 | hypothetical protein MAC_05071 | 1.47 |
| MAC_04313 | hypothetical protein MAC_04313 | 1.47 |
| MAC_03407 | hypothetical protein MAC_03407 | 1.47 |
| MAC_08541 | hypothetical protein MAC_08541 | 1.48 |
| MAC_06367 | hypothetical protein MAC_06367 | 1.52 |
| MAC_00424 | hypothetical protein MAC_00424 | 1.52 |
| MAC_00489 | hypothetical protein MAC_00489 | 1.52 |
| MAC_06965 | hypothetical protein MAC_06965 | 1.54 |
| MAC_09470 | hypothetical protein MAC_09470 | 1.57 |
| MAC_04876 | hypothetical protein MAC_04876 | 1.58 |
| MAC_08268 | hypothetical protein MAC_08268 | 1.58 |
| MAC_05054 | hypothetical protein MAC_05054 | 1.59 |
| MAC_02556 | hypothetical protein MAC_02556 | 1.65 |
| MAC_01543 | hypothetical protein MAC_01543 | 1.66 |
| MAC_09057 | hypothetical protein MAC_09057 | 1.69 |
| MAC_05488 | hypothetical protein MAC_05488 | 1.69 |
| MAC_00294 | hypothetical protein MAC_00294 | 1.7 |
| MAC_08845 | hypothetical protein MAC_08845 | 1.74 |
| MAC_02548 | hypothetical protein MAC_02548 | 1.77 |
| MAC_09566 | hypothetical protein MAC_09566 | 1.78 |
| MAC_06672 | hypothetical protein MAC_06672 | 1.85 |
| MAC_07873 | hypothetical protein MAC_07873 | 1.96 |
| MAC_07329 | hypothetical protein MAC_07329 | 2 |
| MAC_07507 | hypothetical protein MAC_07507 | 2.02 |
| MAC_06704 | hypothetical protein MAC_06704 | 2.04 |
| MAC_00516 | hypothetical protein MAC_00516 | 2.06 |
| MAC_04780 | hypothetical protein MAC_04780 | 2.12 |
| MAC_01401 | hypothetical protein MAC_01401 | 2.14 |
| MAC_05908 | hypothetical protein MAC_05908 | 2.15 |
| MAC_02330 | hypothetical protein MAC_02330 | 2.2 |
| MAC_04420 | hypothetical protein MAC_04420 | 2.21 |
| MAC_03312 | hypothetical protein MAC_03312 | 2.28 |
| MAC_02590 | hypothetical protein MAC_02590 | 2.31 |
| MAC_04799 | hypothetical protein MAC_04799 | 2.34 |
| MAC_01715 | hypothetical protein MAC_01715 | 2.39 |
| MAC_09682 | hypothetical protein MAC_09682 | 2.43 |
| MAC_06604 | hypothetical protein MAC_06604 | 2.51 |
| MAC_07465 | hypothetical protein MAC_07465 | 2.53 |
| MAC_06380 | hypothetical protein MAC_06380 | 2.57 |
| MAC_01556 | hypothetical protein MAC_01556 | 2.61 |
| MAC_09352 | hypothetical protein MAC_09352 | 2.63 |
| MAC_04396 | hypothetical protein MAC_04396 | 2.75 |
| MAC_03362 | hypothetical protein MAC_03362 | 2.88 |
| MAC_04955 | hypothetical protein MAC_04955 | 2.96 |
| MAC_04763 | hypothetical protein MAC_04763 | 3 |
| MAC_02603 | hypothetical protein MAC_02603 | 3.3 |
| MAC_02158 | hypothetical protein MAC_02158 | 3.35 |
| MAC_07308 | hypothetical protein MAC_07308 | 3.37 |
| MAC_03809 | hypothetical protein MAC_03809 | 3.46 |
| MAC_02481 | hypothetical protein MAC_02481 | 3.75 |
| MAC_04031 | hypothetical protein MAC_04031 | 3.87 |
| MAC_02506 | hypothetical protein MAC_02506 | 3.91 |
| MAC_05976 | hypothetical protein MAC_05976 | 4.1 |
| MAC_09732 | hypothetical protein MAC_09732 | 4.21 |
| MAC_05095 | hypothetical protein MAC_05095 | 4.24 |
| MAC_02623 | hypothetical protein MAC_02623 | 4.31 |
| MAC_04391 | hypothetical protein MAC_04391 | 4.32 |
| MAC_05715 | hypothetical protein MAC_05715 | 4.34 |
| MAC_09586 | hypothetical protein MAC_09586 | 4.42 |
| MAC_08604 | hypothetical protein MAC_08604 | 4.56 |
| MAC_04454 | hypothetical protein MAC_04454 | 4.66 |
| MAC_08927 | hypothetical protein MAC_08927 | 4.72 |
| MAC_05692 | hypothetical protein MAC_05692 | 5.04 |
| MAC_08016 | hypothetical protein MAC_08016 | 5.18 |
| MAC_06126 | hypothetical protein MAC_06126 | 5.33 |
| MAC_05207 | hypothetical protein MAC_05207 | 6.11 |
| MAC_05723 | hypothetical protein MAC_05723 | 6.58 |
| MAC_07870 | hypothetical protein MAC_07870 | 6.83 |
