# Supplementary material for: Glyoxal oxidase-mediated detoxification of reactive carbonyl species contributes to virulence, stress tolerance, and development in a pathogenic fungus
Source: PLoS Pathog. 2024 Jul 30;20(7):e1012431. doi: 10.1371/journal.ppat.1012431 (PMC11315307; doi:10.1371/journal.ppat.1012431)
Supplement: S4 Table — (DOCX) [file ppat.1012431.s011.docx]

**S4 Table. DEGs related to virulence queried against the PHI**.

| Gene ID | Protein name | log_2_(Δ*MaGlox*/WT) |
| --- | --- | --- |
| MAC_06826 | Flavin-binding monooxygenase-like family protein | -3.04 |
| MAC_06606 | GMC oxidoreductase | -2.79 |
| MAC_02029 | MFS multidrug transporter | -2.62 |
| MAC_01391 | aspartyl protease | -2.36 |
| MAC_05829 | choline dehydrogenase | -2.32 |
| MAC_02945 | flavohemoprotein | -2.16 |
| MAC_00632 | pyridine nucleotide-disulfide oxidoreductase | -2.14 |
| MAC_07589 | putative negative acting factor | -2.09 |
| MAC_02486 | hypothetical protein MAC_02486 | -2.02 |
| MAC_02167 | ABC transporter | -2.01 |
| MAC_07951 | C6 finger domain protein | -2 |
| MAC_07244 | putative multicopperoxidase | -1.92 |
| MAC_04110 | C6 transcription factor | -1.91 |
| MAC_07641 | Sugar transporter family protein | -1.91 |
| MAC_02653 | hypothetical protein MAC_02653 | -1.88 |
| MAC_05601 | hydrolase, TatD family protein | -1.84 |
| MAC_07031 | zinc transporter protein | -1.84 |
| MAC_05040 | candidapepsin-4 precursor | -1.8 |
| MAC_05412 | hypothetical protein MAC_05412 | -1.8 |
| MAC_00787 | ankyrin repeat domain containing protein | -1.76 |
| MAC_02282 | Ran1-like protein kinase | -1.76 |
| MAC_03963 | multidrug resistant protein | -1.75 |
| MAC_06292 | cytochrome P450 monooxygenase | -1.73 |
| MAC_09749 | cytochrome P450 alkane hydroxylase | -1.73 |
| MAC_04725 | phosphoenolpyruvate carboxykinase | -1.69 |
| MAC_03758 | transducin family protein | -1.68 |
| MAC_09191 | hexose transporter-like protein | -1.67 |
| MAC_01575 | hypothetical protein MAC_01575 | -1.66 |
| MAC_00961 | hypothetical protein MAC_00961 | -1.65 |
| MAC_02378 | MFS multidrug transporter | -1.61 |
| MAC_07043 | glycerol-3-phosphate dehydrogenase | -1.61 |
| MAC_05929 | Ribosome biogenesis protein YTM1 | -1.59 |
| MAC_04470 | catalase | -1.58 |
| MAC_09534 | kinase domain containing protein | -1.57 |
| MAC_03615 | putative SHK1 kinase-binding protein | -1.56 |
| MAC_01130 | spermidine synthase | -1.55 |
| MAC_01384 | WD repeat containing protein 36 | -1.54 |
| MAC_07411 | heat shock protein 70 | -1.53 |
| MAC_06408 | DNA repair and recombination protein RAD5B | -1.53 |
| MAC_00270 | Cytochrome P450 55A2 | -1.52 |
| MAC_03738 | Peptidyl-prolyl cis-trans isomerase PIN4 | -1.49 |
| MAC_03456 | hypothetical protein MAC_03456 | -1.49 |
| MAC_01579 | glutamate-rich WD repeat containing protein 1 | -1.48 |
| MAC_09220 | C6 transcription factor | -1.48 |
| MAC_04242 | mitotic check point protein (Bub2) | -1.47 |
| MAC_05300 | protein arginine N-methyltransferase 1 | -1.47 |
| MAC_01942 | cytochrome P450 monooxygenase | -1.45 |
| MAC_00058 | putative beta (1-3) glucanosyltransferase | -1.44 |
| MAC_09140 | alpha-glucosidase | -1.42 |
| MAC_05580 | hypothetical protein MAC_05580 | -1.4 |
| MAC_01488 | NADPH oxidase | -1.38 |
| MAC_07462 | histone chaperone ASF1 | -1.38 |
| MAC_00854 | DEAD/DEAH box RNA helicase | -1.38 |
| MAC_05171 | phosphate permease | -1.38 |
| MAC_07955 | Multicopper oxidase family protein | -1.35 |
| MAC_07639 | hypothetical protein MAC_07639 | -1.35 |
| MAC_01288 | origin recognition complex subunit Orc4 | -1.34 |
| MAC_02373 | putative Sulfite oxidase | -1.34 |
| MAC_06202 | 5'-monophosphate dehydrogenase IMD2 | -1.31 |
| MAC_03484 | poly(A) RNA binding protein | -1.3 |
| MAC_01540 | hypothetical protein MAC_01540 | -1.3 |
| MAC_00494 | integral membrane family protein | -1.29 |
| MAC_03371 | bZIP family transcription factor | -1.29 |
| MAC_01477 | hypothetical protein MAC_01477 | -1.29 |
| MAC_01312 | endoglucanase | -1.28 |
| MAC_07024 | eliciting plant response-like protein | -1.28 |
| MAC_00726 | C6 finger domain protein | -1.28 |
| MAC_09141 | MFS maltose permease | -1.27 |
| MAC_06779 | NADPH-P450 reductase | -1.27 |
| MAC_03760 | polysaccharide deacetylase (NodB) | -1.26 |
| MAC_07331 | cholinesterase | -1.26 |
| MAC_03457 | cellulose signaling associated protein ENVOY | -1.26 |
| MAC_00105 | regulatory factor Sgt1 | -1.26 |
| MAC_03443 | Cell division control protein | -1.25 |
| MAC_02883 | Ankyrin | -1.25 |
| MAC_00473 | Periodic tryptophan protein 2 | -1.22 |
| MAC_03639 | activator 1 subunit 3 | -1.22 |
| MAC_06025 | DNA repair protein Rad7, protein | -1.21 |
| MAC_08027 | penicillin-binding protein | -1.2 |
| MAC_08821 | WD domain-containing protein | -1.2 |
| MAC_08798 | putative N-acetyl-glutamate semialdehyde dehydrogenase, precursor | -1.2 |
| MAC_07597 | beta-lactamase | -1.2 |
| MAC_09259 | MDR efflux pump ABC3 | -1.2 |
| MAC_00926 | heat shock 70 kd protein cognate 1 | -1.19 |
| MAC_09301 | putative protein arginine N-methyltransferase 3 | -1.19 |
| MAC_03398 | hypothetical protein MAC_03398 | -1.19 |
| MAC_04538 | WD repeat protein | -1.18 |
| MAC_06004 | C6 zinc finger domain protein | -1.18 |
| MAC_05521 | aspartic-type endopeptidase (OpsB) | -1.18 |
| MAC_07482 | lysyl-tRNA synthetase | -1.18 |
| MAC_03644 | ADP, ATP carrier protein | -1.17 |
| MAC_09020 | exosomal core protein CSL4 | -1.17 |
| MAC_03406 | hypothetical protein MAC_03406 | -1.17 |
| MAC_07851 | hypothetical protein MAC_07851 | -1.17 |
| MAC_03829 | APSES transcription factor | -1.16 |
| MAC_05994 | chromatin assembly factor 1 subunit C | -1.16 |
| MAC_01024 | cell cycle control protei | -1.16 |
| MAC_09802 | dependent RNA helicase drs-1 | -1.16 |
| MAC_08298 | acetolactate synthase | -1.16 |
| MAC_01316 | rRNA assembly protein Mis3 | -1.15 |
| MAC_03899 | small nucleolar ribonucleoprotein complex subunit Utp15 | -1.15 |
| MAC_04632 | protein-tyrosine phosphatase 2 | -1.14 |
| MAC_02199 | MSF membrane transporter | -1.14 |
| MAC_00475 | NAP family protein | -1.14 |
| MAC_08909 | phosphotransferase enzyme family protein | -1.13 |
| MAC_00809 | serine/threonine-protein kinase PRKX | -1.13 |
| MAC_04237 | FKBP-type peptidyl-prolyl isomerase | -1.13 |
| MAC_00292 | WD domain containing protein | -1.13 |
| MAC_06226 | ABC transporter family protein | -1.13 |
| MAC_02229 | hypothetical protein MAC_02229 | -1.13 |
| MAC_06232 | hypothetical protein MAC_06232 | -1.13 |
| MAC_07265 | pescadillo | -1.12 |
| MAC_03144 | nitrate reductase | -1.12 |
| MAC_09052 | Patatin family phospholipase | -1.11 |
| MAC_07516 | SNF2 family helicase/ATPase | -1.11 |
| MAC_06026 | DNA repair protein RAD16 | -1.11 |
| MAC_03757 | pre-mRNA splicing factor ATP-dependent RNA helicase prp16 | -1.1 |
| MAC_05917 | mitochondrial folate carrier protein Flx1 | -1.1 |
| MAC_00491 | mitochondrial carrier protein | -1.1 |
| MAC_00353 | C-5 sterol desaturase | -1.1 |
| MAC_09193 | Multicopper oxidase family protein | -1.09 |
| MAC_00734 | C2H2 transcription factor | -1.09 |
| MAC_03397 | Carboxylesterase family protein | -1.09 |
| MAC_08348 | DUF803 domain membrane protein | -1.09 |
| MAC_08059 | WD repeat protein | -1.09 |
| MAC_08834 | hypothetical protein MAC_08834 | -1.09 |
| MAC_04499 | zinc knuckle domain containing protein | -1.08 |
| MAC_03544 | putative transcriptional repressor | -1.08 |
| MAC_04622 | calmodulin-dependent protein kinase | -1.08 |
| MAC_02559 | pantothenate transporter liz1 | -1.08 |
| MAC_07952 | mitochondrial carrier protein RIM2 | -1.07 |
| MAC_09571 | RNA (cytosine-5-)-methyltransferase NCL1 | -1.07 |
| MAC_02952 | penicillin-binding protein | -1.06 |
| MAC_07532 | fungal specific transcription factor | -1.06 |
| MAC_09601 | hypothetical protein MAC_09601 | -1.06 |
| MAC_07247 | Protein phosphatase 2C domain containing protein | -1.05 |
| MAC_06048 | UDP-glucose,sterol transferase | -1.05 |
| MAC_01221 | MFS monocarboxylate transporter | -1.05 |
| MAC_01796 | alpha-1,2-galactosyltransferase-like protein | -1.05 |
| MAC_08880 | alpha-1,3-mannosyltransferase CMT1 | -1.05 |
| MAC_04965 | hypothetical protein MAC_04965 | -1.05 |
| MAC_03573 | ribose-phosphate pyrophosphokinase | -1.04 |
| MAC_02583 | dimethyladenosine transferase dimethyltransferase | -1.04 |
| MAC_07982 | PAP2 domain protein | -1.04 |
| MAC_05612 | hypothetical protein MAC_05612 | -1.04 |
| MAC_06114 | DEAD box ATP-dependent RNA helicase | -1.03 |
| MAC_07856 | multisynthetase complex auxiliary component p43 | -1.03 |
| MAC_01190 | penicillopepsin | -1.03 |
| MAC_00921 | putative SEN1 protein | -1.03 |
| MAC_01916 | Frequency clock protein | -1.02 |
| MAC_00641 | histone acetyltransferase type B catalytic subunit | -1.02 |
| MAC_05638 | hypothetical protein MAC_05638 | -1.02 |
| MAC_07048 | hypothetical protein MAC_07048 | -1.02 |
| MAC_04102 | increased rDNA silencing protein | -1.01 |
| MAC_01149 | Delta (24(24(1)))-sterol reductase | -1.01 |
| MAC_05222 | peptidyl-tRNA hydrolase domain protein | -1.01 |
| MAC_01302 | norsolorinic acid reductase | -1.01 |
| MAC_06229 | putative lysyl-tRNA synthetase (lysine--tRNA ligase) | -1.01 |
| MAC_08527 | monooxygenase FAD-binding protein | -1.01 |
| MAC_01923 | histone acetyltransferase (MysT1) | -1 |
| MAC_05051 | nuclear export protein Noc3 | -1 |
| MAC_04849 | Superoxide dismutase | 1 |
| MAC_06837 | Rieske [2Fe-2S] domain protein | 1.01 |
| MAC_07355 | GPI-anchored cell wall beta-1,3-endoglucanase EglC | 1.01 |
| MAC_02006 | Lcc2 | 1.03 |
| MAC_05902 | carboxyphosphonoenolpyruvate phosphonomutase | 1.04 |
| MAC_02775 | DNA mismatch repair protein | 1.04 |
| MAC_03517 | hypothetical protein MAC_03517 | 1.04 |
| MAC_04434 | NADP-dependent leukotriene B4 12-hydroxydehydrogenase | 1.05 |
| MAC_00542 | cytochrome c peroxidase precursor | 1.06 |
| MAC_03180 | peroxisomal dehydratase | 1.06 |
| MAC_07788 | copper transporter | 1.07 |
| MAC_07846 | calcium-transporting ATPase 3 | 1.07 |
| MAC_07865 | glucosidase II alpha subunit | 1.12 |
| MAC_03500 | N-acetylglucosamine-6-phosphate deacetylase | 1.12 |
| MAC_00174 | NADP-dependent alcohol dehydrogenase | 1.13 |
| MAC_01993 | Superoxide dismutase | 1.14 |
| MAC_00337 | cytochrome b5 | 1.14 |
| MAC_08573 | C2H2 finger domain protein | 1.15 |
| MAC_02789 | MFS transporter | 1.16 |
| MAC_06282 | N-acetylglucosaminidase | 1.16 |
| MAC_08940 | hypothetical protein MAC_08940 | 1.16 |
| MAC_08402 | Cutinase transcription factor 1 beta | 1.18 |
| MAC_08996 | Ctr copper transporter family protein | 1.19 |
| MAC_07543 | short-chain dehydrogenase | 1.19 |
| MAC_06240 | hypothetical protein MAC_06240 | 1.19 |
| MAC_07310 | homeoprotein | 1.2 |
| MAC_03651 | hypothetical protein MAC_03651 | 1.2 |
| MAC_09685 | short chain dehydrogenase | 1.21 |
| MAC_00549 | WD domain containing protein | 1.21 |
| MAC_03516 | toxin biosynthesis protein | 1.22 |
| MAC_09307 | Ctf1 transcription factor | 1.23 |
| MAC_01091 | hypothetical protein MAC_01091 | 1.23 |
| MAC_09145 | carboxylesterase family protein | 1.26 |
| MAC_04570 | hypothetical protein MAC_04570 | 1.27 |
| MAC_07047 | putative acyl-CoA dehydrogenas | 1.28 |
| MAC_06269 | Peroxidase/catalase | 1.28 |
| MAC_02013 | hypothetical protein MAC_02013 | 1.28 |
| MAC_07651 | fumarate reductase Osm1, putativ | 1.29 |
| MAC_04378 | magnesium-translocating P-type ATPase family protein | 1.29 |
| MAC_05628 | oxidoreductase, short-chain dehydrogenase/reductase family | 1.31 |
| MAC_01466 | vacuolar protease A | 1.33 |
| MAC_06221 | WD-repeat containing protein slp1 | 1.36 |
| MAC_00051 | Calcipressin family protein | 1.36 |
| MAC_09544 | Cutinase gene palindrome-binding protein | 1.36 |
| MAC_02711 | putative calcium P-type ATPase | 1.38 |
| MAC_09005 | subtilisin-like protease Pr1B | 1.38 |
| MAC_01904 | hypothetical protein MAC_01904 | 1.39 |
| MAC_01470 | acyl-CoA dehydrogenase | 1.4 |
| MAC_05152 | quinone oxidoreductase | 1.41 |
| MAC_02332 | ankyrin repeat protein | 1.43 |
| MAC_08742 | flavohemoprotein | 1.43 |
| MAC_05732 | cytochrome P450 | 1.43 |
| MAC_09584 | glyceraldehyde-3-phosphate dehydrogenas | 1.47 |
| MAC_05007 | 4-aminobutyrate aminotransferase | 1.5 |
| MAC_07466 | alpha-glucosidase | 1.51 |
| MAC_02385 | Pyruvate kinase | 1.52 |
| MAC_00424 | hypothetical protein MAC_00424 | 1.52 |
| MAC_08713 | methyltransferase | 1.54 |
| MAC_09442 | long-chain-fatty-acid--CoA ligase FAA2 | 1.55 |
| MAC_09507 | hydrophobin | 1.56 |
| MAC_00274 | enoyl-CoA hydratase/carnithine racemase | 1.57 |
| MAC_09470 | hypothetical protein MAC_09470 | 1.57 |
| MAC_04876 | hypothetical protein MAC_04876 | 1.58 |
| MAC_02839 | mannose-6-phosphate isomerase | 1.66 |
| MAC_01543 | hypothetical protein MAC_01543 | 1.66 |
| MAC_05950 | plasma membrane calcium-transporting ATPase 2 | 1.68 |
| MAC_07309 | putative P-type ATPase | 1.68 |
| MAC_07121 | L-kynurenine/alpha-aminoadipate aminotransferase | 1.73 |
| MAC_03098 | annexin XIV | 1.73 |
| MAC_09498 | aminoadipate-semialdehyde dehydrogenase | 1.73 |
| MAC_07124 | PAP2 domain containing protein | 1.86 |
| MAC_06218 | methyltransferase LaeA | 1.9 |
| MAC_07780 | putative endochitinase CHI3 | 1.9 |
| MAC_08267 | putative BCS1 protein precursor | 1.95 |
| MAC_07120 | cytochrome P450 52A11 | 1.96 |
| MAC_00550 | exo-beta-1,3-glucanase | 1.96 |
| MAC_07329 | hypothetical protein MAC_07329 | 2 |
| MAC_08097 | putative chitosanase CSN1 | 2.04 |
| MAC_06704 | hypothetical protein MAC_06704 | 2.04 |
| MAC_07796 | galactose-proton symport | 2.08 |
| MAC_09405 | sorbitol dehydrogenase | 2.12 |
| MAC_02330 | hypothetical protein MAC_02330 | 2.2 |
| MAC_03586 | putative fluconazole resistance protein (FLU1) | 2.23 |
| MAC_05894 | oxidoreductase | 2.23 |
| MAC_09500 | methyltransferase LaeA | 2.25 |
| MAC_01176 | 3-ketoacyl-CoA thiolase | 2.3 |
| MAC_03585 | cobalamin-independent methionine synthase | 2.34 |
| MAC_04376 | hydrophobin | 2.36 |
| MAC_04169 | HMG box protein | 2.41 |
| MAC_06568 | integral membrane protein | 2.43 |
| MAC_03279 | methyltransferase LaeA | 2.5 |
| MAC_02205 | chitinase 18-15 | 2.55 |
| MAC_08073 | Multifunctional beta-oxidation protein | 2.58 |
| MAC_01059 | Subtilisin-like serine protease PR1A | 2.63 |
| MAC_07558 | major allergen Asp f 2-like protein | 2.65 |
| MAC_09477 | Cytochrome P450 family protein | 2.66 |
| MAC_04396 | hypothetical protein MAC_04396 | 2.75 |
| MAC_08754 | chitinase | 2.94 |
| MAC_01474 | histone H1 | 3.27 |
| MAC_04410 | lysine amidinotransferase | 3.29 |
| MAC_07308 | hypothetical protein MAC_07308 | 3.37 |
| MAC_09497 | polyketide synthase | 3.4 |
| MAC_00831 | D-arabinitol dehydrogenase ArbD | 3.87 |
| MAC_05385 | conidial pigment polyketide synthase PksP/Alb1 | 3.9 |
| MAC_00860 | tetraspanin | 3.91 |
| MAC_06833 | trypsin-related protease | 4.05 |
| MAC_08008 | carboxylesterase family protein | 4.18 |
| MAC_04082 | MFS monocarboxylate transporter | 4.31 |
| MAC_05384 | laccase | 4.9 |
